# Mechanical regulation of adipogenic reprogramming suppresses ovarian cancer progression

**DOI:** 10.1038/s42003-026-10567-4

**Published:** 2026-06-29

**Authors:** Yu-Huan Li, Jia-Wen Zhao, Xin Wang, Zong-Bao Lu, Wei Li, Song-Ling Zhang, Zheng-Quan He

**Affiliations:** 1 Maternity Center, First Hospital of Jilin University, Changchun, Jilin China; 2 State Key Laboratory of Organ Regeneration and Reconstruction, Institute of Zoology, Chinese Academy of Sciences, Beijing, China; 3 Beijing Institute for Stem Cell and Regenerative Medicine, Beijing, China; 4 University of Chinese Academy of Sciences, Beijing, China

**Keywords:** Transdifferentiation, Cancer microenvironment

## Abstract

Epithelial ovarian cancer (EOC) is a lethal malignancy with high recurrence rates owing to residual micrometastases and chemoresistance. This study explores cytoskeletal regulation as a potential therapeutic strategy to modulate tumor cell fate. We demonstrate that combining (‒)-Blebbistatin (Ble) with adipogenic stimuli (BMP4 or PPARγ agonists) effectively induces adipogenic transdifferentiation of ovarian cancer cells both in vitro and in vivo. The transformed cells exhibit characteristic lipid droplet accumulation, upregulated adipogenic markers (*PPARG*, *CEBPA*, and *FABP4*), and functional maturation evidenced by β-adrenergic-responsive lipolysis. In subcutaneous and intraperitoneal models, Ble treatment significantly suppressed tumor growth, with HuNu/Perilipin 1 colocalization confirming human tumor cell conversion into adipocyte-like cells. Mechanistic investigations reveal that cytoskeletal destabilization triggers nuclear heterochromatin redistribution and chromatin decondensation, enhancing accessibility for adipogenic transcription factors (*CEBPA* and *CEBPB*) to activate related transcriptional programs. The critical role of mechanical signaling is demonstrated by both adipogenic conversion on soft substrates and the prevention of Ble-triggered adipogenesis following overexpression of a constitutive nuclear localization mutant of YAP. These findings establish that cytoskeletal destabilization in combination with adipogenic instructive cues enable the adipogenic reprogramming of EOC cells *via* heterochromatin remodeling, providing preclinical insights for ovarian cancer differentiation-targeted therapy.

**Figure:**
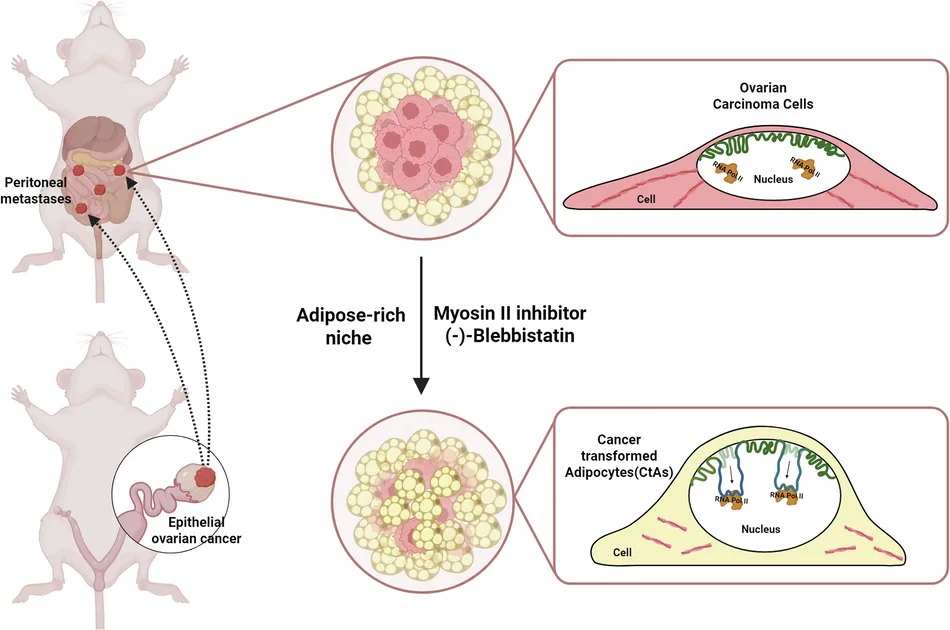

## Abstract (web summary)

Mechanical cytoskeleton disruption combined with adipogenic cues reprograms ovarian cancer cells into adipocyte-like cells via heterochromatin remodeling and YAP nuclear export, suppressing tumor progression.

## Introduction

Epithelial ovarian cancer (EOC) is one of the most lethal gynecological malignancies; approximately 70% of patients are diagnosed at advanced stages (III/IV), and the five-year survival rate is only 30%^1,2^. Current therapeutic strategies for ovarian cancer include surgical resection, chemotherapy, targeted therapy, and immunotherapy, all of which have significant limitations^3,4^. Surgical intervention, while critical for debulking, is highly invasive, and even optimal cytoreduction (R0 resection) may leave behind microscopic metastatic foci (<1 cm)^5^. Platinum-based chemotherapy plus paclitaxel serves as the mainstay adjuvant or neoadjuvant treatment; however, its utility is limited by severe side effects and acquired drug resistance after multiple treatment cycles^6^. Although promising, targeted therapies such as Poly ADP-ribose Polymerase (PARP) inhibitors are effective only for specific molecular subtypes, limiting their broad clinical applicability^7^. Moreover, ovarian cancer is typically classified as an immunologically “cold” tumor because its immunosuppressive microenvironment is characterized by insufficient immune cell infiltration, resulting in poor response rates to immune checkpoint inhibitors^8^. These challenges highlight the urgent need for the development of alternative therapeutic approaches.

Reprogramming the fate of tumor cells has emerged as a promising therapeutic strategy. Unlike conventional approaches that eliminate cancer cells directly (e.g., surgery or chemotherapy) or indirectly (e.g., immune activators), this strategy promotes the transformation of malignant cells into benign non-proliferative cells. This approach minimizes damage to healthy tissues while eradicating the malignant properties of tumor cells, thereby reducing the risk of recurrence and metastasis. Importantly, this strategy is independent of the tumor immune microenvironment, making it potentially effective even for immunologically “cold” tumors like ovarian cancer. Pioneering studies have demonstrated the feasibility of this approach: glioblastoma cells have been successfully reprogrammed into noncancerous neurons *via* overexpression of the neuron-specific transcription factor Neuro D1^9^; however, its application in ovarian cancer remains largely unexplored.

Mechanical signals influence genomic organization and transcription^10,11^. Mesenchymal stem cells exhibit distinct lineage differentiation when attached to substrates of varying stiffness^12^. Importantly, mechanical cues from the tumor microenvironment, such as stromal stiffness, have been shown to reprogram transcriptional networks in cancers^13^. Therefore, regulation of cell fate through mechanical forces represents a strategy for cell fate conversion, which directly affects the physical microenvironment of cells, enabling efficient and safe induction of cell fate conversion with greater potential for clinical translation^14,15^.

Cells sense and respond to extracellular mechanical signals through cytoskeletal regulation^16^. Non-muscle myosin II (NMII) and F-actin filaments together form actomyosin fibers, which generate cytoskeletal tension^17^. These actomyosin fibers receive mechanical signals from the extracellular matrix (ECM) via adhesion molecules and transmit them to the nucleus through the linker of nucleoskeleton and cytoskeleton (LINC) complexes. The nuclear lamina plays a critical role in maintaining chromatin compartmentalization by anchoring heterochromatin to the nuclear periphery, thereby preserving transcriptional silence and stabilizing cell identity^18,19^.

Our previous study demonstrated that disrupting the cytoskeleton of fibroblasts using the NMII inhibitor (‒)-Blebbistatin (Ble) plus neurogenic factors induced efficient transdifferentiation of neurons, establishing a cytoskeletal-microenvironmental coupling paradigm for cell fate reprogramming^20^. Given that both primary and recurrent high-grade serous ovarian carcinomas tend to metastasize to adipose tissue^21–23^, which provides a favorable microenvironment for adipogenic conversion, we hypothesized that a similar approach could be used to reprogram ovarian cancer cells into non-malignant adipocyte-like cells.

Here, we demonstrate that pharmacological disruption of the mechanical cytoskeleton by Ble synergizes with adipogenic stimuli to drive adipogenic transdifferentiation of ovarian cancer cells, concomitantly suppressing their proliferation in vitro and tumorigenicity in vivo. Mechanical cytoskeletal destabilization triggers mechano-epigenetic remodeling through redistribution of constitutive heterochromatin and increased chromatin accessibility, establishing an epigenetically permissive state that facilitates pioneer transcription factor binding. These findings reveal the crucial role of mechanical regulation in determining tumor cell fate and provide a potential strategy for the treatment of metastatic ovarian cancer.

## Results

### Cytoskeleton disruption by Ble facilitates fibroblast transdifferentiation into adipocyte-like cells

**Fig. 1:**
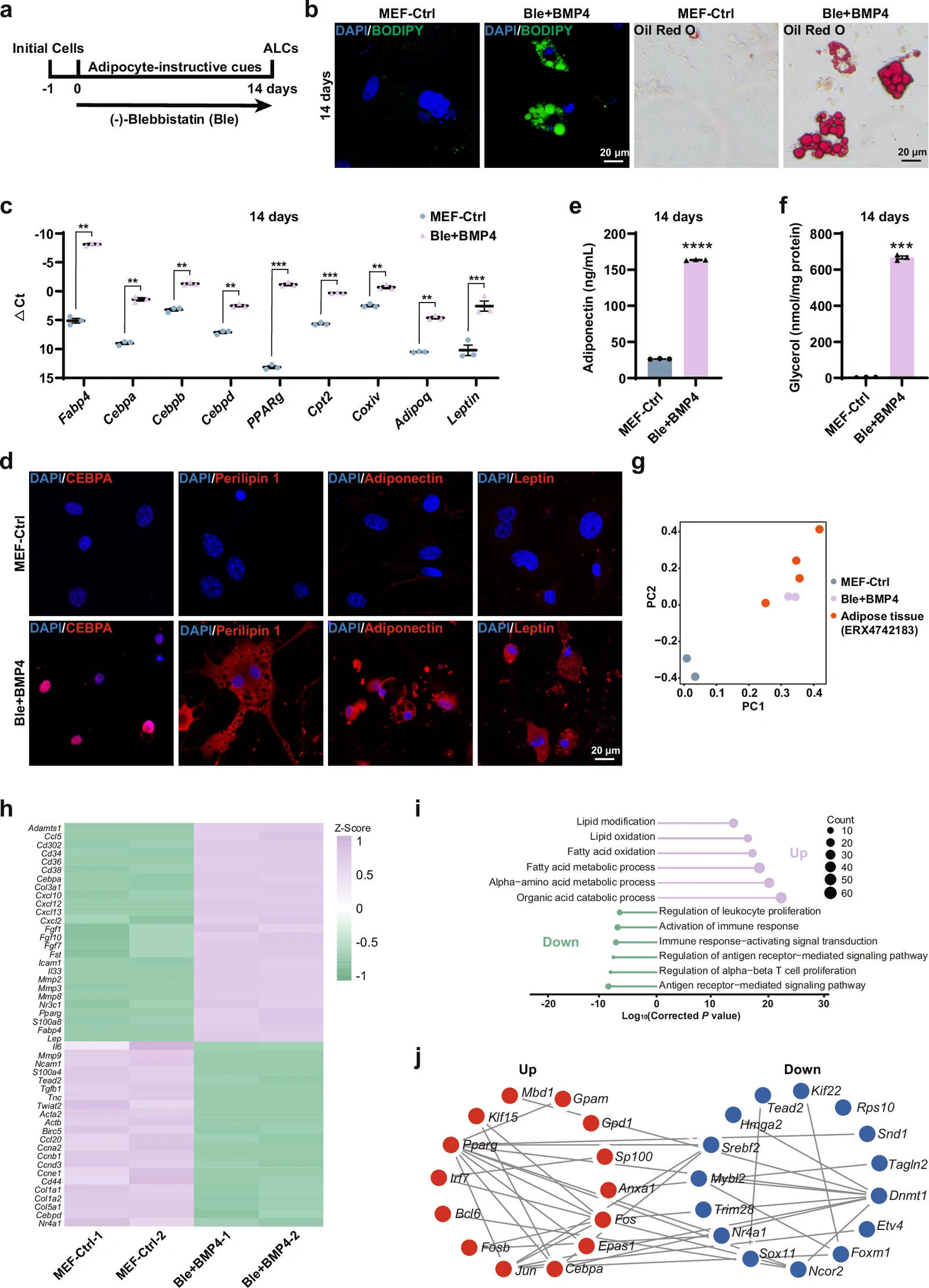
Ble plus adipogenic cues induce transdifferentiation from MEFs to adipocyte-like cells. **a** Schematic of adipogenic transdifferentiation induced by disrupting the cytoskeleton [using (−)-Blebbistatin (Ble)] plus adipocyte-instructive cues (BMP4 or Rosi). **b** Lipid droplets in Ble+BMP4-treated MEFs were visualized by BODIPY and Oil Red O staining. Scale bars, 20 μm. **c** qRT-PCR analysis of ΔCt values (ΔCt = Ct (target gene) – Ct (*Gapdh*)) for key adipogenic transcription factors, mature adipocyte markers, and fatty acid oxidation-related genes in Ble+BMP4-treated MEFs (*n* = 3 independent experiments). Lower ΔCt values indicate higher mRNA expression levels. **d** Immunostaining for CEBPα (red), Perilipin 1(red), Adiponectin (red), Leptin (red), and nuclei (DAPI) in Ble+BMP4-treated MEFs at D14; scale bar, 20 μm. **e** Adiponectin secretion levels were measured for Ctrl and Ble+BMP4-treated MEFs by ELISA (*n* = 3 independent experiments). **f** Isoproterenol-induced lipolysis was measured in Ble+BMP4-treated MEFs (*n* = 3 independent experiments). A colorimetric analysis was used to determine glycerol release (lipolysis). **g** Principal component analysis (PCA) of the transcriptomes of adipose tissue, Ctrl and Ble+BMP4-treated MEFs. RNA-seq data of mouse adipose tissue was downloaded from the Sequence Read Archive database with data accession number ERX4742183^54,55^. **h** Heatmap of selected adipocyte-specific and MEF-specific gene expression in Ble+BMP4-treated MEFs. **i** Gene ontology (GO) analysis of genes upregulated and downregulated in Ble+BMP4-treated MEFs compared with Ctrl group. The dot size represents the gene count. **j** Transcription factor networks for Ble+BMP4-treated MEFs. Red and blue nodes indicate upregulation and downregulation, respectively. Data are presented as mean ± SEM. **P < 0.01, ***P < 0.001, ****P < 0.0001. Statistical analyses for (**c**,** e**,** f**) were performed using two-sided paired Student’s *t* tests. For qRT-PCR data (**c**), paired two-sided *t*-tests were applied to ΔCt values, and multiple testing correction across genes was performed using the two-stage step-up method (Benjamini, Krieger, and Yekutieli; FDR = 5%).

To establish a method of adipogenic transdifferentiation induced by cell mechanical destruction, we treated mouse embryo fibroblasts (MEFs) with Ble in adipose environment which add adipocyte-instructive cues, such as BMP4 and Rosiglitazone (Rosi)^24–26^ (Fig. 1a). BODIPY and Oil Red O staining revealed robust lipid droplet accumulation in Ble+BMP4-treated MEFs (Fig. 1b), indicating the presence of adipocyte-like cells^27^. In contrast, no lipid droplets were observed after BMP4 treatment alone, highlighting the indispensable role of Ble in driving adipogenic conversion (Supplementary Fig. 1a, b).

To characterize these adipocyte-like cells, we analyzed their gene expression and found that Ble+BMP4 treatment activated an adipogenic transcriptional program, increasing the expression of key adipogenic regulators (*Cebpa*, *Cebpb*, *Cebpd*, *and Pparg*) and the adipocyte markers (*Fabp4*, *Adipoq*, and *Leptin*)^28^. Additionally, genes involved in fatty acid oxidation (*Cpt2*) and mitochondrial respiration (*Coxiv*) were also markedly increased, suggesting increased metabolic activity in the transdifferentiated cells (Fig. 1c and Supplementary Fig. 1c). Immunofluorescence staining further confirmed the induction of core adipogenic regulators (CEBPA) and functional markers (Perilipin 1, Adiponectin, and Leptin) (Fig. 1d and Supplementary Fig. 1d, f), validating activation of the adipogenic program at the protein level.

Next, we evaluated the functional properties of these reprogrammed MEFs. Consistent with their adipocyte identity, Ble+BMP4-treated cells secreted higher levels of adiponectin (Fig. 1e), and exhibit the physiological function of adipocytes by executing lipolysis in response to β-adrenergic stimulation with isoproterenol (Fig. 1f), confirming their capacity to regulate energy mobilization in response to physiological stimuli^29^. Notably, in contrast to Ble+BMP4-treated MEFs, MEFs treated with BMP4 alone exhibited no detectable induction of adipocyte-specific functional characteristics (Supplementary Fig. 1h, i). Together, these findings clearly demonstrate that Ble+BMP4-treated MEFs acquire the functional and metabolic hallmarks required for terminal adipocyte maturation, confirming their biological fidelity to native adipocytes^30^.

Transcriptomic profiling *via* bulk RNA-seq further corroborated these observations. Principal component analysis showed that Ble+BMP4-treated MEFs clustered within primary adipocytes from adipose tissues, clearly distinct from MEFs (origin cells) (Fig. 1g). Differential gene expression profiling further supported this finding, showing robust upregulation of adipogenic markers (e.g., *Cebpa*, *Pparg*, and *Fabp4*) (Fig. 1h). Gene cluster analysis revealed that the genes upregulated in Ble+BMP4-reated MEFs were predominantly associated with adipocyte bioprocesses, including lipid modification, oxidation, and fatty acid metabolism (Fig. 1i). Transcriptional regulatory network analysis identified *Pparg* and *Cebpa* as the core upregulated transcription factors (Fig. 1j). Notably, both differential expression gene profiling and transcriptional regulatory network analysis revealed downregulation of cell cycle-related genes (e.g., *Ccna2*, *Ccnb1*, *Ccnd3*, *Tead2*, *Foxm1*, *and Ncor2*) (Fig. 1h, j), confirming the proliferation arrest observed in Ble+BMP4-treated MEFs. Together, these results demonstrated that Ble facilitates MEFs to undergo adipogenic transdifferentiation in adipogenesis culture system.

Finally, we substituted BMP4 with the pharmacological PPARγ agonist Rosiglitazone to recapitulate an adipogenic niche^31,32^. Comprehensive molecular profiling and functional analyses revealed a robust commitment to the adipose lineage (Supplementary Fig. 2). These findings demonstrated that synchronous cytoskeleton disruption and adipogenic stimulation synergistically reprogram MEFs into metabolically competent adipocyte-like cells.

### Ble facilitates adipogenic transdifferentiation and suppresses proliferation of ovarian cancer cells in vitro

**Fig. 2:**
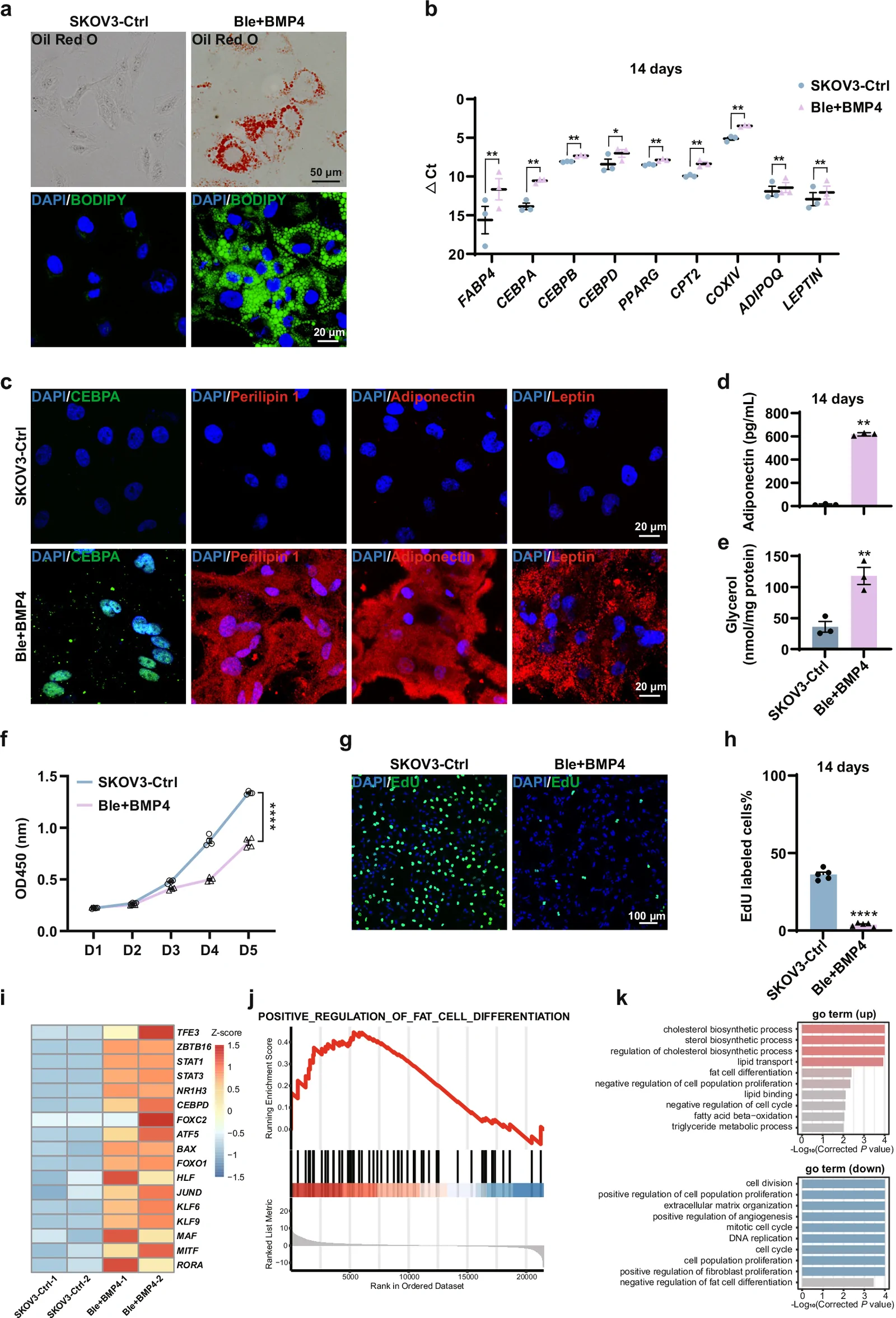
Ble plus adipogenic cues induce SKOV3 adipogenic transdifferentiation and inhibit proliferation in vitro. **a** Lipid droplets in Ble+BMP4 treated SKOV3 cells at D14 were visualized by Oil Red O and BODIPY staining. Scale bars, 50 μm and 20 μm. **b** qRT-PCR analysis of ΔCt values (ΔCt = Ct (target gene) – Ct (*GAPDH*)) for adipocyte-specific genes in Ble+BMP4 treated SKOV3 cells at D14 (*n* = 3 independent experiments). Lower ΔCt values indicate higher mRNA expression levels. **c** Immunostaining for CEBPα (green), Perilipin 1 (red), Adiponectin (red), Leptin (red), and nuclei (DAPI) in Ble+BMP4 treated SKOV3 cells at D14; scale bar, 20 μm. **d** Adiponectin secretion levels were measured for Ctrl and Ble+BMP4 treated SKOV3 cells by ELISA (*n* = 3 independent experiments). **e** Isoproterenol-induced lipolysis was measured in Ble+BMP4 treated SKOV3 cells at D14 (*n* = 3 independent experiments). **f** CCK-8 analysis of Ble+BMP4 treated SKOV3 cells revealed a lower cell proliferation rate (*n* = 4 independent experiments). **g**, **h** Ble+BMP4 treated SKOV3 cells were incubated with EdU for 2.5 h to label proliferating cells. EdU-labeled cells were quantified using ImageJ (*n* = 5 independent experiments), scale bar, 100 μm. **i** Heatmap illustrating the expression patterns of key adipogenesis-related transcription factors in Ctrl and Ble+BMP4 groups. **j** GSEA enrichment plot showing borderline enrichment of the “positive regulation of fat cell differentiation” gene set in SKOV3 cells upon Ble+BMP4 treatment (NES: 1.5132473, FDR: 0.0782314). **k** GO enrichment analysis showing selected biological processes for upregulated and downregulated genes in Ble+BMP4 treated SKOV3 cells. Data are presented as mean ± SEM. *P < 0.05, **P < 0.01, ****P < 0.0001. Statistical analyses for (**b**, **d**, **e**, **h**) were performed using two-sided paired Student’s *t* tests. For qRT-PCR data (**b**), paired two-sided *t*-tests were applied to ΔCt values, and multiple testing correction across genes was performed using the two-stage step-up method (Benjamini, Krieger, and Yekutieli; FDR = 5%). For (**f**), proliferation rates were derived from linear regression of the growth curves, and significance between groups was calculated using an unpaired two-sided Student’s *t* test on the regression slopes.

Having established a proof-of-concept for adipogenic transdifferentiation using Ble plus BMP4 or Rosi in MEFs, we explored its translational potential in human ovarian carcinoma cells, including SKOV3, A2780 and OVCAR3 cells. Strikingly, BODIPY and Oil Red O staining revealed robust intracellular lipid droplet accumulation in human ovarian carcinoma cells after 14 days of Ble+BMP4 treatment (Fig. 2a, Supplementary Figs. 3a, b and 4a), indicating adipocyte fate commitment. Gene expression analysis further demonstrated significant upregulation of adipocyte-specific genes, including *FABP4*, *CEBPA*, and *PPARG* (Fig. 2b, Supplementary Figs. 3c and 4b). Notably, immunofluorescence staining confirmed the induction of core adipogenic regulators (CEBPA) and mature adipocyte markers (Perilipin 1, Adiponectin, and Leptin) in Ble+BMP4-treated human ovarian carcinoma cells (Fig. 2c, Supplementary Figs. 3d, e and 4c). Consistent with these findings, functional assays revealed significantly elevated adiponectin secretion (Fig. 2d, Supplementary Figs. 3f and 4d) and increased lipolytic activity in response to the β-adrenergic agonist isoproterenol (Fig. 2e, Supplementary Figs. 3g and 4e) in Ble+BMP4-treated human ovarian carcinoma cells, confirming that these cancer-derived adipocyte-like cells acquire key physiological characteristics of mature adipocytes. Collectively, these results suggest that Ble+BMP4 treatment drives the transdifferentiation of ovarian cancer cells into functional adipocyte-like cells.

To further delineate the molecular program underlying this cell fate transition, transcriptomic profiling was performed. Analysis of key adipogenesis-related transcription factors via heatmap exhibited distinct upregulation of core regulators, such as *CEBPD* and *PPARG*, in Ble+BMP4-treated human ovarian carcinoma cells (Fig. 2i, Supplementary Figs. 3j and 4i). Consistently, Gene Set Enrichment Analysis (GSEA) suggested a borderline enrichment of the gene signature associated with positive regulation of fat cell differentiation (Fig. 2j). Consistently, Gene Ontology (GO) enrichment analysis further validated that genes upregulated in Ble+BMP4-treated cells were primarily involved in adipocyte-specific biological processes, including cholesterol biosynthesis, lipid transport, and triglyceride metabolism (Fig. 2k, Supplementary Figs. 3k and 4j). Collectively, these multi-omics results provide compelling molecular evidence confirming the acquisition of a mature adipocyte transcriptional program.

We subsequently investigated the effect of adipogenic transdifferentiation on the proliferative properties of human ovarian carcinoma cells. CCK-8 assays demonstrated that Ble+BMP4 treatment significantly inhibited the proliferation of these cells (Fig. 2f, Supplementary Figs. 3h and 4f). This antiproliferative effect was further confirmed through multiple approaches. 5-Ethynyl-2′-deoxyuridine (EdU) incorporation assays revealed a significant decrease in DNA synthesis, as evidenced by the reduced number of EdU-labeled cells in Ble+BMP4-treated cultures compared to that in Ctrl cultures (Fig. 2g, h, Supplementary Figs. 3i and 4g, h). Consistent with the antiproliferative phenotype observed in vitro, GO enrichment analysis of downregulated genes revealed a strong enrichment of pathways associated with cell cycle progression, mitotic nuclear division, and DNA replication (Fig. 2k, Supplementary Figs. 3l and 4k). Collectively, these findings demonstrate that Ble treatment in combination with adipogenic cues induce transdifferentiation of ovarian cancer cells into functional adipocyte-like cells and arrests their proliferation, supporting the potential of Ble as a cancer therapeutic strategy.

### Ble inhibits tumor growth and facilitates adipogenic transdifferentiation of ovarian cancer cells in vivo

**Fig. 3:**
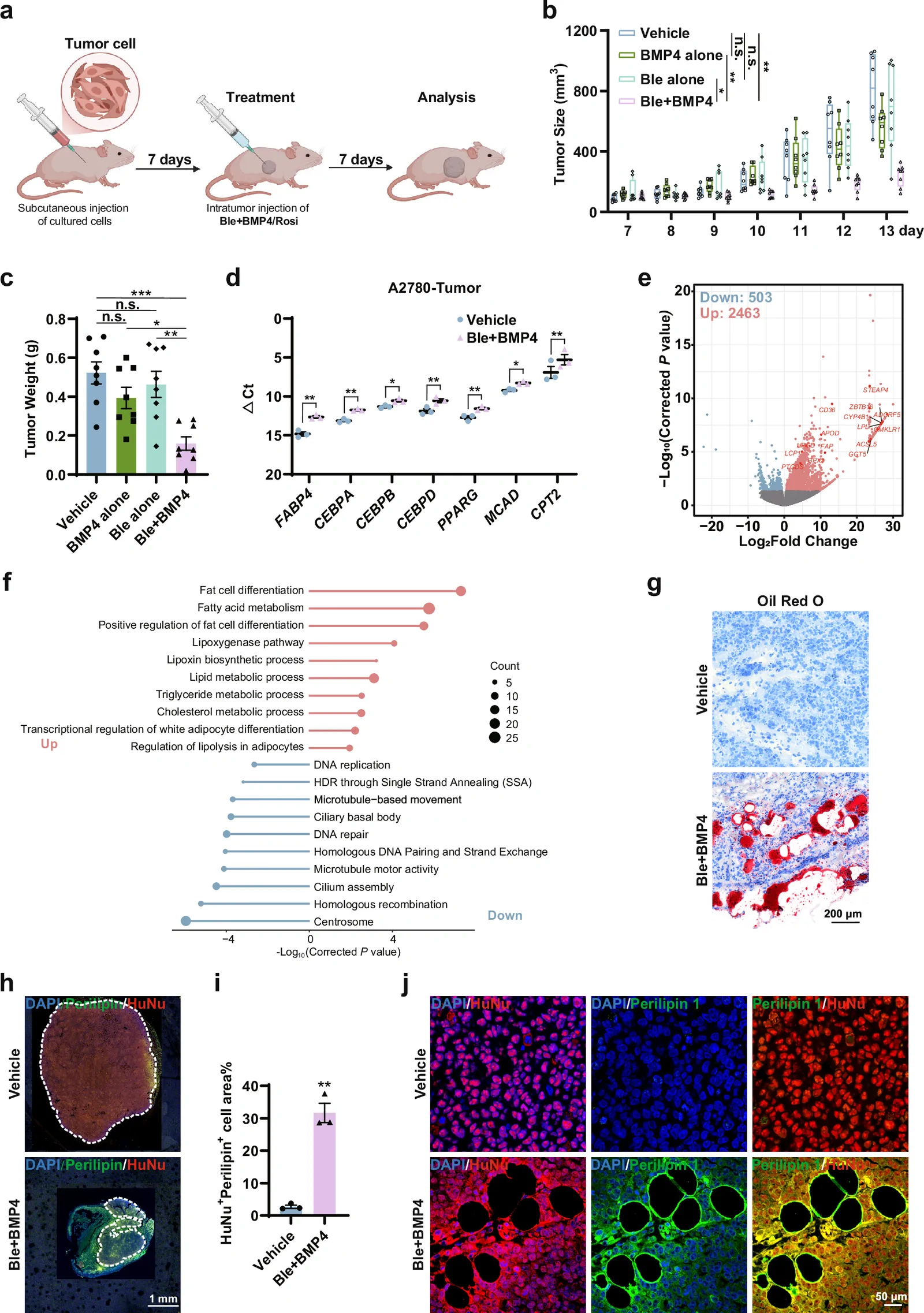
Ble plus adipogenic cues inhibit tumor growth and induce adipogenic transdifferentiation of A2780 cells in vivo. **a** Schematic diagram of subcutaneous tumor-bearing mice administration^56^. **b** Tumor volume growth curves are shown during the treatment time course (*n* = 8 animals for each group, *P* = 0.2063 for Vehicle vs BMP4 alone, *P* = 0.8240 for Vehicle vs Ble alone, *P* = 0.0019 for Vehicle *vs* Ble+BMP4, *P* = 0.0113 for Ble alone vs Ble+BMP4, *P* = 0.0013 for BMP4 alone vs Ble+BMP4). **c** Tumor weights are shown after 7 days of treatment (*n* = 8 animals). **d** qRT-PCR analysis of ΔCt values (ΔCt = Ct (target gene) – Ct (*GAPDH*)) for adipocyte-specific genes in subcutaneous tumors from Ble+BMP4 treated mice (*n* = 3 biologically independent samples). Lower ΔCt values indicate higher mRNA expression levels. **e** Volcano plot of differentially expressed genes (DEGs) between the Ctrl and Ble+BMP4 treated groups. The red dots indicate significantly upregulated genes (log_2_Fold Change > 1, Corrected *P* < 0.05), whereas the blue dots represent significantly downregulated genes (log_2_Fold Change < −1, Corrected *P* < 0.05). The gray dots denote nonsignificant genes. The adipocyte-specific genes are labeled. **f** Bar plot of selected functional enrichment terms of genes upregulated and downregulated in Ble+BMP4 treated mouse tumors. The dot size represents the gene count. **g** Representative images of tumor sections stained with Oil Red O. Scale bars, 200 μm. **h** Representative images of whole tumor sections that were stained with HuNu (red) and Perilipin 1 (green). The white dotted line encircles the boundary of the tumor. Scale bars, 1 mm. **i** The area occupied by HuNu and Perilipin 1 copositive cells was quantified (*n* = biologically independent samples). **j** Local zoom images of tumor sections that were stained with HuNu (red) and Perilipin 1 (green). Scale bars, 50 μm. Data are presented as mean ± SEM. *P < 0.05, **P < 0.01, ***P < 0.001, ****P < 0.0001. n.s. no significant difference (*P* > 0.05). For **b** (tumor growth rates derived from linear regression of individual tumor volume curves) and **c** (tumor weights), data were analyzed by Welch’s ANOVA with Dunnett’s T3 (**b**, unequal variances) or ordinary one-way ANOVA with Tukey’s test (**c**, equal variances). All four groups (Vehicle, BMP4 alone, Ble alone, Ble+BMP4) were compared pairwise. For qRT-PCR data (**d**), paired two-sided *t*-tests were applied to ΔCt values, and multiple testing correction across genes was performed using the two-stage step-up method (Benjamini, Krieger, and Yekutieli; FDR = 5%). For (**i**), a two-sided paired Student’s *t* test was used.

To determine whether adipogenic transdifferentiation could inhibit ovarian cancer progression in vivo, we established a subcutaneous xenograft model using A2780 cells. The mice were administered intratumoral and paratumoral injections starting 7 days after tumor inoculation, and the tumors were harvested for analysis after another 7 days (Fig. 3a). First, Ble plus with BMP4 or Rosi treatment significantly reduced both tumor volume and weight, with a noticeable difference in tumor size between the Ctrl and Ble+BMP4 or Ble+Rosi treated groups (Fig. 3b, c and Supplementary Fig. 5a, b). Gene clustering analysis also revealed that the downregulated genes were primarily associated with pathways such as DNA replication and DNA repair, further confirming that Ble+BMP4 or Ble+Rosi treatment suppressed tumor proliferation (Fig. 3f).

To investigate whether Ble treatment facilitates adipogenic transdifferentiation of tumor cells in vivo, we examined gene expression in subcutaneous tumors and found a significant upregulation of the expression in adipocyte-specific markers, including *CEBPA*, *CEBPB*, *CEBPD*, and *PPARG*, in the Ble+BMP4 or Ble+Rosi treated group (Fig. 3d and Supplementary Fig. 5c). RNA-seq analysis also revealed 2463 upregulated genes, with adipocyte-specific genes such as *CD36*, *LPL*, and *ACSL5* prominently upregulated (Fig. 3e). Furthermore, gene clustering analysis revealed that the upregulated genes were enriched in pathways related to fat cell differentiation, fatty acid metabolism, and fat cell differentiation, suggesting the induction of adipogenic fate in tumor cells. (Fig. 3f). Notably, Oil Red O staining of subcutaneous tumors revealed significant staining at the tumor periphery and injection sites, indicating adipogenic transdifferentiation in these regions (Fig. 3g and Supplementary Fig. 5d). More rigorously, by labeling human-derived ovarian cancer cells with anti-human nuclear antigen (anti-HuNu), we demonstrated its colocalization with the adipocyte-associated protein Perilipin 1 (Fig. 3h‒j and Supplementary Fig. 5e, f). These findings provide strong evidence that subcutaneously inoculated human ovarian carcinoma cells successfully transdifferentiate into adipocyte-like cells in vivo following Ble plus with BMP4 or Rosi treatment.

### Ble facilitates adipogenic transdifferentiation of ovarian cancer peritoneal micrometastases in the endogenous adipose niche

**Fig. 4:**
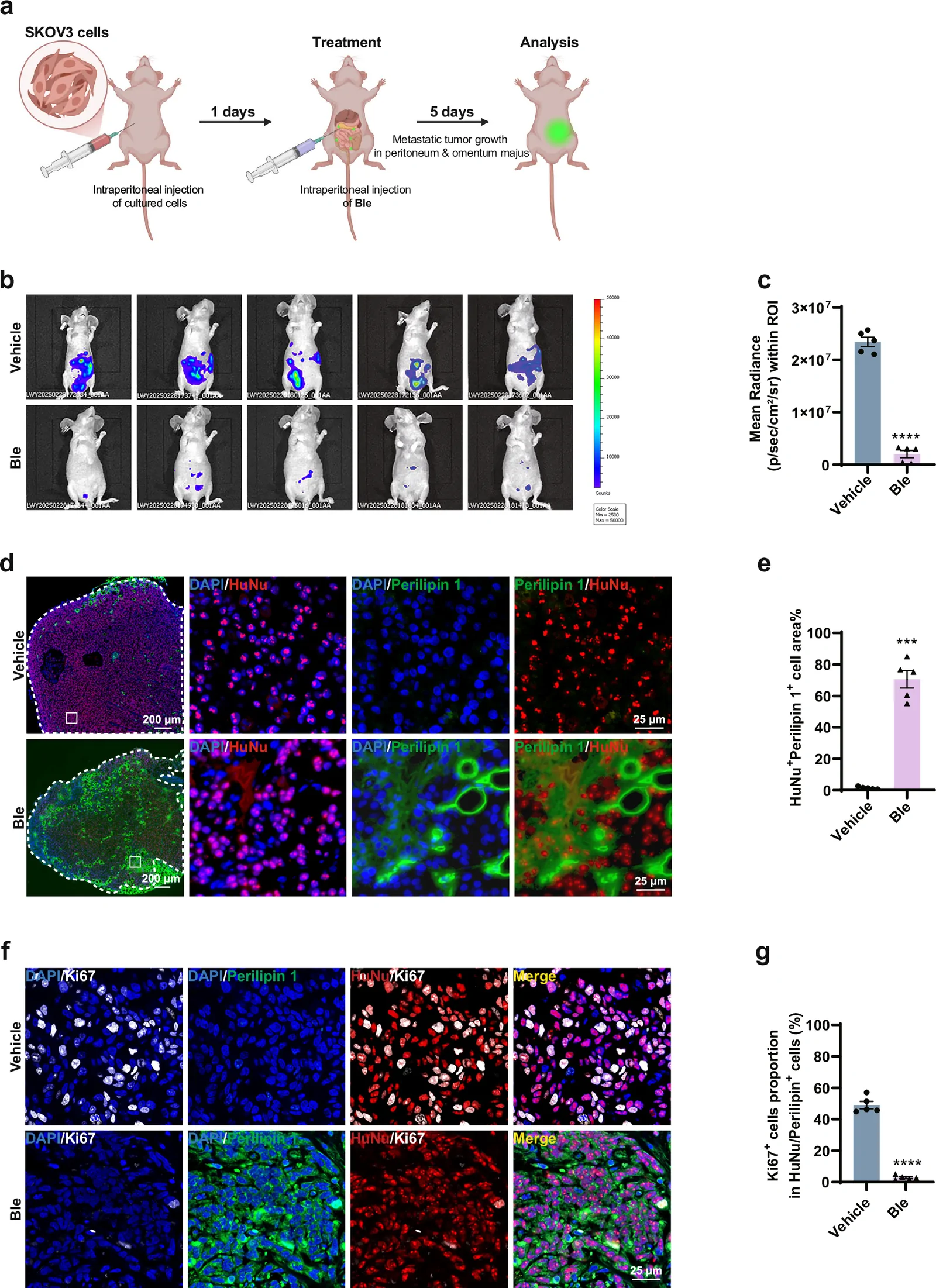
Ble inhibits ovarian cancer peritoneal micrometastases growth and facilitates adipogenic transdifferentiation in the peritoneal adipose niche. **a** Schematic diagram of drug administration in a mouse model of peritoneal metastatic ovarian cancer^57^. **b** Representative bioluminescence images of vehicle and Ble treatment mice at D5 after drug administration. **c** Quantification of tumor radiance (*n* = 5 animals) for (**b**). **d** Representative images of tumor sections that were stained with HuNu (red) and Perilipin 1 (green). The white dotted line encircles the boundary of the tumor. Scale bars, 200 μm and 25 μm. **e** The area occupied by HuNu and Perilipin 1 co-positive cells was quantified (*n* = 5 biologically independent samples). **f** Representative images of tumor sections that were stained with HuNu (red), Ki67 (white), and Perilipin 1 (green). Scale bars, 25 μm. **g** The Ki67-positive cells fraction in HuNu and Perilipin 1 co-positive cells was quantified (*n* = 5 biologically independent samples). Random visual fields were selected from each group to avoid artificial selection. Data are presented as mean ± SEM. ****P < 0.0001. For **c**, a two-sided unpaired Student’s *t* test was used. For (**e**, **g**), two-sided paired Student’s *t* tests were applied.

To investigate whether Ble treatment can facilitate adipogenic transdifferentiation of tumor cells within an endogenous adipose environment, a context that closely mirrors the clinical scenario of intraperitoneal metastatic ovarian cancer, we established a murine model of intraperitoneal ovarian cancer metastasis to mimic metastatic ovarian cancer in a clinical setting. Intraperitoneal drug administration was initiated on the second day after the tumor inoculation (Fig. 4a). The results revealed a significant reduction in the tumor burden in the Ble-treated group as early as D5, as visualized by intravital imaging (Fig. 4b, c), consistent with observations in the OVCAR3 model at D10, where Ble treatment significantly reduced both the number of intraperitoneal tumor nodules and total tumor volume compared to vehicle controls (Fig. Supplementary Fig. 6a, b), suggesting that Ble treatment inhibits the proliferation of intraperitoneally disseminated ovarian cancer cells.

Similarly, we also observed colocalization of HuNu and Perilipin 1 in tumor sections harvested from the peritoneal cavity of mice treated with Ble alone (Fig. 4d, e and Supplementary Fig. 6c), indicating that peritoneally inoculated SKOV3 and OVCAR3 cells underwent adipogenic transdifferentiation under Ble treatment. Consistent with the immunofluorescence findings, qRT-PCR analysis of OVCAR3-derived tumors at D10 revealed significant upregulation of key adipogenic genes in the Ble-treated group (Fig. Supplementary Fig. 6e), providing molecular evidence of adipogenic transdifferentiation in vivo. More importantly, we further observed a significant reduction in the proportion of Ki67-positive cells among HuNu and Perilipin 1 co-positive cells, indicating that adipogenic transdifferentiated tumor cells ceased proliferation (Fig. 4f, g and Supplementary Fig. 6c, d), which corroborates the decreased tumor burden observed *via* intravital imaging and the reduced tumor nodule count and volume in the Ble-treated group.

### Ble enhances chromatin accessibility to facilitate adipogenic transcription factor activation

**Fig. 5:**
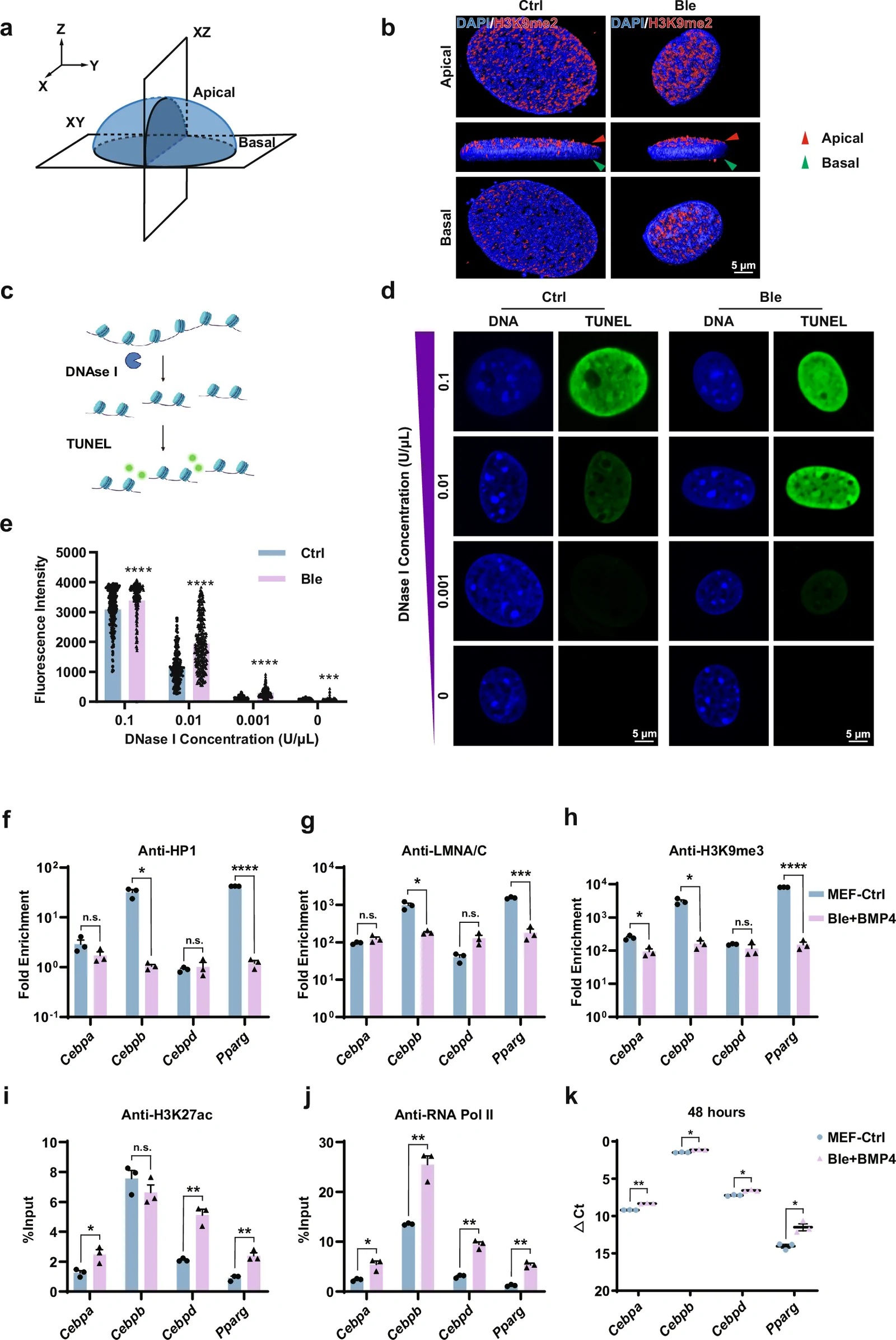
Ble treatment increases DNA accessibility to support adipogenic fate conversion. **a** 3D diagram of the cell nucleus. **b** Representative images of nuclear H3K9me2 immunofluorescence staining showing a reduced apical distribution of H3K9me2 after Ble treatment for 48 h. Scale bars, 5 μm. **c** Schematic illustration of the DNase I-TUNEL assay^58^. **d** After Ble treatment for 48 h, TUNEL signal of cells treated with different concentrations of DNase I. **e** Quantification of TUNEL signal fluorescence intensity under different DNase I concentrations in Ctrl and Ble-treated groups. Cell counts per independent culture (*n*) are as follows: Ctrl: 281, 321, 242, 254; Ble: 193, 346, 308, 257, corresponding to DNase I concentrations of 0.1, 0.01, 0.001, and 0 U/µL, respectively. **f**, **g** ChIP-qPCR analysis revealed a decrease in HP1 and LMNA/C enrichment at the promoters of the adipocyte-specific transcription factors *Cebpb* and *Pparg* after Ble treatment for 48 h (*n* = 3 independent experiments). **h** ChIP-qPCR analysis revealed a decrease in H3K9me3 enrichment at the promoters of the adipocyte-specific transcription factors *Cebpa*, *Cebpb*, and *Pparg* after Ble treatment for 48 h (*n* = 3 independent experiments). **i** ChIP-qPCR analysis revealed an increase in H3K27ac enrichment at the promoters of *Cebpa*, *Cebpd*, and *Pparg* after Ble treatment for 48 h (*n* = 3 independent experiments). **j** ChIP-qPCR analysis revealed an increase in RNA Pol II enrichment at the promoters of *Cebpa*, *Cebpb*, *Cebpd*, and *Pparg* after Ble treatment for 48 h (*n* = 3 independent experiments). **k** qRT-PCR analysis of ΔCt values (ΔCt = Ct (target gene) – Ct (*Gapdh*)) for *Cebpa*, *Cebpb*, *Cebpd*, and *Pparg* in Ble-treated cells (*n* = 3 independent experiments). Lower ΔCt values indicate higher mRNA expression levels. Data are presented as mean ± SEM. *P < 0.05, **P < 0.01, ***P < 0.001, ****P < 0.0001. n.s., no significant difference (*P* > 0.05). For **e**, at each DNase I concentration, paired *t*-tests were performed between Ctrl and treatment groups; for (**f**–**k**), paired *t*-tests were applied to account for inter-batch variation. Multiple testing correction across all panels was performed using the two-stage step-up method (Benjamini, Krieger, and Yekutieli; FDR = 5%).

Cytoskeletal dysfunction leads to nuclear softening, which may cause redistribution of constitutive chromatin^19^. To elucidate the mechanism underlying Ble-induced adipogenesis in the adipose environment, we systematically analyzed a series of biological events that occur during the early stages of adipogenesis. First, we performed 3D reconstruction of the distribution of H3K9me2 in the nucleus^33^. The results revealed that H3K9me2 was predominantly localized on the apical side in untreated cells, but began to shift toward the basal side in Ble-treated cells, suggesting the release of transcriptionally repressed heterochromatin regions, indicating remolded heterochromatin organization (Fig. 5a, b). To investigate whether changes in H3K9me2 distribution correlated with altered chromatin condensation, we performed an in-situ DNase I–Terminal deoxynucleotidyl transferase dUTP nick end labeling (TUNEL) assay in which double-strand breaks induced by DNase I treatment were detected using TUNEL assays (Fig. 5c). The fluorescence intensity measured by TUNEL assay serves as a direct indicator of DNase I sensitivity and, consequently, the degree of chromatin condensation^34^. Our results revealed that Ble treatment significantly increased TUNEL fluorescence intensity compared to that in Ctrl cells (Fig. 5d, e), suggesting that chromatin decondensation, which may increase chromatin accessibility, facilitates cellular reprogramming.

Next, we examined the binding of adipogenic transcription factors (*Cebpa*, *Cebpb*, *Cebpd*, and *Pparg*) to the heterochromatin marker Heterochromatin protein 1 (HP1) and the nuclear lamina protein LMNA/C in this permissive chromatin environment by ChIP-qPCR. The results showed that the binding of *Cebpb* and *Pparg* to both HP1 and LMNA/C was significantly reduced after 48 h of Ble treatment (Fig. 5f, g), indicating that the transcriptionally silent state maintained by the nuclear lamina was disrupted, allowing the expression of adipogenic genes. Subsequently, we examined the epigenetic status of key adipogenic transcription factors by ChIP-qPCR and found that the epigenetic state of adipogenic transcription factors (*Cebpa*, *Cebpb*, *Cebpd* and *Pparg*) was altered, characterized by a reduction in the repressive marker H3K9me3 and an increase in the activation marker H3K27ac (Fig. 5h, i). Furthermore, we observed a significant increase in the binding of these transcription factors to RNA polymerase II, indicating increased transcriptional activity (Fig. 5j) along with increased mRNA levels of these transcription factors (Fig. 5k), further confirming the activation of the adipogenic transcriptional network. Collectively, these data suggest that Ble-mediated cytoskeletal disruption alters the heterochromatin distribution and increases global chromatin accessibility. In an adipose environment, the activity of pioneer transcription factors is further increased, thereby promoting the initiation of adipogenesis.

### Mechanical signals facilitate adipogenic transdifferentiation

**Fig. 6:**
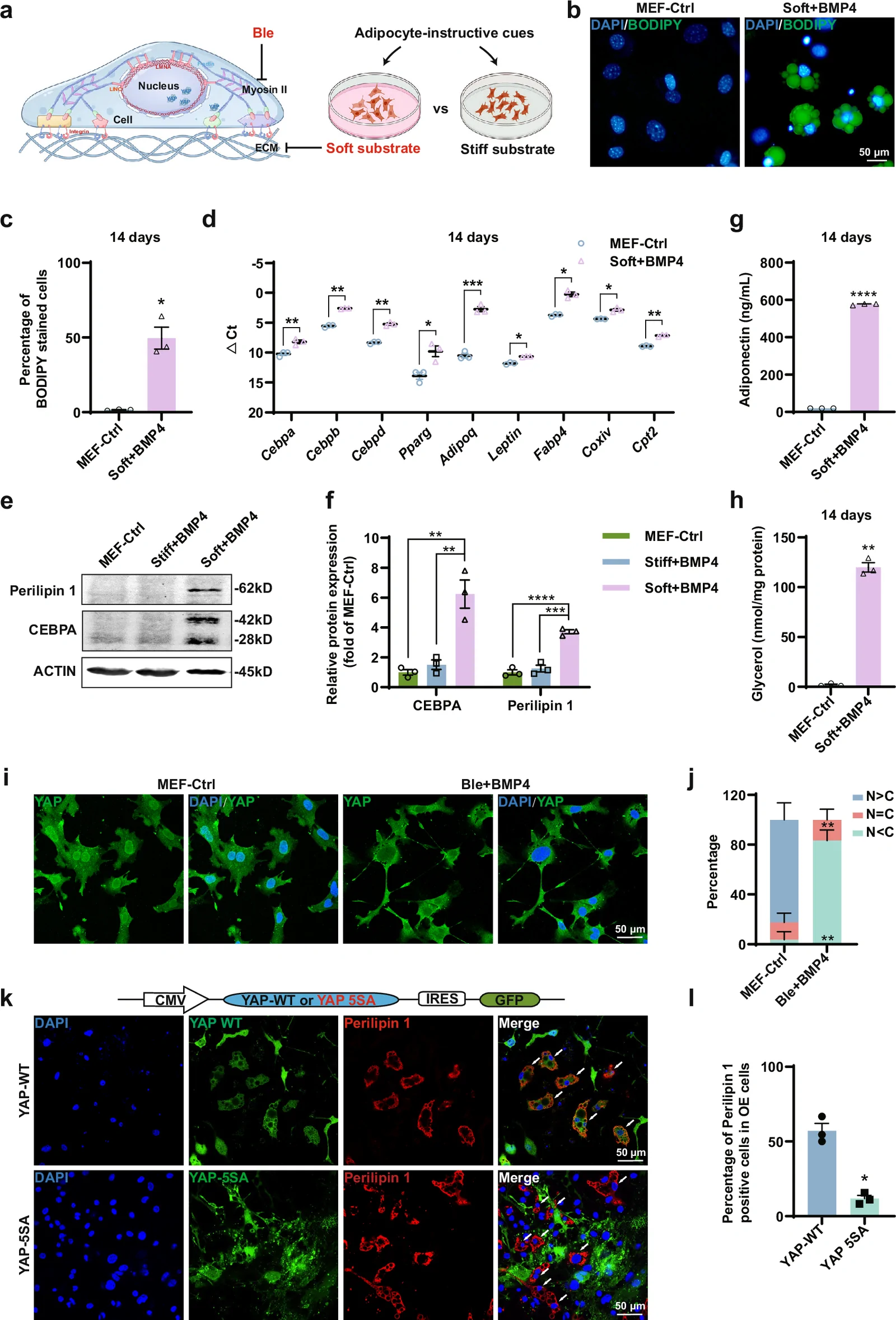
Mechanical signals facilitate adipogenic transdifferentiation in a YAP-dependent manner. **a** Schematic of adipogenic transdifferentiation induced by soft substrate culture combined with adipocyte-instructive cues (BMP4) treatment. **b** Lipid droplets of MEFs cultured on stiff (Ctrl) versus soft substrates with BMP4 for 14 days were visualized by BODIPY staining. Scale bars, 50 μm. **c** The percentage of BODIPY-stained cells was quantified (*n* = 3 independent experiments). **d** qRT-PCR analysis of ΔCt values (ΔCt = Ct (target gene) – Ct (*GAPDH*)) for adipogenesis- and FAO-related genes in MEFs cultured on soft substrates with BMP4 (*n* = 3 independent experiments). Lower ΔCt values indicate higher mRNA expression levels. **e** Western blot analysis of CEBPα and Perilipin 1 in MEFs cultured on soft substrates with BMP4 for 14 days. **f** Quantitative analysis of **g**, the relative protein expression of CEBPα and Perilipin 1 (*n* = 3 independent experiments). **g** Adiponectin secretion levels were measured for MEFs cultured on soft substrates with BMP4 by ELISA (*n* = 3 independent experiments). **h** Isoproterenol-induced lipolysis was measured in MEFs cultured on soft substrates with BMP4 for 14 days (*n* = 3 independent experiments). **i** Immunocytochemistry of YAP subcellular localization in Ble+BMP4 treated cells. Scale bar, 50 μm. **j** YAP subcellular localization percentage was quantified (*n* = 3 independent experiments). N Nucleus, C Cytoplasm. **k** Representative immunofluorescence images of Ble-treated MEFs overexpressing YAP-WT or YAP-5SA (green), stained for Perilipin 1 (red). Scale bar, 50 μm. **l** Quantification of the percentage of Perilipin 1-positive cells in MEFs overexpressing YAP-WT or YAP-5SA (*n* = 3 independent experiments). Data are presented as mean ± SEM. *P < 0.05, **P < 0.01, ***P < 0.001, ****P < 0.0001. For **c**, **g**, **h**, **i**, paired *t*-tests were applied. For qRT-PCR data (**d**), paired two-sided *t*-tests were applied to ΔCt values, and multiple testing correction across genes was performed using the two-stage step-up method (Benjamini, Krieger, and Yekutieli; FDR = 5%). For **f**, one-way ANOVA with Dunnett’s test was used after normalization to MEF-Ctrl. For **j**, paired *t*-tests were performed using the two-stage step-up method (Benjamini, Krieger, and Yekutieli; FDR = 5%).

NM II-based actomyosin stress fibers serve as critical mechanotransduction components that perceive mechanical cues from the ECM and transmit them *via* the LINC complex to the nuclear lamina, thereby facilitating the mechanical regulation of nuclear processes^35^ (Fig. 6a). To further investigate the influence of mechanical signaling on cell fate conversion, we cultured MEFs on soft substrates designed to mimic mechanically soft ECM^36^. In contrast to cells on stiff substrate, those cultured on the soft substrate for 14 days developed significant lipid droplets, as detected by BODIPY staining (Fig. 6b, c), indicating a shift toward adipogenic transdifferentiation. Further analysis of these cells revealed a marked upregulation of adipocyte-specific genes, including *Cebpa*, *Cebpb*, *Cebpd*, and *Pparg* (Fig. 6d). At the protein level, we also detected increased expression of the adipogenic transcription factor CEBPA and the adipocyte protein Perilipin 1 in cells cultured on soft substrates (Fig. 6e, f). Additionally, these cells cultured on the soft substrate exhibited functional adipocyte characteristics, including elevated adiponectin secretion (Fig. 6g) and the ability to undergo lipolysis in response to the β-adrenergic agonist isoproterenol (Fig. 6h). These results demonstrated that MEFs cultured on soft substrates for 14 days acquired functional adipocyte properties, further supporting the notion that mechanical stimuli play a pivotal role in adipogenic transdifferentiation.

YAP is a well-established co-transcription factor that mediates classical mechanoresponses. Its nuclear translocation and co-transcriptional activity are directly regulated by mechanotransduction: YAP accumulates in the nucleus under high mechanical stress and is exported to the cytoplasm under low stress^37^. In our system, nuclear export of YAP was observed in Ble treated cells (Fig. 6i, j). To investigate whether Ble-induced adipogenic fate transition depends on YAP activation, we generated stable cell lines overexpressing wide-type YAP (YAP-WT) or constitutive activation YAP mutant (YAP-5SA) via lentiviral transduction. In YAP-5SA mutant, the phosphorylation sites required for cytoplasmic retention are mutated to alanine, thus preventing nuclear export^38^. Our results showed that overexpression of YAP-5SA, but not YAP-WT, effectively inhibited Ble-induced nuclear export of YAP and blocked adipogenic conversion, as assessed by Perilipin 1 staining (Fig. 6k, l). These results demonstrate that Ble-induced adipogenic conversion depends on YAP nuclear export, highlighting its role as a key mechanosensitive regulator and underscoring the importance of mechanical signaling in cell fate decisions.

## Discussion

Here, we report a strategy for regulating tumor cell fate by modulating mechanical signaling as a trigger, which successfully converting ovarian cancer cells into harmless adipocytes when combined with adipogenic cues. In ovarian cancer, metastatic tumor cells disseminate within the peritoneal cavity and frequently metastasize to adipose-rich peritoneal tissues^23,39^. This unique tropism provides a potential microenvironmental advantage for adipogenic transdifferentiation. By leveraging this feature in combination with the pharmacological modulation of the cytoskeleton, we successfully reprogrammed ovarian cancer cells. Building on previous studies demonstrating the transdifferentiation of breast cancer cells into adipocytes through classical signaling pathways^40^, our findings further validate the therapeutic potential of this approach. Notably, our strategy not only eliminates the requirement for cellular plasticity but also achieves a significantly higher adipogenic transdifferentiation efficiency (~70%) in a mouse model of peritoneal metastatic ovarian cancer. Moreover, we propose that optimizing the adipogenesis-favoring microenvironment through supplementation with exogenous cytokines or small molecules may further improve conversion efficiency. This approach exhibits considerable therapeutic potential for preventing tumor recurrence caused by residual tumor cells after surgical resection or immunotherapy-escaping cells.

Current limitations of ovarian cancer treatment include the following: R0 surgical resection leaves residual micro-metastases that can cause recurrence; platinum-based neoadjuvant chemotherapy shows high initial response rates but frequent subsequent drug resistance; targeted therapies are only effective for specific molecular subtypes, limiting their clinical application; and ovarian cancer is a “cold tumor” with an immunosuppressive microenvironment that contains abundant immunosuppressive cells that hinder immune cell attack^41^. In contrast, strategies that regulate cell fate target the fundamental survival mechanisms of tumor cells and can complement the above treatment approaches. Moreover, converting tumor cell fate does not depend on the immune microenvironment, making it effective even for cold tumors, such as ovarian cancer.

Transcription factors and epigenetic modifications are among the most extensively studied cell fate regulators. Previous studies have employed methods such as gene editing, epigenetic regulation, and cell reprogramming to alter tumor cell fate. For example, targeting the E7 oncogene using CRISPR/Cas9 can induce apoptosis in cervical cancer cells, leading to tumor eradication^42^. By modulating DNA methylation to alter the epigenetic state of cancer cells, site-specific methylation of the tumor suppressor Maspin and oncogene SOX2 in breast cancer cells can be achieved, thereby suppressing tumor growth^43^. Alternatively, overexpression of the neural transcription factor NeuroD1 can reprogram glioblastoma cells into noncancerous neurons, thereby inhibiting tumor progression^9^. However, these methods have certain limitations: gene editing may cause off-target effects, leading to unintended genomic damage^44,45^, the long-term stability and specificity of epigenetic regulation are difficult to ensure, related treatments may exert nonspecific effects on normal cells^46^, and cell reprogramming may be limited by challenges such as low efficiency and incomplete reprogramming^47,48^.

Herein, we propose the manipulation of tumor cell fate through mechanical force regulation, which represents a gene-editing-free strategy for cellular reprogramming. First, we pharmacologically disrupted the cytoskeleton to induce heterochromatin redistribution, increase chromatin accessibility, and promote binding of pioneer adipogenic transcription factors, thereby increasing the expression of adipogenic genes (Supplementary Fig. 7). Moreover, this process depends on YAP nuclear export. We subsequently validated the “cytoskeletal-microenvironmental regulation of cell fate” hypothesis via mechanical modulation. In the cellular mechanical regulation system, cells can sense and respond to extracellular mechanical signals by adjusting their cytoskeleton, such as increasing stress or tension, to match the increasing matrix stiffness^49^. Therefore, when we cultured cells on soft substrates to simulate the reduced cellular tension caused by Ble-induced cytoskeleton disruption and exposed them to adipogenic conditions, adipogenic transdifferentiation was induced. These findings fully demonstrate the crucial role of mechanical signals in regulating tumor cell fate and suggest that in addition to transcription factors and epigenetic modifications, mechanical regulation may represent a third dimension for maintaining or converting cell fate.

Although we have validated the anti-tumor efficacy of myosin II inhibition-mediated adipogenic transdifferentiation in subcutaneous and intraperitoneal ovarian cancer xenograft models, several limitations should be noted. Our study was performed in a limited set of ovarian cancer cell lines and conventional xenograft models, which cannot fully recapitulate the heterogeneity and complex microenvironment of primary human ovarian tumors, restricting the generalizability of our findings. In addition, bulk RNA-seq provides only population-level insights and cannot resolve tumor cell subpopulation dynamics or cellular interactions within the tumor microenvironment; future single-cell RNA sequencing will be required to dissect the heterogeneous mechanisms underlying adipogenic transdifferentiation. Consistent with this cellular and microenvironmental heterogeneity, the adipogenic conversion response showed clear spatial variation in vivo, with mature lipid droplets mainly observed at the tumor periphery or in small nodules, likely due to uneven intratumoral drug distribution that limits uniform transdifferentiation efficiency. Furthermore, the reduced tumor burden in our peritoneal metastasis model may reflect a combined effect of adipogenic transdifferentiation and suppressed tumor invasion and colonization, since myosin II inhibition also impairs cell migration and adhesion^50,51^, and the relative contribution of each process to the overall therapeutic effect remains to be further clarified. Of note, while the adipocyte-like cells generated from tumor cell transdifferentiation exhibited a stable, quiescent phenotype without evidence of pathological characteristics associated with cancer-associated adipocytes (CAAs), their long-term in vivo fate and potential to gradually acquire CAA-related tumor-promoting properties require further long-term observation and characterization; additionally, the long-term stability of this transdifferentiation strategy has not been fully evaluated, and whether the adipocyte phenotype can be stably maintained or may undergo phenotypic reversal still requires further validation using lineage-tracing approaches.

From the perspective of clinical translation, several additional considerations and limitations exist. The poor solubility and suboptimal pharmacological properties of Blebbistatin limit its direct clinical application, supporting the need to identify more drug-like myosin II inhibitors with improved physicochemical properties in future studies, such as MT228, an optimized non-muscle myosin II (NMII) inhibitor with superior biosafety and improved physicochemical properties^52^. Given the issue of uneven drug distribution noted earlier, our therapeutic strategy is particularly suitable for patients with minimal residual disease (MRD), in whom lower tumor burden facilitates more uniform drug penetration, whereas patients with large residual lesions may show reduced therapeutic efficacy due to insufficient drug infiltration. To overcome these challenges, optimizing administration routes, dosage, and frequency will be critical to improve treatment uniformity and translational potential.

In summary, our findings demonstrate that cytoskeleton disruption promotes adipogenic transdifferentiation in EOC via chromatin reorganization. By coupling cytoskeletal modulation with microenvironmental cues, this approach successfully converts tumor cells within the adipose-abundant peritoneal cavity into adipocytes, bypassing therapeutic resistance and immunosuppressive barriers. Notably, this strategy represents a gene-editing-free approach to tumor cell reprogramming, identifying mechanical regulation as a third dimension for cell fate control. Thus, it represents a promising malignant cell reprogramming strategy, providing a preclinical experimental basis for the development of targeted differentiation therapeutic strategies against ovarian cancer.

## Methods

### Cell lines and cell culture

Mouse embryonic fibroblasts (MEFs) were established from CF-1 mice and cultured in complete growth medium consisting of Dulbecco’s modified Eagle’s medium (DMEM; Gibco, 11995-065, USA) supplemented with 10% fetal bovine serum (FBS; Gibco, 10099141, USA) and 1× penicillin/streptomycin (Gibco, 15140163, USA) at 37 °C in a 5% CO_2_ atmosphere. Human ovarian cancer cell lines A2780 (purchased from Shanghai Zhongqiao Xinzhou Biotechnology Co., Ltd.), SKOV3 (purchased from ATCC, Lot. 70025324) and OVCAR3 (purchased from ATCC, Lot. HTB-161) were regularly authenticated via short tandem repeat typing (STR) and mycoplasma testing, and the cell lines were passaged in the same complete growth medium as described above.

### In vitro adipogenesis

Twelve-well plates (with or without coverslips) were coated with 20 μg/mL fibronectin (MCE, HYP3160, USA) for 30 min. MEFs, A2780, SKOV3 and OVCAR3 cells were seeded onto fibronectin-coated plates and cultured in complete growth medium for 24 h. Upon reaching ~30–50% confluence, the cells were switched to adipocyte induction medium supplemented with 20 μM (‒)-Blebbistatin (Ble; MCE, HY-13441, USA). The medium was changed every other day, and analysis of these cells was performed on D14.

The adipocyte induction medium used was the N2B27 medium containing 10% KnockOut™ Serum Replacement (Gibco, 10828028, USA), 20 ng/mL BMP4 (Novoprotein, CR93, China) or 2 μM Rosiglitazone (Rosi; MCE, HY-17386, USA). N2B27 medium was prepared by mixing DMEM/F12 (Gibco, 11320-033, USA) and neurobasal medium (Gibco, 21103-049, USA) in a 1:1 ratio, followed by supplementation with 0.5% N-2 supplement (Gibco, 17502048, USA), 1% B-27 supplement (Gibco, 17504044, USA), 10 μg/mL insulin (Pricella, PB180432, China), β-mercaptoethanol (1000×, Gibco, 21985023, USA), 0.02% bovine serum albumin (BSA; Sigma, A3803, USA), GlutaMAX (100×, Gibco, 10565-018, USA), and penicillin/streptomycin.

### Cell culture on soft substrate

Cytosoft plates with an elastic modulus of 64 kPa (Sigma, 5145, USA) were used to culture the cells on soft substrates. The plates were then coated with 40 μg/mL fibronectin for 1 h at room temperature. After incubation, any remaining solution was aspirated, and the coated surfaces were rinsed twice with Phosphate-Buffered Saline (PBS). After the second rinse, sufficient medium was left on the plates to ensure the complete coverage of the polydimethylsiloxane (PDMS) surface. The coated plates were considered ready for cell seeding.

### BODIPY staining and imaging

The cells were incubated with 1 μM BODIPY™ FL C12 (Invitrogen, D3822, USA) in the dark at room temperature for 15 min and washed twice with PBS. Images were acquired using an IX73 inverted microscope (Olympus, Tokyo, Japan). Representative images were analyzed using ImageJ 1.54 g software.

### RNA extraction and qRT-PCR

Total RNA was isolated from adherent cells or homogenized tumor tissues using a Total RNA Column Extraction Kit (Life-iLab, AN51L518, China) according to the manufacturer’s instructions. Briefly, cells were rinsed with PBS before lysis, and tissues were fully homogenized in advance. All samples were lysed with Lysis Buffer, mixed with an equal volume of absolute ethanol, loaded onto RNA spin columns, washed with Wash Buffer, and subjected to empty-tube centrifugation to remove residual buffer prior to elution with Elution Buffer. RNA was subsequently reverse-transcribed into cDNA using NovoScriptPlus All-in-one 1st Strand cDNASynthesis SuperMix (gDNA Purge) (Novoprotein, E047, China). The 20 μL reverse transcription system contained 0.1 ng–1 μg of total RNA, 2× reaction SuperMix and gDNA Purge for synchronous genomic DNA elimination. The reaction was incubated at 50 °C, followed by a 5 s incubation at 85 °C to inactivate reverse transcriptase and RNase inhibitor. qRT-PCR was performed in triplicate using NovoStartSYBR QPCR SuperMix Plus (Novoprotein, E096, China) on a QuantStudio 5 Real-Time PCR instrument (Applied Biosystems, USA). Each 20 μL qPCR reaction system consisted of 2× SYBR SuperMix, forward and reverse primers (final concentration: 0.6 μM), cDNA template and ROX reference dye. A two-step amplification protocol was applied: initial denaturation at 95 °C for 1 min, then 45 cycles of 95 °C for 20 s and 60 °C for 1 min. *Gapdh/GAPDH* served as the internal control. All the primers used are listed in Supplementary Tables 1, 2.

### RNA sequencing analysis

Total RNA was isolated from the cells and tumors in two independent experiments using a Total RNA Column Extraction Kit. RNA-seq libraries were prepared using the NEBNext® UltraTM RNA Library Prep Kit for Illumina® (NEB, E7530L, USA). Sequencing was performed on a HiSeq X-Ten sequencer (Illumina, USA) with a 150 bp paired-end sequencing reaction. All RNA-sequencing data analyses were performed with STAR (version 2.7.11a) and StringTie (version v2.1.7) using the UCSC hg38 annotation with default settings. Genes with a sum of fragments per kilobase of exon model per million fragments mapped (FPKM) values greater than 1 across all samples were removed and not used for downstream analysis. A twofold change in the FPKM value between the two groups and a *P* value less than 0.05 were used as the criteria for selecting differentially expressed genes (DEGs). Gene Ontology (GO) analysis was performed using KOBAS. Principal component analysis (PCA), heatmaps, and functional enrichment bar plots were generated using prcomp, pheatmap, and ggplot2 functions in R. Annotations for human transcription factors (TFs) were downloaded from TRRUST (version 2). The interaction network of the differentially expressed TFs was constructed using the STRING online tool STRING: functional protein association networks (https://string-db.org/) using Cytoscape. The Gene Set Enrichment Analysis was performed using the “GSEA” function and visualized through the “gseaplot2” function in the R package clusterProfiler.

### Immunofluorescence microscopy analysis of cells

Cells grown on coverslips were fixed with 4% paraformaldehyde (PFA) for 10 min at room temperature, followed by permeabilization with 0.3% Triton X-100 (Sigma, T8787, USA). After blocking with 2% BSA for 1 h, the samples were incubated with primary antibodies at the following dilutions: anti-CEBPA (CST, 8178S, USA; 1:100), anti-Perilipin 1 (CST, 9349S, USA; 1:200), anti-Adiponectin (Proteintech, 21613-1-AP, China; 1:200), anti-Leptin (Proteintech, 17436-1-AP, China; 1:200), anti-Ki67 (Abcam, ab15580, UK; 1:1000), and anti-Human Nucleoli [NM95] (Abcam, ab190710, UK; 1:100) for 2 h at room temperature or overnight at 4 °C. After washing, the cells were incubated with the secondary antibodies: Donkey Anti-Rabbit IgG H&L (Alexa Fluor® 488) (Abcam, ab150073, UK; 1:400), Donkey Anti-Mouse IgG H&L (Alexa Fluor® 594) (Abcam, ab150108, UK; 1:400), and Donkey Anti-Rabbit IgG H&L (Alexa Fluor® 647) (Abcam, ab150075, UK; 1:400) for 1 h at room temperature. After the final washing step, the coverslips were mounted with SlowFade Glass Antifade Mountant with DAPI (Invitrogen, S36920, USA). Fluorescence imaging was performed using an Olympus IX73 inverted microscope or a Nikon laser confocal microscope AXR (Ti2-E) (Nikon, Japan). Quantitative analysis of CEBPA-, Perilipin 1-, Adiponectin-, Leptin-, and Ki67-positive cells was conducted by counting at least three random fields per replicate using the ImageJ 1.54 g software.

### Adiponectin ELISA analysis

To quantify adiponectin secretion, cell culture supernatants from Ctrl and treated cells and cells subjected to the 14-day adipogenic transdifferentiation protocol were collected. For human adiponectin detection using the Human Adiponectin ELISA Kit (Abcam, ab99968, UK), all concentrated reagents were diluted to working concentrations prior to use. Samples and standards (100 μL per well) were incubated at room temperature for 2.5 h, followed by sequential incubation with biotinylated antibody and Horseradish Peroxidase (HRP)-streptavidin after repeated washing. After TMB chromogenic reaction and termination, absorbance was measured at 450 nm, and sample concentrations were calculated via a 4PL standard curve. For mouse adiponectin detection using the Mouse ADP/Acrp30 (Adiponectin) ELISA Kit (Elabscience, E-EL-M0002, China), concentrated buffers and antibodies were diluted to working solutions. Samples and standards (100 μL per well) were incubated at 37 °C for 90 min, then reacted with biotinylated antibody and HRP conjugate with specified washing cycles. Color development was performed with TMB substrate at 37 °C, and absorbance was detected at 450 nm after reaction termination. Concentrations were determined using a 4PL standard curve.

### Lipolysis analysis

To induce lipolysis, Ctrl and treated cells and cells subjected to the 14-day adipogenic transdifferentiation protocol were treated with 100 nM isoproterenol. All cells were cultured and assayed in 96-well flat-bottom plates. Prior to stimulation, differentiated cells were gently washed twice with 100 μL lipolysis wash buffer per well, and then incubated in 150 μL lipolysis assay buffer. After 3 h of lipolysis stimulation, 50 μL of cell culture supernatant was collected. A glycerol standard curve ranging from 0 to 10 nmol per well was prepared in the same 96-well plate via serial dilution of glycerol standard. For colorimetric detection, a 50 μL reaction mix containing 46 μL assay buffer, 2 μL enzyme mix and 2 μL OxiRed probe was added to each well. The plate was incubated at room temperature for 30 minutes in the dark, and absorbance was measured at 570 nm using a microplate reader. Glycerol concentrations were calculated by referencing the standard curve after blank value subtraction. Glycerol release was quantified using a Lipolysis Assay Kit (Colorimetric) (Abcam, ab185433, UK).

### Cell Counting Kit-8 (CCK-8) assay

The cells were seeded in 96-well plates at a density of 3 × 10^3^ cells/well and cultured at 37 °C with 5% CO_2_. After cell attachment, the medium was replaced with a fresh culture medium. At 24, 48, 72, and 96 h post-seeding, 20 μL of CCK-8 reagent (Apexbio, K1018, USA) was added to each well, and after 2 h of incubation, absorbance was measured at 450 nm using an Infinite F50 Absorbance Reader (Tecan, Switzerland).

### EdU incorporation to detect proliferating cells

The EdU incorporation assay for detecting proliferating cells was performed in 4-well plates using the E-Click EdU Cell Proliferation Imaging Assay Kit (Green, Elab Fluor 488) (Elabscience, E-CK-A376, China) according to the manufacturer’s instructions. Briefly, Ctrl-treated cells and cells subjected to the adipogenic transdifferentiation protocol for 14 days were labeled with 10 μM EdU for 2.5 h at 37 °C, fixed with 4% PFA for 15 min, washed with PBS, permeabilized with 0.3% Triton X-100, and blocked with 3% BSA in PBS. The Click reaction was incubated at room temperature for 30 min in the dark, followed by DAPI nuclear counterstaining. Fluorescence images were acquired using an Olympus IX73 inverted microscope, and at least three random fields were quantified using ImageJ 1.54 g software.

### DNase I-TUNEL assay

DNase I-TUNEL assays were performed as previously described^53^ with some modifications. Briefly, cover slip cultures were fixed with 4% PFA for 15 min at room temperature and permeabilized with 0.3% Triton X-100. The cells were then incubated with graded concentrations (0.1, 0.01, and 0.001 U/μL) of DNase I (NEB, M0303S, USA) for 5 min at 37 °C in DNase I reaction buffer. The TUNEL assay was subsequently performed using a One-step TUNEL FITC Apoptosis Detection Kit (Apexbio, K1133, USA). After equilibration with diluted equilibration buffer, samples were incubated with prepared TUNEL labeling solution at 37 °C for 60 min under dark conditions, followed by repeated PBS washing and DAPI nuclear counterstaining. Fluorescence images were acquired using a Nikon laser confocal microscope AXR (Ti2-E), and quantification was performed using Nis Elements Imaging Software (version 5.21.00).

### Chromatin immunoprecipitation–quantitative RT-PCR (ChIP-qPCR)

ChIP–qPCR assays were performed using the SimpleChIP® Plus Enzymatic Chromatin IP Kit (Cell Signaling Technology, 9005S, USA). Cells were cross-linked with 1.5% formaldehyde, and chromatin was fragmented via micrococcal nuclease digestion combined with sonication to obtain DNA fragments ranging from 200 to 500 bp. Fragmented lysates were incubated with specific primary antibodies together with Protein G magnetic beads at 4 °C with continuous rotation overnight. The following antibodies were used for immunoprecipitation: anti-HP1α (CST, 2616S, USA; 1:25), anti-H3K9me3 (Abcam, ab8898, UK; 2 µg per 25 µg chromatin), anti-H3K27ac (Abcam, ab177178, UK; 2 µg per 25 µg chromatin), and anti-RNA Pol II (Abcam, ab5408, UK; 1:500). After multiple low-salt and high-salt washing steps, captured DNA was eluted, de-crosslinked at 65 °C and purified. 10% of the total chromatin was collected as the input control throughout the experiment. The purified DNA was analyzed by qPCR. qPCR was performed with the following program: initial denaturation at 95 °C for 1 min, followed by 45 cycles of 95 °C for 20 s and 60 °C for 1 min. The primer sequences used in this study are listed in Supplementary Table 3.

### Western blot analysis

Western blotting was performed using standard protocols. Primary antibodies against CEBPA (CST, 8178S, USA), Perilipin 1 (CST, 9349S, USA), and β-actin (Sigma, A1978, Germany) were used at dilutions of 1:1000 (for CEBPA and Perilipin 1) and 1:5000 (for β-actin). Protein samples were separated by SDS-PAGE and transferred to PVDF membranes prior to antibody incubation. After incubation with IRDye 800CW goat anti-rabbit IgG (H + L) secondary antibody (LI-COR, 926-32211, USA, 1:10000), the protein bands were detected using an Azure chemiluminescence imaging system (Azure, USA).

### Mice

Specific pathogen-free (SPF)-grade female BALB/c nude mice aged 4–6 weeks and weighing 14–16 g were purchased from the Vital River Laboratory Animal Technology Co., Ltd. (Beijing, China; production license SYXK (Ji) 2023-0011). Mice were housed under a 12-h light/12-h dark cycle at 22 ± 2 °C and 40–60% humidity in individually ventilated cages (IVCs) with aspen and corncob bedding, with free access to standard laboratory chow and water. No environmental enrichment was provided. All animal experiments were conducted at the Experimental Animal Center of the First Hospital of Jilin University. The study protocol was approved by the Animal Ethics Committee of the First Hospital of Jilin University (Approval No. JDYY20250740). We have complied with all relevant ethical regulations for animal use. All housing conditions and experimental procedures strictly complied with the Guidelines for the Management of Laboratory Animals. Humane endpoints were defined as tumor volume exceeding 2 cm³, body weight loss of 20–25%, or the development of cachexia or a moribund state; animals were monitored daily. For live imaging, mice were anaesthetized with tribromoethanol (250 mg/kg, i.p.). At the end of the experiments, mice were euthanized by gradual displacement of carbon dioxide (CO₂) in a closed chamber using a compressed gas cylinder, with a flow rate achieving 30–50% chamber volume displacement per minute. Animals were left in the chamber for at least 5 min after respiratory arrest, and death was confirmed by cervical dislocation prior to tissue collection.

### Establishment of a subcutaneous xenograft model and treatment

To establish a subcutaneous xenograft model of ovarian tumors, A2780 cells (5 × 10^6^ cells) suspended in a 1:1 mixture of fresh culture medium and Matrigel (354248, BD Biocoat, USA) were injected subcutaneously into the scapula region of 6-week-old female BALB/c nude mice. One day after injection, the mice were treated daily with intratumoral and paratumoral injections of 20 μL of drug or vehicle for two weeks. After the treatment period, the mice were sacrificed and the tumor volume and weight were measured. During the experimental period, tumor growth was closely monitored and recorded to ensure compliance with the ethical limit (≤2 mm³) set by the Animal Ethics Committee. Upon excision, the tumors were processed for further analysis and embedded in paraffin for immunofluorescence staining or in OCT compound for Oil Red O staining. The treatment groups were as follows: the Ble + BMP4 group (3 mg/kg Ble dissolved in vehicle and 10 μg/kg BMP4 dissolved in PBS containing trehalose), the Ble + Rosi group (3 mg/kg Ble and 20 mg/kg Rosi dissolved in vehicle), and the Ctrl group [vehicle alone (2% DMSO, 40% poly(ethylene glycol) 400 (Coolaber, CP8187, China), 2% TWEEN^®^ 80 (Aladdin, T104866, USA), 56% saline solution)].

### Establishment of an intraperitoneal ovarian cancer metastasis model and treatment

To establish an intraperitoneal ovarian cancer metastasis model, 6-week-old female BALB/c nude mice were intraperitoneally injected with SKOV3 or OVCAR3 cells (1 × 10^7^ cells suspended in 500 μL PBS). One day post-injection, the mice received daily intraperitoneal (i.p.) injections of 200 μL of drug or vehicle for 10 days. After the treatment period, the mice were sacrificed, and the tumors were embedded in paraffin for immunofluorescence staining. Tumor progression was monitored every 5 days using live animal bioluminescence imaging to assess the metastatic tumor burden. The treatment groups were as follows: Ble group (3 mg/kg Ble dissolved in vehicle) and Ctrl group [vehicle alone (2% DMSO, 40% PEG400, 2% TWEEN® 80, 56% saline solution)].

### Oil Red O staining of tissue

Lipid accumulation in tumors was confirmed using a modified Oil Red O staining kit (Solarbio, G1261, China) according to the manufacturer’s instructions. Briefly, the tumors were snap-frozen in Tissue-Tek OCT compound (SAKURA, 4583, USA), and 7 μm thick tumor sections were cut and rinsed with distilled water and then soaked in 60% isopropanol for 20–30 s. The tumors were stained with a modified Oil Red O Stain Solution for 10–15 min. After staining, the slices were washed with 60% isopropanol to remove the dye solution, and then rinsed with distilled water. The samples were re-stained with Mayer’s hematoxylin solution for 1–2 min. The sections were then rinsed with distilled water. The water around the slices was blotted with filter paper, and the sections were sealed with glycerin gelatin. Oil Red O-stained tumor slices were imaged using an Olympus IX73 inverted microscope.

### Immunofluorescence microscopy analysis of tumors

Tumor samples were fixed in 4% PFA for 2 h, dehydrated through a graded ethanol series, and embedded in paraffin. Sections (4–5 μm thick) were cut, deparaffinized (xylene followed by ethanol gradients), and subjected to heat-induced antigen retrieval using Tris-EDTA buffer (95 °C). After blocking with 5% BSA, the sections were incubated with the primary antibody (Supplementary Table 4) at 4 °C overnight, followed by incubation with fluorescent secondary antibodies at room temperature for 1 h. The nuclei were counterstained with DAPI and the slides were mounted with an anti-fade mounting medium. Fluorescence imaging was performed using either a VS200 Research Slide Scanner (Olympus, Japan) or a Nikon AXR laser confocal microscope (Ti2-E). For quantitative analysis, the area containing cells positive for both HuNu and Perilipin 1 was quantified in at least three random fields per replicate using the ImageJ 1.54 g software.

### Intravital imaging

For intravital imaging, tumor-bearing mice were anesthetized *via* intraperitoneal injection of tribromoethanol (250 mg/kg). Prior to imaging, 0.2 mL of D-luciferin potassium salt (15 mg/mL in PBS; GoldBio, LUCK-1G, USA) was administered intraperitoneally. Bioluminescence imaging was performed 10 min post-injection using an IVIS Lumina XR imaging system (PerkinElmer, USA) with the following parameters: exposure time, 1 s–1 min (auto-adjusted); field of view = 7.5 cm. Image acquisition and quantification were performed using Living Image software (v4.7.2, PerkinElmer) with standardized regions of interest (ROIs). All procedures were approved by the Animal Ethics Committee of the First Hospital of Jilin University.

### Statistics and reproducibility

All experiments were performed in at least three independent biological replicates unless otherwise specified. Biological replicates for cell-based assays were defined as independently cultured cell batches; for animal studies, each individual animal was considered a replicate. For quantitative data, results are shown as mean ± standard error of the mean (SEM). For qPCR, each sample was run in three technical replicates; for all other assays (including ELISA, lipolysis, and CCK8), each biological replicate contributed a single measurement, as preliminary tests confirmed negligible intra-assay variability. Sample sizes (*n*) indicate the number of independent biological replicates; exact *n* values are given in figure legends. For image acquisition and analysis, random visual fields were selected from each group to avoid artificial selection bias. All images were captured under identical microscopic parameters, and a unified analysis threshold was applied for positive cell calculation. Data are presented as mean ± SEM. Group comparisons were performed using two-sided Student’s *t* tests (paired or unpaired as appropriate) or one-way analysis of variance (ANOVA) with Tukey’s test (or Welch’s ANOVA with Dunnett’s T3 when variances were unequal). Multiple comparisons across multiple genes or panels were corrected using the two-stage step-up method (Benjamini, Krieger and Yekutieli; False Discovery Rate (FDR) = 5%). GraphPad Prism 9.5.0 was used. All *P* values indicating significance are shown in the figures: *P < 0.05, **P < 0.01, ***P < 0.001, ****P < 0.0001, n.s. *P* ≥ 0.05.

### Ethics approval and consent to participate

The study protocol was approved by the Animal Ethics Committee of the First Hospital of Jilin University (Approval No. JDYY20250740). We have complied with all relevant ethical regulations for animal use. All housing conditions and experimental procedures strictly complied with the Guidelines for the Management of Laboratory Animals.

### Reporting summary

Further information on research design is available in the Nature Portfolio Reporting Summary linked to this article.

## Supplementary information

Transparent Peer Review file

Supplementary Information

Description of Additional Supplementary Files

Supplementary Data 1

Reporting Summary

## Data Availability

RNA-seq data of MEFs have been deposited in the Genome Sequence Archive of the Beijing Institute of Genomics, Chinese Academy of Sciences (https://ngdc.cncb.ac.cn/). The accession number is CRA024556. RNA-seq data of A2780, SKOV3, and OVCAR3 cells (accession number: HRA018018) and A2780 subcutaneous tumors (accession number is HRA011043) have been deposited in the Genome Sequence Archive for human (GSA-Human) of the Beijing Institute of Genomics, Chinese Academy of Sciences (https://ngdc.cncb.ac.cn/gsa-human/). Uncropped Western blot images are provided in Supplementary Fig. 8. The numerical source data for all plots and summary statistics are provided in Supplementary Data 1.xlsx. The mouse adipose tissue RNA-seq dataset used in Fig. 1g is available from the NCBI Sequence Read Archive (accession ERX4742183) and is cited in the reference list.
