# Supplementary material for: Mechanical regulation of adipogenic reprogramming suppresses ovarian cancer progression
Source: Commun Biol. 2026 Jun 29;9:1506. doi: 10.1038/s42003-026-10567-4 (PMC13752288; doi:10.1038/s42003-026-10567-4)
Supplement: Supplementary file 1 [file 42003_2026_10567_MOESM1_ESM.pdf]

nature portfolio

Peer Review File

Mechanical regulation of adipogenic reprogramming suppresses ovarian cancer progression

Corresponding Author: Dr Zheng-Quan He

This file contains all reviewer reports in order by version, followed by all author rebuttals in order by version.

Attachments originally included by the reviewers as part of their assessment can be found at the end of this file.

Version 0:

Reviewer comments:

Reviewer #1

(Remarks to the Author)
Review of “Mechanical regulation of adipogenic reprogramming suppresses ovarian cancer progression” by Li et al.
Summary
Li et. al presented a study that investigated cytoskeletal regulation as a therapeutic strategy in epithelial ovarian cancer (EOC). The authors report that combining blebbistatin (Ble) with adipogenic cues (BMP4, PPARγ agonists) induces adipocyte-like transdifferentiation of ovarian cancer cells in vitro and in vivo, evidenced by lipid droplet accumulation, adipogenic marker expression, and β-adrenergic–responsive lipolysis. In mouse models, Ble treatment suppressed tumor growth, with HuNu/Perilipin-1 colocalization confirming tumor cell conversion. Mechanistically, cytoskeletal destabilization triggered heterochromatin remodeling, enabling CEBPα/CEBPβ activity. Adipogenic conversion also occurred on soft substrates and was blocked by constitutively nuclear YAP, highlighting mechanical signaling as a key regulator.
Overall impression:
The concept of mechanically induced reprogramming is relevant, and the authors apply a broad methodological toolkit of high quality. However, several claims, particularly regarding terminal differentiation and adipocyte morphology, are not convincingly supported by the data shown. The mere accumulation of neutral TAG in small lipid droplets stained by Bodipy and induction of LD-formation relevant genes does not indicate necessarily an adipocyte identity; true adipogenesis requires expression of adipokines such as ADIPOQ or LEPTIN, and the formation of large lipid droplets, which was not observed in Ble-treated cancer cells. Based on the current data, Ble-treated cells rather appear to undergo metabolic stress due to loss of actin–myosin structure, leading to ER perturbation and formation of small lipid droplets. The absence of large, fused lipid droplets typical of adipocytes suggests that lipid droplet maturation is impaired, and therefore the term “adipocytes” is not appropriate in this context.
Major points
1. Terminology & Clarity
o Line 112: “terminally differentiated adipocytes” is incorrect; please use “adipocyte-like” True terminal adipocytes cannot be recapitulated in vitro within 14 days. See also “Overall impression” above.
2. Lipid droplet accumulation vs. adipogenesis
o Data shown in Fig. S2 (ROSI treatment alone) showed stronger lipid accumulation in MEFs than Ble-treatment in terms of the number and size of Bodipy-stained cells, and the expression of PPARG/FABP4. Why do the authors stick to blebbistatin when ROSI has a stronger effect?
o Fig. 2A shows numerous small lipid droplets in blebbistatin-treated A2780 cells that do not exhibit in vitro adipocyte morphology or lipid droplet fusion, as observed in Fig. S2A in MEFs. The authors should explain why they believe LD accumulation indicates adipogenesis rather than being merely a consequence of increased FA synthesis to maintain survival under cytoskeletal stress. Lipid droplets: a cellular organelle vital in cancer cells | Cell Death Discovery.
o Also, PLIN1 staining appeared to be more cytoplasmic than LD-specific, with increased staining observed in almost all cells. At least, judging by the image presented. Does PLIN1 colocalise with LDs in Ble-treated A2780 cells?
o In this context RNA-seq data from “differentiated” A2780 cells as shown for MEFs in Fig. 1F shall be provided to support a “adipocyte”-like phenotype of Ble-treated A2780 cells.
o In Fig. 2c, the effect of Ble versus rosiglitazone (Rosi) treatment on peroxisome proliferator-activated receptor gamma (PPARG) and other differentiation markers was contrary to that observed in MEFs. What is the explanation for that?
o Although there is increased PLIN1 expression in vivo (see Figures 3 and 4), the cancer cell-derived cells do not exhibit

typical adipocyte morphology, characterised by large, fusing lipid droplets. The authors must explain this observation in the discussion section.
3. Gene expression analysis
o In Fig. 2d the authors showed that “ACTB” is downregulated in RNAseq data. This makes this gene unsuitable as a reference gene for quantitative real time PCR. Please re-analyze with validated stable reference genes (for example see Reliable reference genes for expression analysis of proliferating and adipogenically differentiating human adipose stromal cells - PubMed).
o Clarify what is meant by “relative expression of mRNA”, shown are fold changes vs. ctr of housekeeping genes normalized values?
o Indicate whether SE or SD are shown in the figure legends?
4. Cell line choice & Methodological clarification
o In figure 3, Ble was used always used in combination with BMP4 or ROSI but not administrated alone while in Figure 4 only Ble was used. Is there a reason for change in treatment strategy?
o In Figure 3, the author used A2780 (therapy-sensitive) for the subcutaneous xenograft model, while SKOV3 (therapy-resistant) was used for intraperitoneal injection. Firstly, the main text and figure legends do not state that different cell types have been used. The authors must consistently refer to the cell types used in the figure captions and the main text to avoid confusing the reader. Secondly, there is no in vitro data showing that SKOV3 cells can “transdifferentiate” in a manner similar to A2780 cells. The authors must provide evidence showing whether or not SKOV3 behave similarly to A2780 cells in response to Ble-treatment.
Minor points.
8. Fig. 1f: Specify the selection criteria for the “top 50 differentially expressed genes.”
9. Fig. 3b / Fig. S4a: provide statistical details (p-values; specify whether SE or SD is shown).
10. Fig. 3j: Lipid droplet morphology is not visible; please explain.
11. Oil Red O staining: clarify whether the peripheral staining in mouse tissue reflects tumor-associated lipid storage.
12. Line 210 / Fig. 4d: clarify whether Ble+BMP4-induced “transdifferentiation” is local, and explain what the perilipin+ areas in the vehicle controls represent.
13. Fig. 3h vs. Fig. 4d: clarify whether outcomes depend on tumor cell administration route or treatment condition.
14. Line 273: specify in the main text that ChIP-qPCR was used to demonstrate CEBPα/PPARγ binding to HP1/LMNA/C.
15. Line 306 / Fig. 6: specify which cell type was cultured on soft vs. stiff matrices (A2780 vs. MEFs). Clarify whether adipogenesis was induced solely by substrate stiffness or if additional cocktails were used.
16. Substrate stiffness: While it is true that 64 kPa (the Young's modulus of young cartilage) is soft relative to plastic (~GPa), it is much stiffer than adipose tissue (~0.5–2 kPa). What is the biological relevance of a 64 kPa 'soft' surface, and how does it relate to the adipogenic niche?
17. Overexpression system: Describe the system used to overexpress the YAP-5SA mutant. A control (e.g. YAP wt) is missing to confirm that the YAP-5SA mutant indeed negates the effects of the BLE on cells.

Reviewer #2

(Remarks to the Author)
Brief summary of the manuscript
Li et al. investigate the effect of pharmacological disruption of actomyosin contractility with (–)-Blebbistatin combined with adipogenic cues on ovarian cancer cells. They report that this approach induces adipogenic reprogramming both in vitro and in vivo, linking the observed conversion to chromatin remodeling and YAP nuclear export. The authors propose this as a novel therapeutic concept for ovarian cancer.

Overall impression of the work
The study is conceptually novel and timely, presenting a coherent, multi-layered dataset that integrates mechanistic and in vivo work. However, statistical underpowering, incomplete reporting, and several gaps in experimental validation and translational anchoring reduce confidence in the generalizability and durability of the effect. Addressing the experimental and reporting issues outlined below would substantially strengthen the manuscript.

Specific comments, with recommendations
Major comments
1. Statistical power and transparent reporting
Increase or describe in detail the biological n for key assays, including imaging quantifications (state how many cells and fields of view were analyzed per replicate), RT-qPCR, and in vivo readouts. In every figure legend, specify: exact statistical tests, two-sidedness, multiple-testing correction, alpha, and the definition of replicates. Use adjusted p-values for RNA-seq and targeted assays with multiple comparisons. Overlay individual data points on all plots and provide raw Ct tables (targets and endogenous controls) for RT-qPCR experiments.
2. RNA-seq pathway validation (GSEA)
Perform GSEA for curated adipogenic differentiation signatures and report NES/q (FDR) to validate pathway-level upregulation beyond DEG lists. Show representative adipogenesis-related transcription factors and downstream effector genes from the RNA-seq analysis.
3. MEF controls and prior literature
Reconcile the absence of adipogenesis in MEF controls (Fig. 1b,c) with the well-described multi-lineage potential of MEFs. Provide optimization data or rationale (medium composition, cell confluence, timing, substrate coating, insulin/BMP4 dosing) or include a positive-control protocol demonstrating expected adipogenesis under standard conditions. In addition, including a second cell type with known adipogenic potential—such as mesenchymal stem cells—would help establish whether

Blebbistatin generally facilitates adipogenesis.

4. Model breadth and clinical anchoring

Add at least one additional ovarian cancer cell line for all core in vitro and in vivo phenotypes. Ideally, extend to patient-derived organoids or primary tumor cells to increase clinical and translational relevance.

5. Single-cell resolution of reprogramming (optional but highly valuable)

Incorporate scRNA-seq and/or scATAC-seq to define the fraction of cells undergoing conversion, characterize heterogeneity, and directly link chromatin accessibility to adipogenic transcription factor activity. This would strengthen mechanistic insight and help anticipate tumor microenvironment influences, including the risk of cancer-associated adipocyte (CAA) emergence.

6. In vivo efficacy endpoints

Extend beyond short-term tumor volume measurements by including survival and recurrence analyses (e.g., Kaplan–Meier) and longer follow-up. This is critical, as novel therapies often show strong early effects that later diminish due to resistance mechanisms.

7. Safety/off-target profiling

Provide basic toxicology and off-target assessments for myosin II inhibition in non-tumor tissues (histopathology, serum chemistries, body weight) and discuss dosing/pharmacokinetic considerations, given Blebbistatin's known cytotoxicity, photoinstability, and poor solubility.

8. Durability of adipogenic conversion and CAA risk

Assess the stability of the induced adipogenic state after drug withdrawal (e.g., lineage tracing or washout experiments). Evaluate whether converted cells could become CAAs, which are known to support metastasis and therapy resistance, and discuss these implications for relapse risk.

Minor comments

1. Imaging and consistency

Provide higher-magnification BODIPY images to evaluate lipid droplet size and maturity. Improve resolution in Fig. 2 and ensure representative fields match the quantified percentages. State whether image selection and thresholding were blinded.

2. Heatmaps and units (Fig. 1f)

Replace "max/min" alone with a clear numeric scale (e.g., log2FC or z-score) and specify measurement units and data transformation.

3. Figure and legend specificity

For each panel, specify the assay platform (RNA-seq vs RT-qPCR), the exact cell type used (e.g., Fig. 6), and the number/type of replicates. Define all abbreviations and clarify what "max/min" denote.

4. qPCR data presentation (Figs. 2c, 3d; Suppl. Fig. 3SC)

Overlay individual data points; provide raw Ct tables; correct missing SD values (e.g., FABP4 in Suppl. Fig. 3SC). Investigate and explain unexpected zero SD in cancer-cell differentiation experiments.

5. Terminology and scope of conclusions

Calibrate claims in the Abstract and Discussion to reflect the current preclinical status. Explicitly acknowledge limitations regarding model scope, conversion durability, and safety.

If the authors increase statistical rigor and transparency, add pathway-level validation (GSEA), broaden and clinically anchor their model systems, consider single-cell resolution or at minimum strengthen conversion durability data, and expand in vivo endpoints and safety readouts, this work would make a meaningful contribution to the field.

Version 1:

Reviewer comments:

Reviewer #1

(Remarks to the Author)

The authors have thoroughly addressed all comments raised in the previous review and provided clear and well-reasoned responses to the points of criticism. The revisions have significantly improved the manuscript. In its current form, the manuscript is suitable for publication.

Reviewer #2

(Remarks to the Author)

The revised manuscript addresses a novel and potentially relevant concept: mechanical disruption of actomyosin contractility, together with adipogenic cues, may promote adipocyte-like reprogramming of ovarian cancer cells and reduce tumor progression. The authors have made a substantial effort to revise the work, including additional ovarian cancer cell lines, further in vivo validation, positive adipogenic controls, adipose-derived MSC experiments, and replacement of ACTB with GAPDH for qRT-PCR normalization. These additions improve the biological scope and are appreciated.

However, key statistical concerns remain insufficiently resolved. Many central conclusions rely on qRT-PCR panels with small replicate numbers, typically n = 3, and multiple genes tested in parallel. Several control qPCR groups still appear fixed at exactly 1 without visible SD/SEM. If these controls were artificially set as reference values rather than plotted as replicate-level normalized data, the biological variance of the control group is not displayed and the validity of the statistical tests

becomes difficult to assess. The authors should replot qRT-PCR data to show replicate-level variability in both control and treated groups, provide raw Ct/ΔCt values, and clearly document how statistics were performed.

The use of multiple unpaired t-tests across multi-gene qPCR and ChIP-qPCR panels also remains problematic unless appropriate multiple-testing correction is applied and reported. In addition, the RNA-seq/GSEA results should be interpreted more cautiously, as some reported adipogenic enrichments are borderline or not statistically significant, while the manuscript still uses strong wording.

The response regarding MEF adipogenic differentiation is valuable. The added positive-control adipogenic induction kit and adipose-derived MSC data strengthen the experimental framework. However, these data mainly support that Blebbistatin can facilitate adipogenic conversion under adipogenic conditions; they do not fully establish that mechanical disruption alone is instructive.

Overall, the revision improves the biological narrative, but the statistical and quantitative reporting still do not reach a fully satisfactory level. A focused revision addressing qPCR variance display, raw Ct/ΔCt transparency, multiple-testing correction, and more cautious interpretation of pathway analyses is required before the conclusions can be considered sufficiently supported.

Version 2:

Reviewer comments:

Reviewer #2

(Remarks to the Author)
The authors have adequately addressed the remaining concerns raised during the previous review round. In particular, the qRT-PCR data presentation has been substantially improved by replacing artificially normalized relative expression values with ΔCt-based reporting, including replicate-level variability, raw Ct/ΔCt transparency, and multiple-testing correction.

The authors have also revised the interpretation of RNA-seq/GSEA results more cautiously and clarified that Blebbistatin acts as a permissive mechanical sensitizer rather than an independent instructive differentiation signal. These revisions improve the statistical transparency and biological precision of the manuscript.

Although some datasets remain limited by small replicate numbers, the revised presentation is now sufficiently transparent, and the conclusions are supported by multiple complementary experimental approaches. I therefore consider the manuscript suitable for publication in its revised form.

Open Access This Peer Review File is licensed under a Creative Commons Attribution 4.0 International License, which permits use, sharing, adaptation, distribution and reproduction in any medium or format, as long as you give appropriate credit to the original author(s) and the source, provide a link to the Creative Commons license, and indicate if changes were made.
In cases where reviewers are anonymous, credit should be given to 'Anonymous Referee' and the source.

The images or other third party material in this Peer Review File are included in the article's Creative Commons license, unless indicated otherwise in a credit line to the material. If material is not included in the article's Creative Commons license and your intended use is not permitted by statutory regulation or exceeds the permitted use, you will need to obtain permission directly from the copyright holder.
To view a copy of this license, visit https://creativecommons.org/licenses/by/4.0/

Response to Reviewers' Comments

Dear Editors and Reviewers:

Thank you for reading and reviewing our manuscript. We appreciate the insightful comments and thoughtful suggestions provided by the reviewers and editors to help us improve the manuscript significantly. In response to these comments, we have carefully revised the manuscript and conducted additional experiments as recommended. All revisions and newly added content in the tracked revised version of the manuscript are highlighted in *red*.

Below are our point-by-point responses to the reviewers' comments.

Reviewer #1 (Remarks to the Author):

Summary

Li et. al presented a study that investigated cytoskeletal regulation as a therapeutic strategy in epithelial ovarian cancer (EOC). The authors report that combining blebbistatin (Ble) with adipogenic cues (BMP4, PPARγ agonists) induces adipocyte-like transdifferentiation of ovarian cancer cells in vitro and in vivo, evidenced by lipid droplet accumulation, adipogenic marker expression, and β-adrenergic–responsive lipolysis. In mouse models, Ble treatment suppressed tumor growth, with HuNu/Perilipin-1 colocalization confirming tumor cell conversion. Mechanistically, cytoskeletal destabilization triggered heterochromatin remodeling, enabling CEBPα/CEBPβ activity. Adipogenic conversion also occurred on soft substrates and was blocked by constitutively nuclear YAP, highlighting mechanical signaling as a key regulator.

Overall impression:

The concept of mechanically induced reprogramming is relevant, and the authors apply a broad methodological toolkit of high quality. However, several claims, particularly regarding terminal differentiation and adipocyte morphology, are not convincingly supported by the data shown. The mere accumulation of neutral TAG in small lipid droplets stained by Bodipy and induction of LD-formation relevant genes does not indicate necessarily an adipocyte identity; true adipogenesis requires expression of adipokines such as ADIPOQ or LEPTIN, and the formation of large lipid

droplets, which was not observed in Ble-treated cancer cells. Based on the current data, Ble-treated cells rather appear to undergo metabolic stress due to loss of actin－myosin structure, leading to ER perturbation and formation of small lipid droplets. The absence of large, fused lipid droplets typical of adipocytes suggests that lipid droplet maturation is impaired, and therefore the term "adipocytes" is not appropriate in this context.

Major points

1. Terminology & Clarity

Line 112: "terminally differentiated adipocytes" is incorrect; please use "adipocyte-like" True terminal adipocytes cannot be recapitulated in vitro within 14 days. See also "Overall impression" above.

Response:

We sincerely appreciate your valuable comment, which has greatly helped us improve the terminological accuracy and scientific validity of our manuscript.

To rigorously validate the adipogenic identity of the induced cells and refine our characterization of cell fate, we performed high-resolution imaging of lipid droplets in Ble+BMP4-treated A2780 cells, which confirmed the presence of enlarged lipid droplets consistent with adipogenic maturation **(Revised Fig. S3a)**. Furthermore, we examined the expression of key adipokines: ADIPOQ—a highly specific marker of mature adipocytes (PMID: 8631877; PMID: 7592907)—and LEPTIN—a hallmark of functional adipocyte differentiation (PMID: 9674695; PMID: 21952300). Both were significantly upregulated at the mRNA and protein levels. The detection of markedly increased ADIPOQ and LEPTIN expression, together with the upregulation of the fat-specific transcription factor CEBPs and PPARg **(Revised Fig. S3c, d)**, clearly distinguishes genuine adipogenic reprogramming from non-specific lipogenesis or metabolic perturbation. Moreover, our RNA-seq analysis revealed widespread transcriptomic reprogramming upon induction. A heatmap illustrates prominent changes in core adipogenic transcription factors, and GO enrichment analysis demonstrates that upregulated genes are strongly enriched in pathways related to adipogenesis and lipid metabolism, whereas downregulated genes are associated with pro-tumorigenic functions such as cell migration and angiogenesis **(Revised Fig. S3j-i)**

Although these comprehensive data clearly establish that the induced tumor cells exhibit robust adipogenic phenotypic and molecular features, we fully recognize that terminally differentiated adipocytes with fully mature *in vivo* physiological function cannot be faithfully recapitulated within a 14-day *in vitro* induction period. Therefore, we fully accept your suggestion and have revised the manuscript throughout, replacing “terminally differentiated adipocytes” with “**adipocyte-like cells (ALCs)**” to ensure accurate and responsible terminology.

Revised Fig. S3

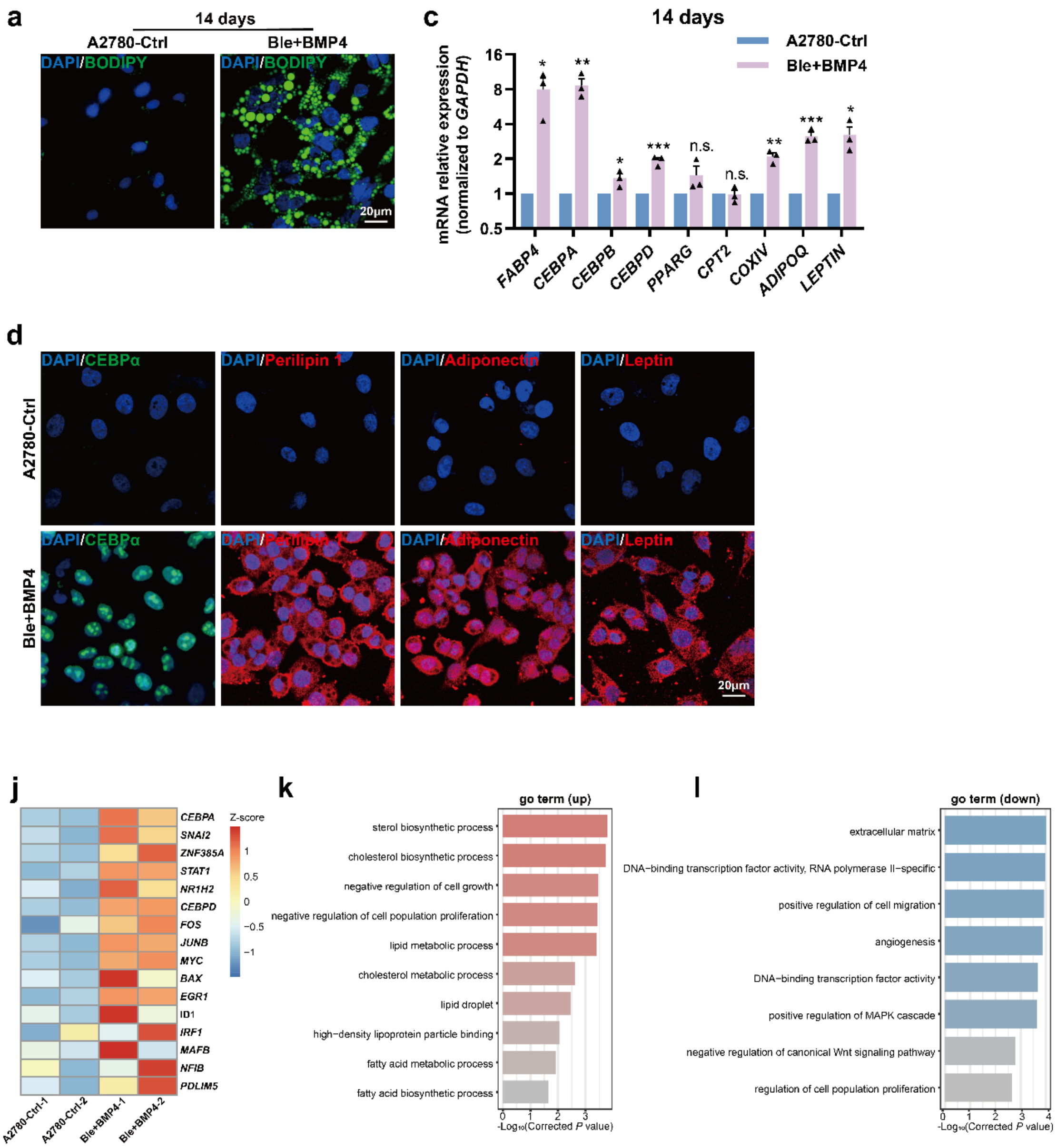

Revised Fig. S3 | Ble induces A2780 cells transdifferentiation into adipocyte-like cells and inhibits cell proliferation. a Lipid droplets in Ble+BMP4 treated A2780 cells at D14 were

visualized by BODIPY staining. Scale bars, 20 µm. … **c** qRT–PCR analysis of the mRNA levels of adipocyte-specific genes in Ble+BMP4 treated A2780 cells at D14 (*n* = 3). **d** Immunostaining for CEBPα (green), Perilipin 1(red), Adiponectin (red), Leptin (red) and nuclei (DAPI) in Ble+BMP4 treated A2780 cells at D14; scale bar, 20 µm. … **j** Heatmap showing differential expression of adipogenesis-related transcription factors in A2780 cells with or without Ble+BMP4 treatment. **k, l** Bar plot of selected functional enrichment terms of genes upregulated and downregulated in Ble+BMP4 treated A2780 cells. Data are presented as mean ± SEM. **P* < 0.05, ***P* < 0.01, ****P* < 0.001. Statistical analyses were performed using two-sided unpaired Student's *t*-test in (**c**).

2. Lipid droplet accumulation vs. adipogenesis

2.1 Data shown in Fig. S2 (ROSI treatment alone) showed stronger lipid accumulation in MEFs than Ble-treatment in terms of the number and size of Bodipy-stained cells, and the expression of PPARG/FABP4. Why do the authors stick to blebbistatin when ROSI has a stronger effect?

Response:

We sincerely appreciate your careful observation and valuable question, which helps us correct the ambiguous annotation and clarify our experimental design.

We first clarify a critical labeling point: the group denoted "iA (Rosi)" in the **Original Fig. S2** does not represent rosiglitazone treatment alone, but rather the **combined treatment of Blebbistatin + Rosiglitazone (Ble+Rosi)**. This experiment was designed to validate that MEFs can undergo adipogenic transdifferentiation via Ble combined with distinct classes of adipogenic inducers—either BMP4 or Rosi. Consistent with this design, our results confirmed that the Ble+Rosi combination induced more robust lipid accumulation and higher expression of *Pparg* and *Fabp4* compared with Ble+BMP4.

To eliminate any misinterpretation, we have revised the group label in the **Revised Fig. S2** from "iA (Rosi)" to **Ble+Rosi**, so that it precisely reflects the actual treatment regimen.

Notably, Ble serves as the core effector in our study to trigger cytoskeletal disruption, and adipogenic transdifferentiation depends on the synergistic action between Ble and adipogenic inducers (BMP4 or Rosi). This synergistic design is central to our mechanistic investigation into

how mechanical cues regulate adipogenic reprogramming, which is the primary focus of our work rather than comparing the potency of different inducers alone.

Revised Fig. S2

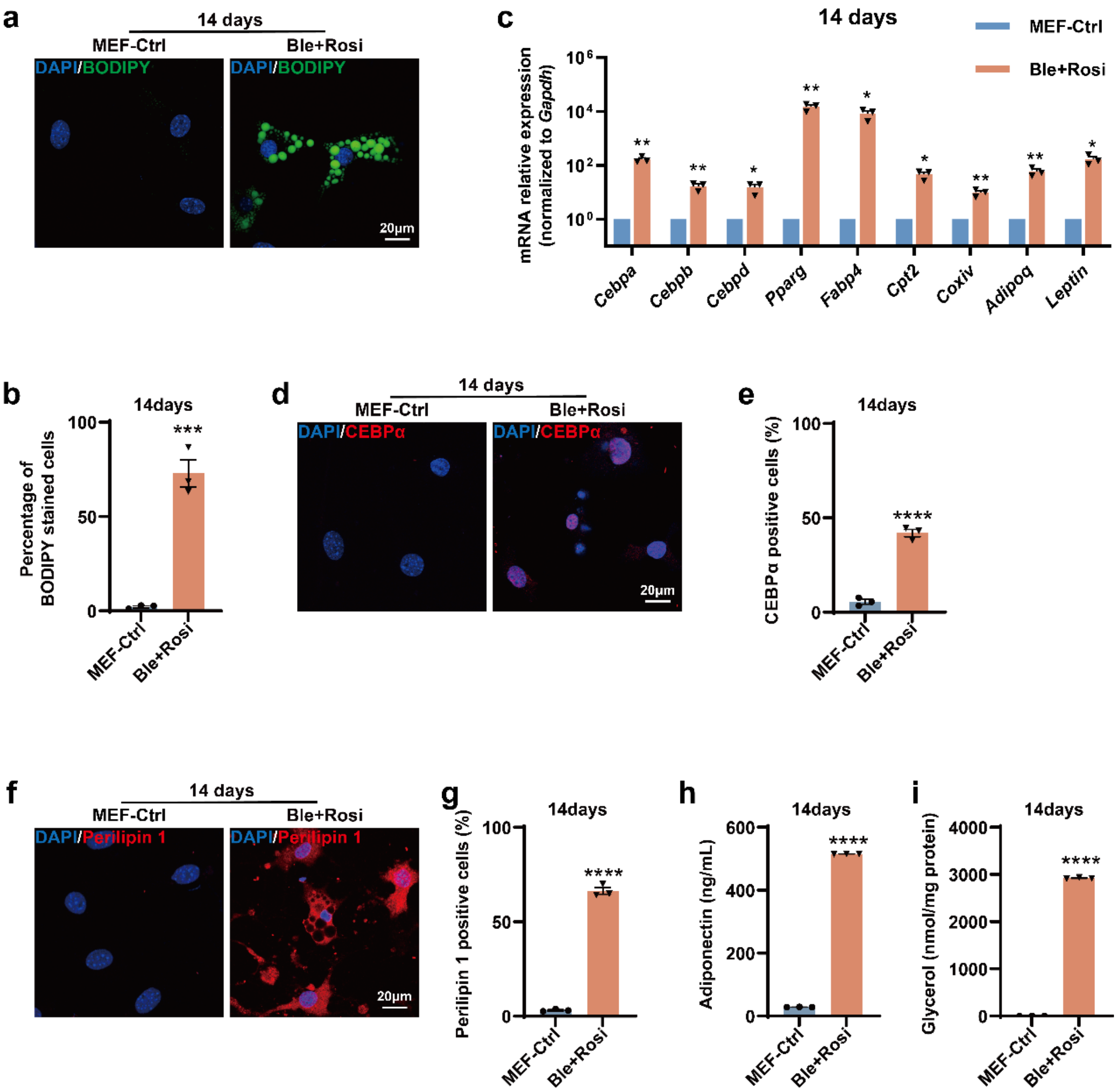

Revised Fig. S2 Ble+Rosi induces adipocyte transdifferentiation in MEFs. a Lipid droplets in Ble+Rosi treated MEFs were visualized by BODIPY staining. Scale bars, 50 μm. **b** The percentage of BODIPY-stained cells were quantified (n = 3). **c** qRT–PCR analysis of the mRNA levels of adipogenesis- and FAO-related genes in Ble+Rosi treated MEFs (n = 3). **d** Immunostaining for CEBPα (red) and nuclei (DAPI) in Ble+Rosi treated MEFs. scale bar, 50 μm. **e** CEBPα-positive cells were quantified (n = 3). **f** Immunostaining for Perilipin 1 and nuclei (DAPI) in Ble+Rosi treated MEFs. scale bars, 50 μm. **g** Perilipin 1-positive cells were quantified (n = 3). **h** Adiponectin secretion levels were measured for Ble+Rosi treated MEFs by ELISA (n = 3). **i** Isoproterenol-induced

lipolysis was measured in Ble+Rosi treated MEFs ($n = 3$). Data are presented as mean ± SEM. $*P < 0.05$, $**P < 0.01$, $***P < 0.001$, $****P < 0.0001$. Statistical analyses were performed using two-sided unpaired Student's *t*-test in (**b**, **c, e, g, h,** and **i)**.

2.2 Fig. 2A shows numerous small lipid droplets in blebbistatin-treated A2780 cells that do not exhibit in vitro adipocyte morphology or lipid droplet fusion, as observed in Fig. S2A in MEFs. The authors should explain why they believe LD accumulation indicates adipogenesis rather than being merely a consequence of increased FA synthesis to maintain survival under cytoskeletal stress. Lipid droplets: a cellular organelle vital in cancer cells | Cell Death Discovery.

Response:

We sincerely appreciate this thoughtful and rigorous comment, which allows us to more clearly distinguish **adipogenic reprogramming** from non-specific stress-induced lipid storage and strengthen our interpretation of lipid droplet morphology and function.

We sincerely appreciate your insightful question, which prompts us to further clarify that lipid droplet (LD) accumulation in Ble+BMP4-treated A2780 cells is a result of adipogenic reprogramming, rather than a stress-induced adaptive response for survival.

First, the relatively small lipid droplets observed in Ble+BMP4-treated A2780 cells do not reflect a lack of adipogenic identity, but rather a well-documented species- and cell-type-specific difference between human and mouse adipogenic models. As reported in previous studies, human adipocyte models — including cancer cells undergoing adipogenic conversion — consistently form smaller lipid droplets compared with mouse 3T3-L1 or MEFs undergoing classical adipogenesis **(PMID: 21499706)**. Our own staining further supports this pattern: human A2780 cells and human adipose-derived mesenchymal stem cells both displayed smaller lipid droplets than similarly induced mouse MEFs **(Response Fig. 1a)**. This morphological distinction is therefore a normal feature of human *in vitro* adipogenic maturation, not evidence of stress-induced lipid accumulation.

More importantly, multiple lines of functional and molecular evidence confirm that these changes reflect genuine adipogenic reprogramming, not a passive survival response to cytoskeletal stress. We observed robust upregulation of master adipogenic transcription factors (*PPARG*, *CEBPa*) and

lineage-specific adipokines (*FABP4*, *ADIPOQ*, *LEPTIN*) at both mRNA and protein levels **(Revised Fig. S3c, d)**. Critically, the induced cells also exhibited β-adrenergic agonist-stimulated lipolysis (**Revised Fig. S3g)**, a specialized metabolic function unique to mature adipocytes that is not recapitulated by non-specific fatty acid synthesis or stress-induced lipid storage.

Taken together, the small lipid droplet phenotype (consistent with human adipocyte morphology), activation of a core adipogenic transcriptional program, and acquisition of adipocyte-specific lipolytic function collectively demonstrate that lipid accumulation in A2780 cells arises from true adipogenic reprogramming, rather than a general adaptive response to cytoskeletal disruption.

Response Fig. 1

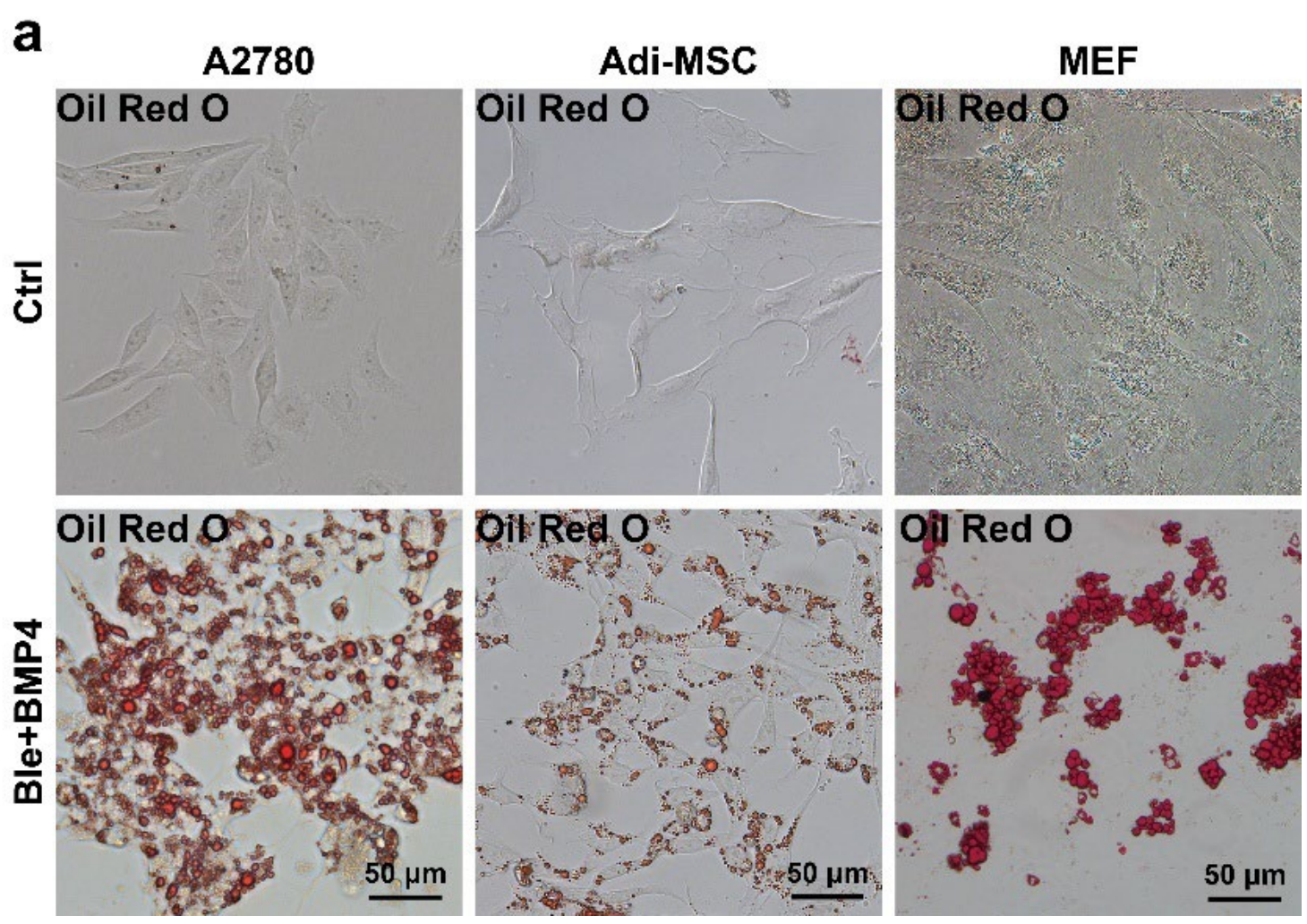

Response Fig. 1 | a Lipid droplets in Ble+BMP4 treated A2780 cells, Adi-MSCs and MEFs were visualized by Oil Red O staining. Scale bars, 50 µm.

Revised Fig. S3

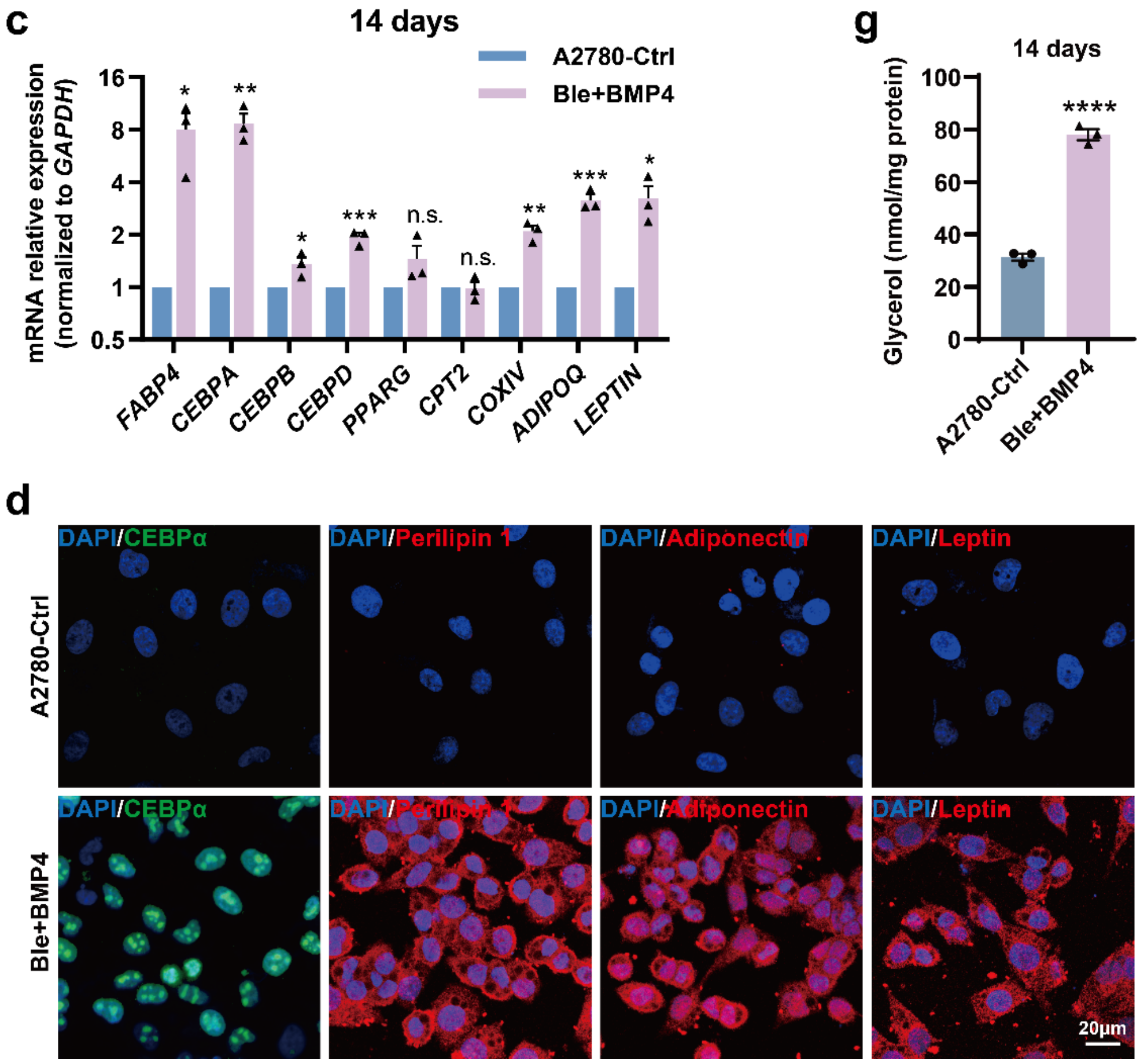

Revised Fig. S3 | Ble induces A2780 cells transdifferentiation into adipocyte-like cells and inhibits cell proliferation. … **c** qRT–PCR analysis of the mRNA levels of adipocyte-specific genes in Ble+BMP4 treated A2780 cells at D14 (n = 3). **d** Immunostaining for CEBPα (green), Perilipin 1(red), Adiponectin (red), Leptin (red) and nuclei (DAPI) in Ble+BMP4 treated A2780 cells at D14; scale bar, 20 μm. … **g** Isoproterenol-induced lipolysis was measured in Ble+BMP4 treated A2780 cells at D14 (n = 3). Data are presented as mean ± SEM. *$P < 0.05$, **$P < 0.01$, ***$P < 0.001$, ****$P < 0.0001$. Statistical analyses were performed using two-sided unpaired Student's t-test in **c** and **g**.

2.3 Also, PLIN1 staining appeared to be more cytoplasmic than LD-specific, with increased staining observed in almost all cells. At least, judging by the image presented. Does PLIN1 colocalise with LDs in Ble-treated A2780 cells?

Response:

We sincerely appreciate your careful observation of PLIN1 staining in Ble+BMP4-treated A2780 cells.

To clarify this, we conducted high-magnification confocal imaging and confirmed clear colocalization of PLIN1 and lipid droplets (LDs, BODIPY-stained) in both induced MEFs and A2780 cells **(Response Fig. 2)**.

The presence of PLIN1 in nearly all cells but LDs in only a subset is attributed to the asynchronous adipogenic reprogramming of tumor cells: PLIN1, an early adipogenic marker **(PMID: 24587346)**, is first upregulated in the cytoplasm of induced cells during the 14-day induction. LDs form and mature only in the late stage of adipogenic differentiation, after which PLIN1 translocate to the LD surface. This sequential, asynchronous progression explains the broader cytoplasmic PLIN1 signal in the overall cell population, while mature LDs appear only in a subset of cells at a given time point.

Response Fig. 2

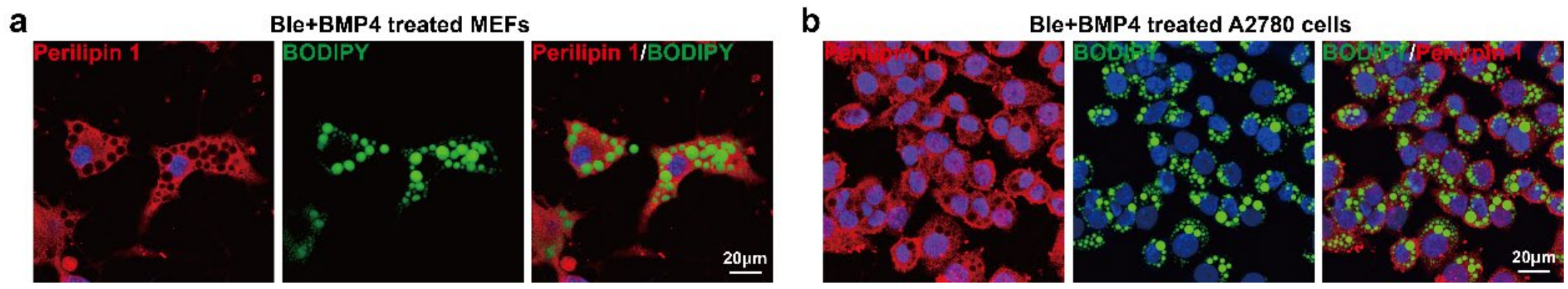

Response Fig. 2 | a. Colocalization of Perilipin 1 (red) and BODIPY-labeled lipid droplets (green) in Ble+BMP4-treated MEFs at D14; scale bar, 20 μm. **b**. Colocalization of Perilipin 1 (red) and BODIPY-labeled lipid droplets (green) in Ble+BMP4-treated A2780 cells at D14; scale bar, 20 μm.

2.4 In this context RNA-seq data from "differentiated" A2780 cells as shown for MEFs in Fig. 1F shall be provided to support a "adipocyte"-like phenotype of Ble-treated A2780 cells.

Response:

We sincerely appreciate your valuable suggestion. As requested, we now provide comprehensive RNA-sequencing (RNA-seq) data for Ble+BMP4-treated A2780 cells in **Revised Fig. S3j-l**. These data reveal widespread transcriptional reprogramming: the heatmap illustrates differential regulation of core adipogenesis-associated transcription factors (e.g., *CEBPA*, *CEBPD*) **(Revised Fig. S3j)**; GO enrichment analysis showed that the upregulated genes were significantly enriched in adipogenesis pathways (e.g., "cholesterol biosynthetic process", "lipid metabolic process", "fatty acid metabolic process") **(Revised Fig. S3k)**. Conversely, downregulated genes are strongly

associated with protumorigenic processes, including positive regulation of cell migration and angiogenesis **(Revised Fig. S3l)**—consistent with a functional shift away from cancer cell characteristics toward an adipocyte-like state.

Together, these transcriptomic data complement our previously reported phenotypic (lipid droplet accumulation), molecular (*CEBPs*, *PPARG*, *ADIPOQ*, *LEPTIN* upregulation), and functional (β-adrenergic-responsive lipolysis) evidence to robustly support the adipocyte-like identity of the induced cells **(Revised Fig. S3a, c, d, g)**. As emphasized earlier, to maintain strict scientific rigor—acknowledging that *in vitro*-induced cells do not fully recapitulate the physiological properties of *in vivo* terminally differentiated adipocytes—we continue to use the term "adipocyte-like cells (ALCs)" to describe the reprogrammed A2780 cells throughout the revised manuscript.

Revised Fig. S3

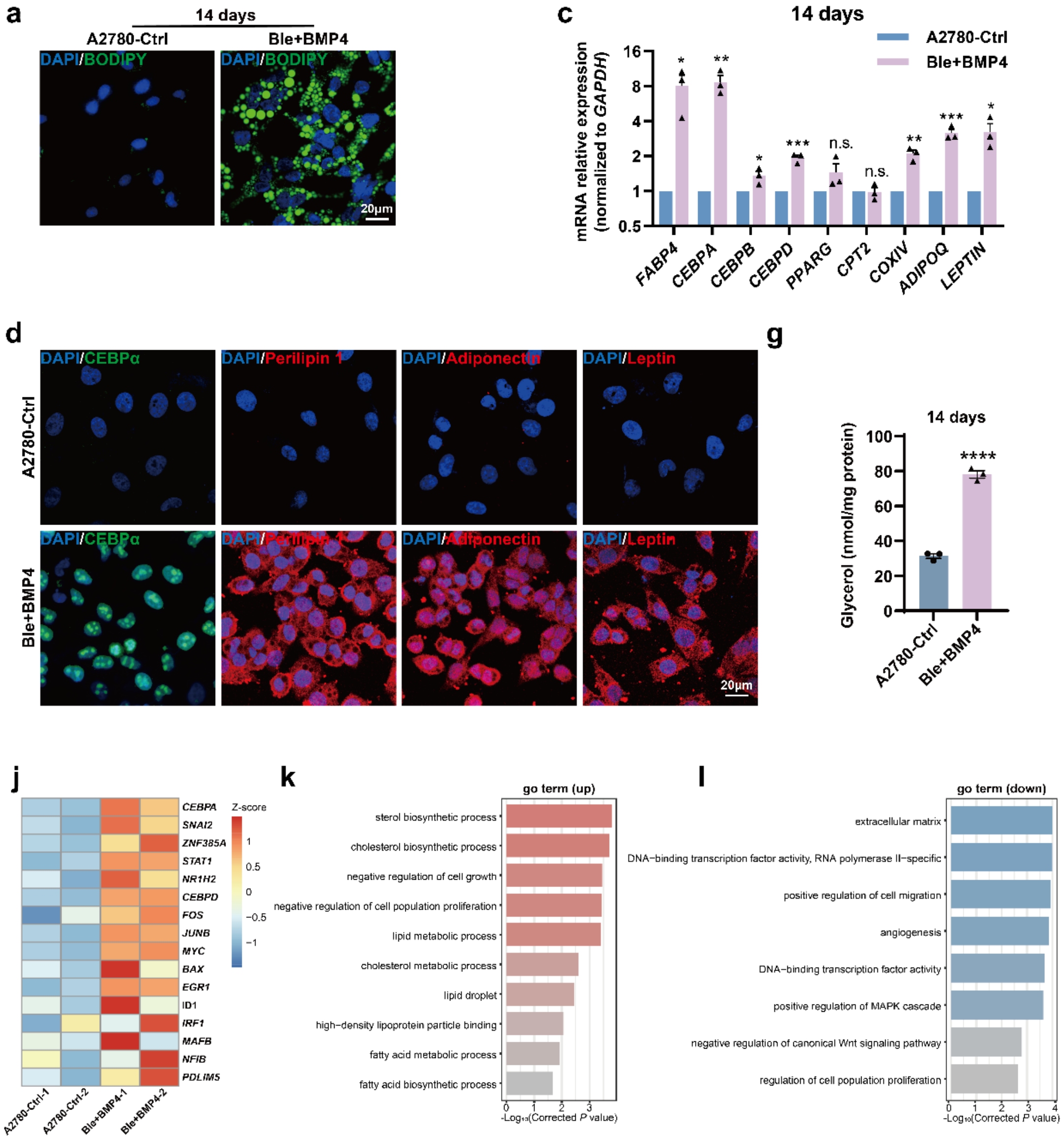

Revised Fig. S3 | Ble induces A2780 cells transdifferentiation into adipocyte-like cells and inhibits cell proliferation. a Lipid droplets in Ble+BMP4 treated A2780 cells at D14 were visualized by BODIPY staining. Scale bars, 20 μm. … **c** qRT–PCR analysis of the mRNA levels of adipocyte-specific genes in Ble+BMP4 treated A2780 cells at D14 (*n* = 3). **d** Immunostaining for CEBPα (green), Perilipin 1(red), Adiponectin (red), Leptin (red) and nuclei (DAPI) in Ble+BMP4 treated A2780 cells at D14; scale bar, 20 μm. … **g** Isoproterenol-induced lipolysis was measured in Ble+BMP4 treated A2780 cells at D14 (n = 3). … **j** Heatmap showing differential expression of adipogenesis-related transcription factors in A2780 cells with or without Ble+BMP4 treatment. **k, l** Bar plot of selected functional enrichment terms of genes upregulated and downregulated in Ble+BMP4 treated A2780 cells. Data are presented as mean ± SEM. $^*P < 0.05$, $^{**}P < 0.01$, $^{***}P$

< 0.001, ****P < 0.0001. Statistical analyses were performed using two-sided unpaired Student's t-test in (**b, c, e, f, g,** and **i**).

2.5 In Fig. 2c, the effect of Ble versus rosiglitazone (Rosi) treatment on peroxisome proliferator-activated receptor gamma (PPARG) and other differentiation markers was contrary to that observed in MEFs. What is the explanation for that?

Response:

We appreciate your comment and clarify that there is no contradiction in the expression trends of *PPARg* and other adipogenic markers between A2780 cells and MEFs. The "Ble" group in both **Original Fig. 2c** (A2780 cells) and **Original Fig. 1d** (MEFs) denotes Ble+BMP4 combined treatment, and this treatment significantly upregulated *PPARg* and other adipogenic differentiation markers in both cell types, with consistent results observed.

Original Fig. 1

d

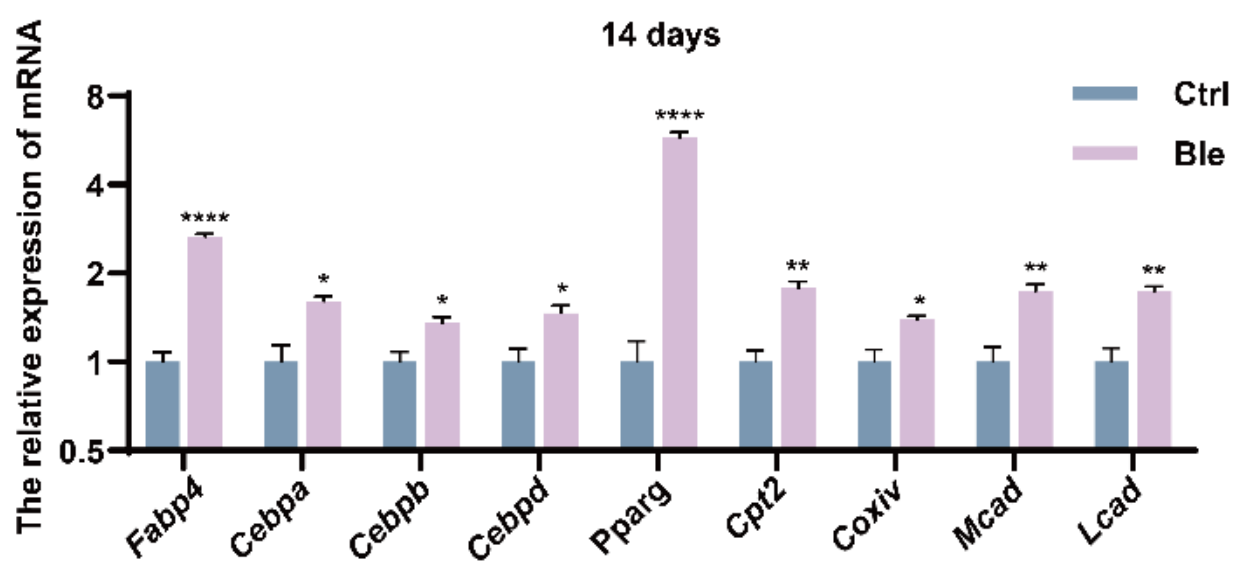

Original Fig. 2

c

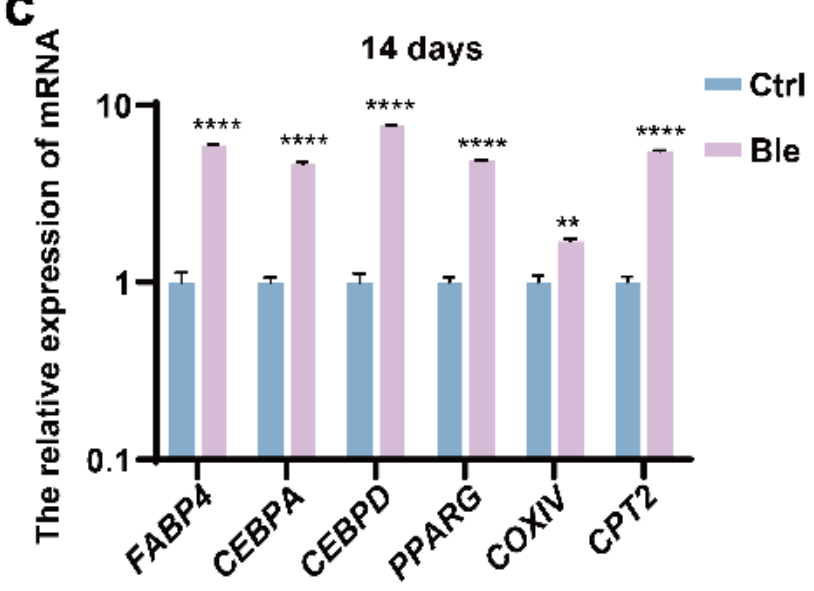

Original Fig. 1 | … **d** qRT–PCR analysis of the mRNA levels of adipogenesis- and FAO-related genes in Ble-induced adipocytes (n = 3).

Original Fig. 2 | … **c** qRT–PCR analysis of the mRNA levels of adipocyte-specific genes in Ble treated A2780 cells at D14 (n = 3).

2.6 Although there is increased PLIN1 expression in vivo (see Figures 3 and 4), the cancer cell-derived cells do not exhibit typical adipocyte morphology, characterized by large, fusing lipid droplets. The authors must explain this observation in the discussion section.

Response:

We sincerely appreciate this constructive comment, which helps us better interpret the *in vivo* adipogenic morphology of reprogrammed cancer cells.

As noted in the manuscript, PLIN1 is abundantly expressed in the cytoplasm of transdifferentiated tumor cells in vivo. Notably, large, fused lipid droplets do form *in vivo*, although they are observed in only a subset of cells. In subcutaneous tumors, these mature lipid droplets are concentrated mainly at the tumor periphery or near the injection site **(Revised Fig. 3j)**; in intraperitoneal tumors, they appear preferentially in smaller tumor nodules **(Revised Fig. 4d)**.

This pattern is consistent with previous reports of adipogenic transdifferentiation in breast cancer models (**PMID: 30645973**), and likely reflects the heterogeneity and partial nature of *in vivo* cell fate conversion. The incomplete adipogenic maturation is most likely attributable to the complex tumor microenvironment—particularly uneven drug distribution and variable exposure within large tumor masses.

We have added a discussion of this morphological heterogeneity and its underlying biological reasons in the revised Discussion section.

Revised Fig. 3

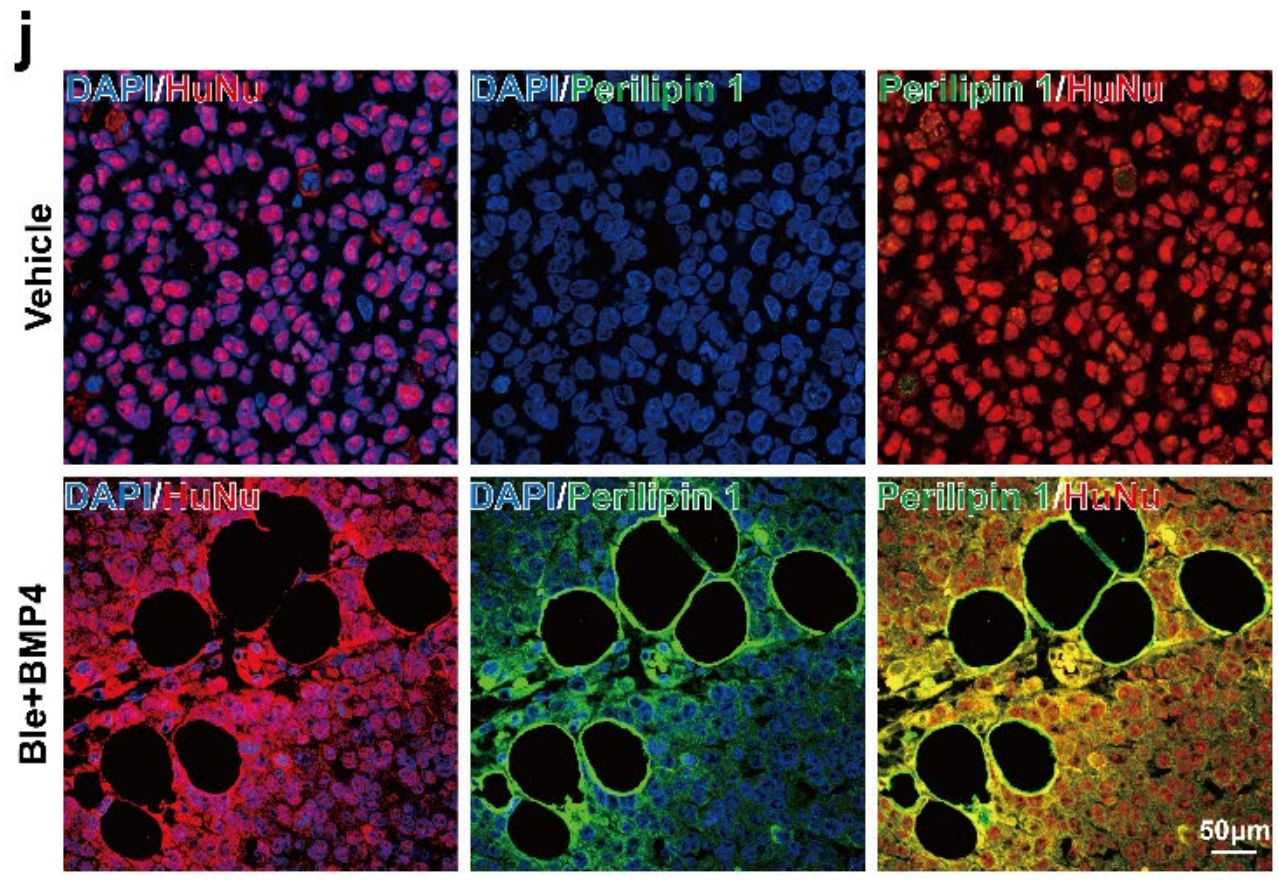

Revised Fig. 4

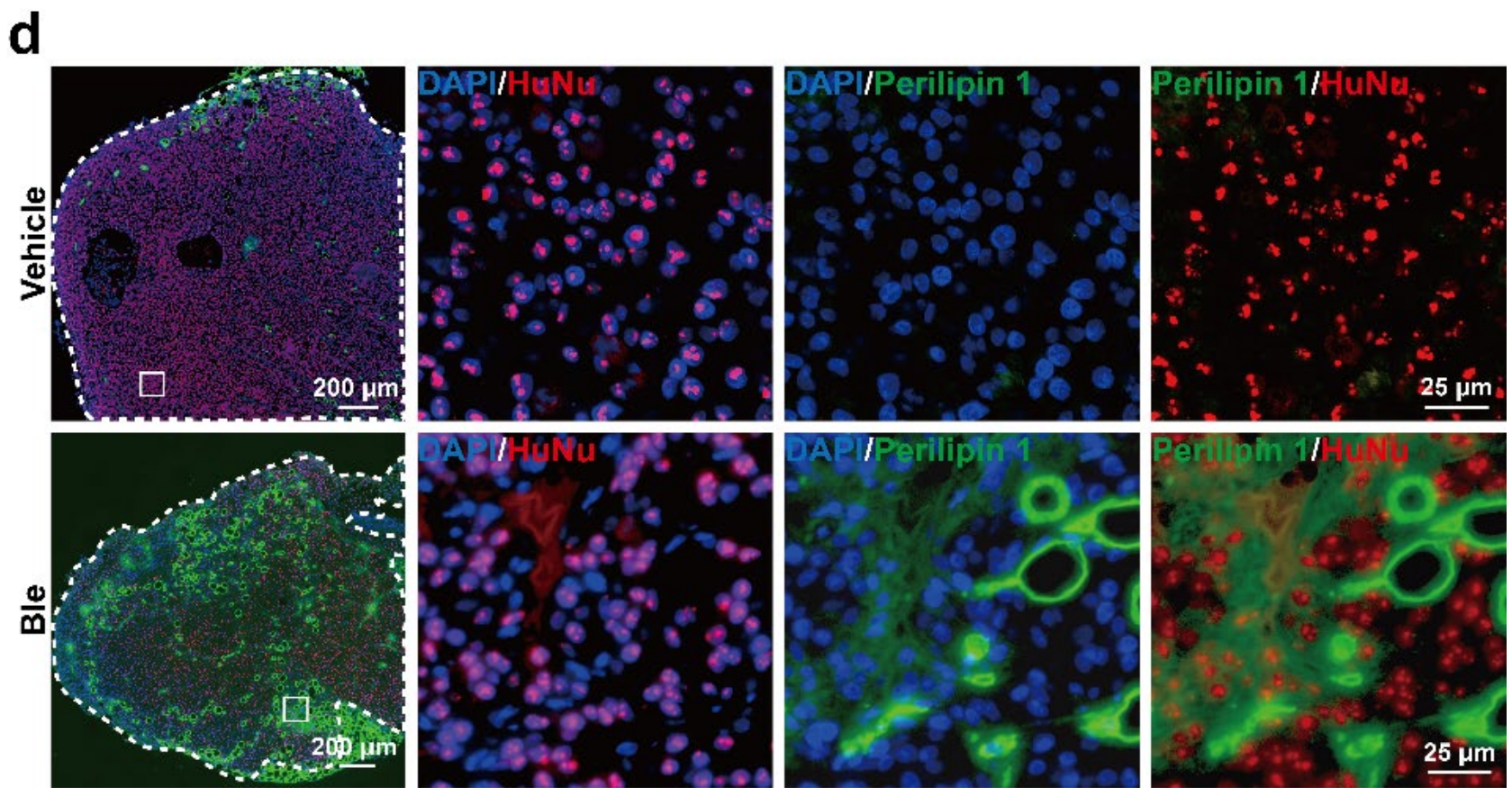

Revised Fig. 3 | … **j** Local zoom images of tumor sections that were stained with HuNu (red) and Perilipin 1 (green). Scale bars, 50 μm.

Revised Fig. 4 | …**d** Representative images of tumor sections that were stained with HuNu (red) and Perilipin 1(green). The white dotted line encircles the boundary of the tumor. Scale bars, 200 μm and 25 μm.

Revised Discussion

"…Although we have validated the anti-tumor efficacy of myosin II inhibition-mediated adipogenic transdifferentiation in subcutaneous and intraperitoneal ovarian cancer xenograft models, several limitations should be noted. … Consistent with this cellular and microenvironmental heterogeneity, the adipogenic conversion response showed clear spatial variation in vivo, with mature lipid droplets mainly observed at the tumor periphery or in small nodules, likely due to uneven intratumoral drug distribution that limits uniform transdifferentiation efficiency. …"

3. Gene expression analysis

3.1 In Fig. 2d the authors showed that "ACTB" is downregulated in RNAseq data. This makes this gene unsuitable as a reference gene for quantitative real time PCR. Please re-analyze with validated stable reference genes (for example see Reliable reference genes for expression analysis of proliferating and adipogenically differentiating human adipose stromal cells - PubMed).

Response:

We sincerely appreciate this important and professional comment.

As observed in our RNA-seq data, *ACTB* expression was indeed significantly downregulated following Ble+BMP4 treatment, likely due to Ble-mediated disruption of cytoskeletal architecture. We fully agree that ACTB is therefore not suitable as a reference gene for qRT-PCR normalization in this adipogenic transdifferentiation system.

Following the suggested literature and our re-evaluation of stably expressed genes, we selected ***GAPDH*** as an alternative reference gene. As validated in the recommended study **(PMID: 30815013)**, *GAPDH* exhibits acceptable stability during both proliferation and adipogenic differentiation of human stromal cells. Importantly, our RNA-seq data further confirmed that GAPDH expression remained unchanged under Ble+BMP4 treatment.

Accordingly, we have re-analyzed all qRT-PCR data using *GAPDH* as the validated reference gene and updated the corresponding results in the revised manuscript.

3.2 Clarify what is meant by "relative expression of mRNA", shown are fold changes vs. ctr of housekeeping genes normalized values?

Response:

We sincerely appreciate your careful comment.

The "relative mRNA expression" shown in our figures represents the fold change relative to the Ctrl group, normalized to stably expressed reference genes (*GAPDH*) using the $2^{-\Delta\Delta Ct}$ method. The expression level of the Ctrl group was set to 1, and the values in treated groups indicate the relative fold change compared with Ctrl.

To eliminate any ambiguity, we have revised the label of the y-axis to **"mRNA relative expression (normalized to *GAPDH*)"** in all corresponding figures.

3.3 Indicate whether SE or SD are shown in the figure legends?

Response:

We apologize for the missing information in the figure legends. As stated in the "Quantification and statistical analysis" section, **data are presented as mean ± SEM.** We have added this description to all corresponding figure legends in the revised manuscript.

4. Cell line choice & Methodological clarification

4.1 In figure 3, Ble was used always used in combination with BMP4 or ROSI but not administrated alone while in Figure 4 only Ble was used. Is there a reason for change in treatment strategy?

Response:

We sincerely appreciate your careful question regarding the treatment strategy.

The different regimens used in **Original Fig. 3** and **Fig. 4** reflect the distinct *in vivo* microenvironments of the tumor models.

In the subcutaneous tumor model (**Original Fig. 3**), the local microenvironment lacks endogenous adipogenic signals. We therefore combined Ble with BMP4 or Rosi to efficiently drive adipogenic transdifferentiation.

By contrast, in the intraperitoneal tumor model (**Original Fig. 4**), the peritoneal cavity — particularly the omentum — is highly enriched in adipose tissue and provides a native adipogenic niche for ovarian cancer cells, consistent with their well-characterized lipophilic metastatic behavior **(PMID: 30568223)**. In this adipose-rich milieu, Ble alone is sufficient to trigger adipogenic transdifferentiation without exogenous adipogenic inducers.

This experimental design leverages the inherent lipotropic nature of ovarian cancer cells and their adaptation to the fat-rich peritoneal microenvironment.

4.2 In Figure 3, the author used A2780 (therapy-sensitive) for the subcutaneous xenograft model, while SKOV3 (therapy-resistant) was used for intraperitoneal injection. Firstly, the main text and figure legends do not state that different cell types have been used. The authors must consistently refer to the cell types used in the figure captions and the main text to avoid confusing the reader. Secondly, there is no in vitro data showing that SKOV3 cells can "transdifferentiate" in a manner similar to A2780 cells. The authors must provide evidence showing whether or not SKOV3 behave similarly to A2780 cells in response to Ble-treatment.

Response:

We sincerely appreciate your valuable and rigorous comments, which have helped us improve the clarity of our experimental descriptions and strengthen the comprehensiveness of our data.

First, we apologize for the ambiguity regarding the cell lines used in different *in vivo* models. We have now **explicitly specified** in both the main text and corresponding figure legends that A2780 cells (therapy-sensitive) were used for subcutaneous xenografts (**Original Fig. 3**) and SKOV3 cells (therapy-resistant) for intraperitoneal models (**Original Fig. 4**), ensuring consistency and eliminating potential confusion for readers.

Notably, the therapy-sensitive or -resistant phenotype of these cell lines does not affect our core conclusion, as our strategy focuses on inducing cell **fate reprogramming toward adipocyte-like**

cells—a process initiated by Ble-mediated cytoskeletal disruption and promoted by adipogenic cues, rather than conventional chemotherapeutic killing.

To address the critical point of whether SKOV3 cells exhibit similar adipogenic transdifferentiation potential to A2780 cells, we have supplemented comprehensive *in vitro* validation data in **Revised Fig. 2**. These results demonstrate that Ble-based induction effectively triggers adipogenic transdifferentiation in SKOV3 cells, as evidenced by: (1) robust lipid droplet formation (BODIPY/Oil Red O staining); (2) significant upregulation of core adipogenic markers at both mRNA and protein levels; (3) increased secretion of adiponectin (detected by ELISA); (4) enhanced β-adrenergic-responsive lipolytic activity; and (5) reduced cell proliferation. Consistent with these phenotypic and functional changes, RNA-seq analysis further confirms: (a) a heatmap showing upregulation of key adipogenic transcription factors; (b) GSEA verifying significant enrichment of the "positive regulation of fat cell differentiation" gene set; and (c) GO enrichment analysis revealing that upregulated genes are enriched in lipid metabolism and adipogenesis pathways, while downregulated genes are associated with cell cycle progression, proliferation, and migration—mirroring the transdifferentiation signature observed in A2780 cells.

Collectively, these supplementary data confirm that Ble combined with adipogenic cues can induce adipogenic transdifferentiation in both A2780 and SKOV3 cells, regardless of their inherent therapy-resistant phenotype. This consistency underscores the generality of our cytoskeleton-mediated adipogenic reprogramming strategy across ovarian cancer cell lines with distinct clinical characteristics.

Revised Fig. 2

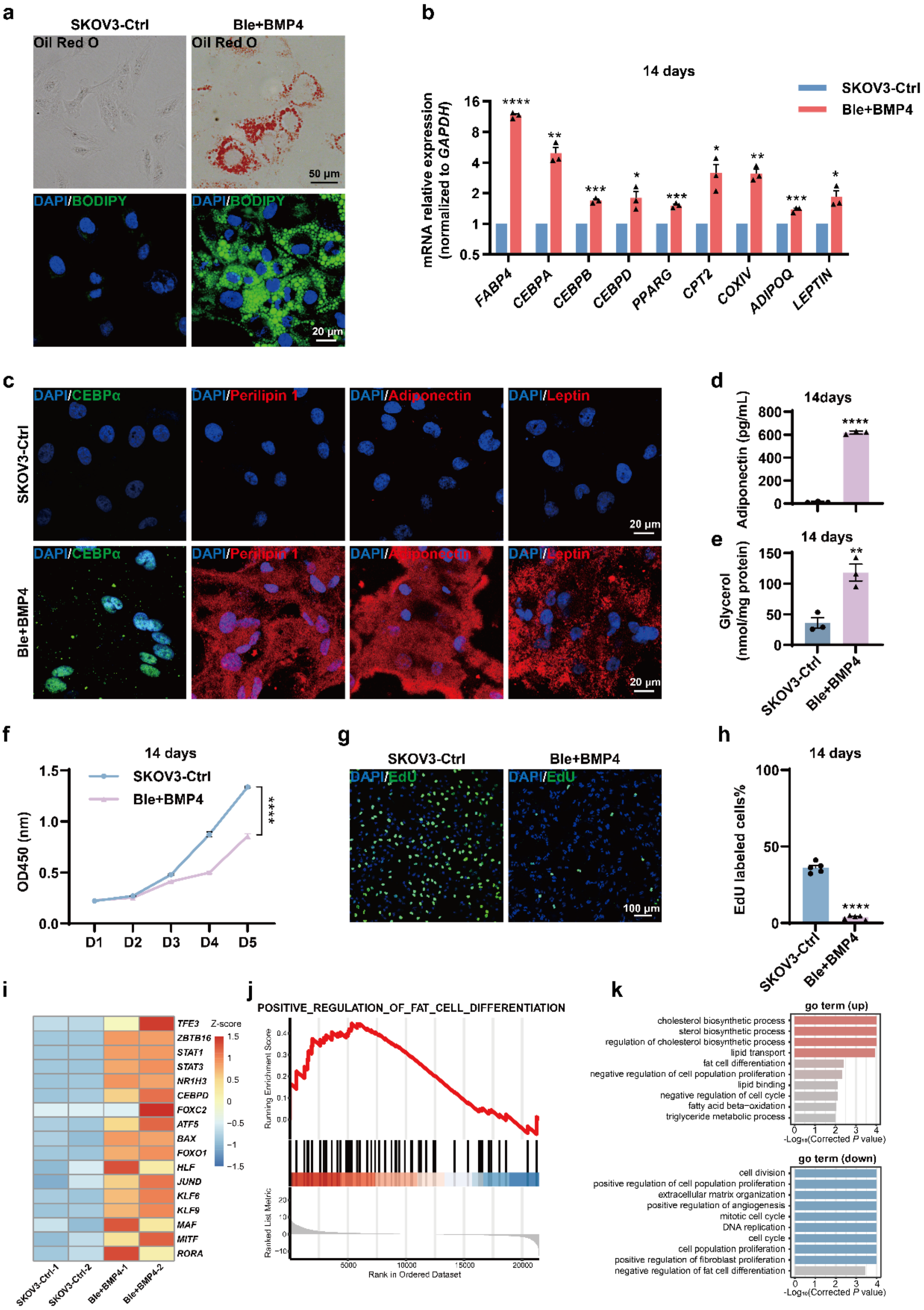

Fig. 2 | Ble induces SKOV3 cells transdifferentiation into adipocyte-like cells and inhibits cell proliferation. a Lipid droplets in Ble+BMP4 treated SKOV3 cells at D14 were visualized by Oil Red O and BODIPY staining. Scale bars, 50 μm and 20 μm. **b** qRT–PCR analysis of the mRNA levels of adipocyte-specific genes in Ble+BMP4 treated SKOV3 cells at D14 (n = 3). **c**

Immunostaining for CEBPα (green), Perilipin 1(red), Adiponectin (red), Leptin (red) and nuclei (DAPI) in Ble+BMP4 treated SKOV3 cells at D14; scale bar, 20 μm. **d** Adiponectin secretion levels were measured for Ctrl and Ble+BMP4 treated SKOV3 cells by ELISA (n = 3). **e** Isoproterenol-induced lipolysis was measured in Ble+BMP4 treated SKOV3 cells at D14 (n = 3). **f** CCK-8 analysis of Ble+BMP4 treated SKOV3 cells revealed a lower cell proliferation rate (n = 4). **g, h** Ble+BMP4 treated SKOV3 cells were incubated with 5-ethynyl-2'-deoxyuridine (EdU) for 2.5 hr to label proliferating cells. EdU-labeled cells were quantified using ImageJ (n = 5), scale bar, 100 μm. **i** Heatmap illustrating the expression patterns of key adipogenesis-related transcription factors in Ctrl and Ble+BMP4 groups. **j** GSEA enrichment plot showing significant enrichment of the "positive regulation of fat cell differentiation" gene set in SKOV3 cells upon Ble+BMP4 treatment (NES: 1.5132473, FDR: 0.0782314). **k** GO enrichment analysis showing selected biological processes for upregulated and downregulated genes in Ble+BMP4 treated SKOV3 cells. Data are presented as mean ± SEM. *P < 0.05, **P < 0.01, ***P < 0.001, ****P < 0.0001. Statistical analyses were performed using two-sided unpaired Student's t-test in (**b**, **d**, **e**, **h**) and two-way ANOVA in (**f**).

Minor points.

1. Fig. 1f: Specify the selection criteria for the "top 50 differentially expressed genes."

Response:

We sincerely appreciate your careful comment. In **Original Fig.1f**, we presented a heatmap of selected adipocyte-specific and MEF-specific genes, rather than the top 50 differentially expressed genes. These genes were manually selected based on their known specific expression in adipocytes or MEFs, as stated in the figure legend. We have clarified this description further in the revised manuscript to avoid any confusion **(Revised Fig. 1h)**.

Revised Figure legends:

"**Revised Fig. 1** |… **h** Heatmap of selected adipocyte-specific and MEF-specific gene expression in Ble+BMP4 treated MEFs."

2. Fig. 3b / Fig. S4a: provide statistical details (p-values; specify whether SE or SD is shown).

Response:

We sincerely appreciate your comment. Data in **Original Fig. 3b** and **Original Fig. S4a** are presented as mean ± SEM, and the corresponding *P*-values have been added in the revised figure legends.

Revised Figure legends:

"**Revised Fig. 3 |** … **b** Tumor volume growth curves are shown during the treatment time course (n = 8 for each group, $P = 0.4346$ for Vehicle *vs* BMP4 alone, $P = 0.7985$ for Vehicle *vs* Ble alone, $P < 0.0001$ for Vehicle *vs* Ble+BMP4). …Data are presented as mean ± SEM. …Statistical analyses were performed using two-way ANOVA in (**b**) …"

"**Revised Fig. S5 | a** Tumor volume growth curves are shown during the treatment time course (n = 8 for each group, $P = 0.0679$ for Vehicle *vs* Rosi alone, $P = 0.8520$ for Vehicle *vs* Ble alone, $P < 0.0001$ for Vehicle *vs* Ble+Rosi). …Data are presented as mean ± SEM. …Statistical analyses were performed using two-way ANOVA in (**a**). …"

3. Fig. 3j: Lipid droplet morphology is not visible; please explain.

Response:

We appreciate your careful observation. The apparent lack of distinct lipid droplet morphology in Original Fig. 3j might be attributed to the **heterogeneity and partial nature of adipogenic transdifferentiation in the complex *in vivo* tumor microenvironment**—a point we have expanded on in our response to **Comment 2.6**.

Notably, while large, mature lipid droplets do form in subcutaneous tumors following Ble+BMP4/Rosi treatment, they are not uniformly distributed across the entire tumor mass. As supplemented in **Revised Fig. 3j & Fig. 4d)**. These lipid droplets are concentrated primarily at the tumor periphery (where drug exposure and adipogenic cues are more favorable) or in smaller tumor nodules, rather than in the central regions of larger tumors. This spatial heterogeneity—consistent with previous reports of in vivo cell fate reprogramming (PMID: 30645973)—explains why distinct lipid droplets are less apparent in the representative field shown in **Original Fig. 3j**.

We have clarified this spatial distribution and the underlying reasons for morphological heterogeneity in the revised Discussion section, with supporting data provided in the revised figures.

Revised Fig. 3

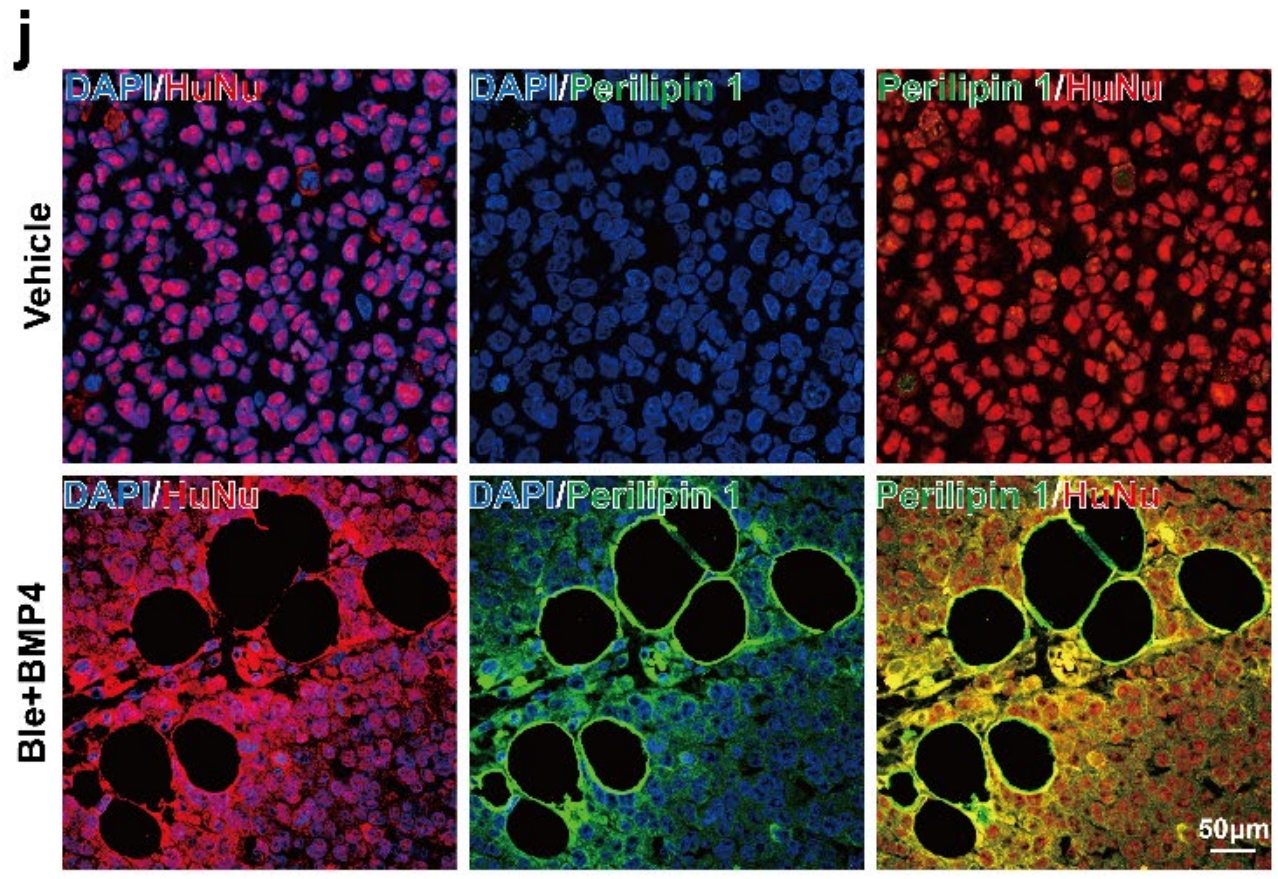

Revised Fig. 4

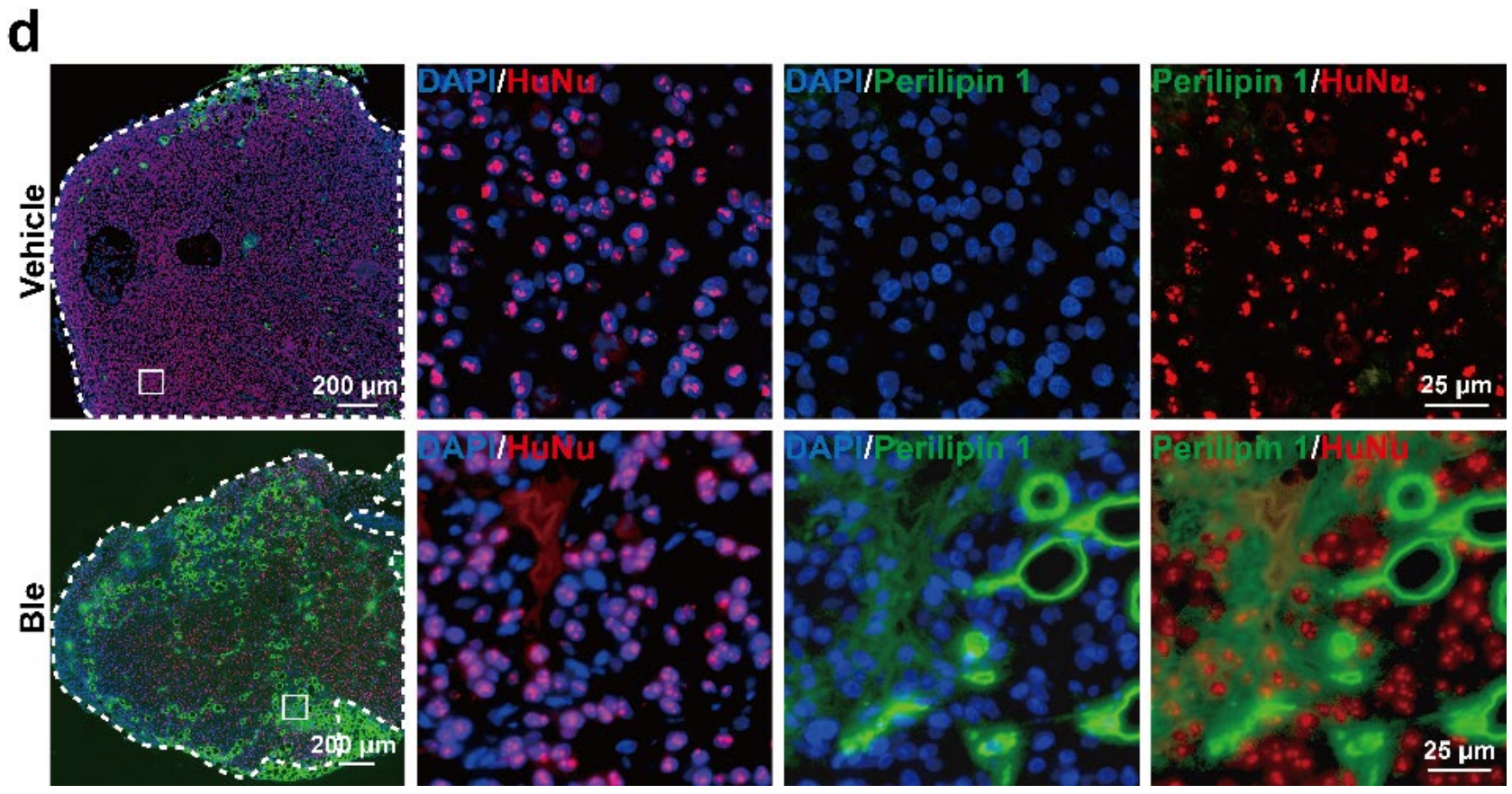

Revised Fig. 3 | … **j** Local zoom images of tumor sections that were stained with HuNu (red) and Perilipin 1 (green). Scale bars, 50 μm.

Revised Fig. 4 | …**d** Representative images of tumor sections that were stained with HuNu (red) and Perilipin 1(green). The white dotted line encircles the boundary of the tumor. Scale bars, 200 μm and 25 μm.

Revised Discussion

"…Although we have validated the anti-tumor efficacy of myosin II inhibition-mediated adipogenic transdifferentiation in subcutaneous and intraperitoneal ovarian cancer xenograft models, several limitations of the current study should be not. … Third, the complex tumor microenvironment leads to heterogeneous therapeutic responses and incomplete adipogenic maturation in vivo: large, fused lipid droplets typical of mature adipocytes are only observed in a subset of cells, preferentially at

the tumor periphery or in smaller nodules, likely due to uneven intratumoral drug distribution and variable exposure. …"

4. Oil Red O staining: clarify whether the peripheral staining in mouse tissue reflects tumor-associated lipid storage.

Response:

We appreciate your thoughtful comment.

We agree that Oil Red O staining alone cannot definitively distinguish between general tumor-associated lipid storage and lipid droplets generated during adipogenic transdifferentiation. Notably, the lipid-rich regions in tumor tissues were consistently positive for Perilipin1, a specific protein that coats lipid droplets in mature adipocytes and is not typically present in tumor cells with only passive lipid accumulation **(PMID: 20081801)**.

Combined with our robust *in vitro* evidence showing that Ble efficiently drives adipogenic transdifferentiation of A2780 and SKOV3 cells, as well as the reduced malignant phenotype of tumor cells *in vivo*, the most reasonable interpretation is that the Oil Red O-positive signals reflect ***de novo* lipid droplet formation during cancer cell adipogenic transdifferentiation**, rather than nonspecific lipid accumulation.

5. Line 210 / Fig. 4d: clarify whether Ble+BMP4-induced "transdifferentiation" is local, and explain what the perilipin+ areas in the vehicle controls represent.

Response:

We appreciate your careful observation and insightful question.

First, the adipogenic transdifferentiation induced by Ble+BMP4 (described in Line 210 and **Original Fig. 3**) is indeed localized, occurring predominantly at the tumor periphery and near injection sites. This pattern reflects restricted drug diffusion within the solid tumor microenvironment following intratumoral and peritumoral administration.

Regarding the Perilipin 1-positive areas in the vehicle control group of Original Fig. 4d, these signals do not colocalize with HuNu (human nuclear antigen). This clearly demonstrates that these PLIN1-positive cells are of mouse host origin, not transdifferentiated human tumor cells. This

background staining is consistent with the well-established lipophilic nature of ovarian cancer metastasis in the murine abdominal microenvironment, where host adipose tissue is abundant **(PMID: 30568223)**.

6. Fig. 3h vs. Fig. 4d: clarify whether outcomes depend on tumor cell administration route or treatment condition.

Response:

We appreciate your careful comparison and thoughtful question.

The differences observed between **Original Fig. 3h** (subcutaneous) and **Original Fig. 4d** (intraperitoneal) primarily reflect distinct tumor microenvironments shaped by different inoculation routes, rather than fundamental discrepancies in treatment efficacy. In the subcutaneous model (Fig. 3h), the local tissue lacks endogenous adipogenic signals, so Ble combined with exogenous BMP4 was required to drive efficient adipogenic transdifferentiation. By contrast, in the intraperitoneal model (Fig. 4d), the omentum provides a naturally fat-rich, pro-adipogenic niche. In this permissive environment, Ble alone was sufficient to induce transdifferentiation in small metastatic nodules.

Together, both models consistently demonstrate that Ble can trigger effective adipogenic transdifferentiation of tumor cells when paired with either exogenous or endogenous adipogenic cues, depending on the *in vivo* context.

7. Line 273: specify in the main text that ChIP-qPCR was used to demonstrate CEBPα/PPARγ binding to HP1/LMNA/C.

Response:

We appreciate your constructive comment. We have now explicitly stated in the main text (Line 273) that ChIP-qPCR was used to demonstrate the binding of *CEBPα*/*PPARγ* to HP1, LMNA/C.

Revised manuscript:

"Next, we examined the binding of adipogenic transcription factors (*Cebpa*, *Cebpb*, *Cebpd*, and *Pparg*) to the heterochromatin marker HP1 and the nuclear lamina protein LMNA/C in this permissive chromatin environment by ChIP-qPCR."

8. Line 306 / Fig. 6: specify which cell type was cultured on soft vs. stiff matrices (A2780 vs. MEFs). Clarify whether adipogenesis was induced solely by substrate stiffness or if additional cocktails were used.

Response:

We appreciate your careful comment.

We have now explicitly stated in the revised main text (Line 306) and corresponding figure legend that **MEFs were used for the soft *vs* stiff matrix culture experiments shown in Original Fig. 6**. In this system, adipogenic reprogramming was induced by **substrate stiffness combined with adipogenic inducers (BMP4 or Rosi)**, consistent with the induction strategy used throughout the study. Both a soft matrix and Ble treatment act by loosening chromatin structure, thereby enhancing accessibility for adipogenic transcription factors and promoting differentiation.

To further strengthen our conclusion, we have added new supporting data showing that culturing ovarian cancer cells (A2780, SKOV3, OVCAR3) on soft matrices in the presence of BMP4 is sufficient to trigger lipid droplet formation (Oil Red O staining) and upregulation of adipogenic marker **(Response Fig. 3)**.

All cell types and experimental conditions have been clarified in the revised figure legends for full transparency.

Response Fig. 3

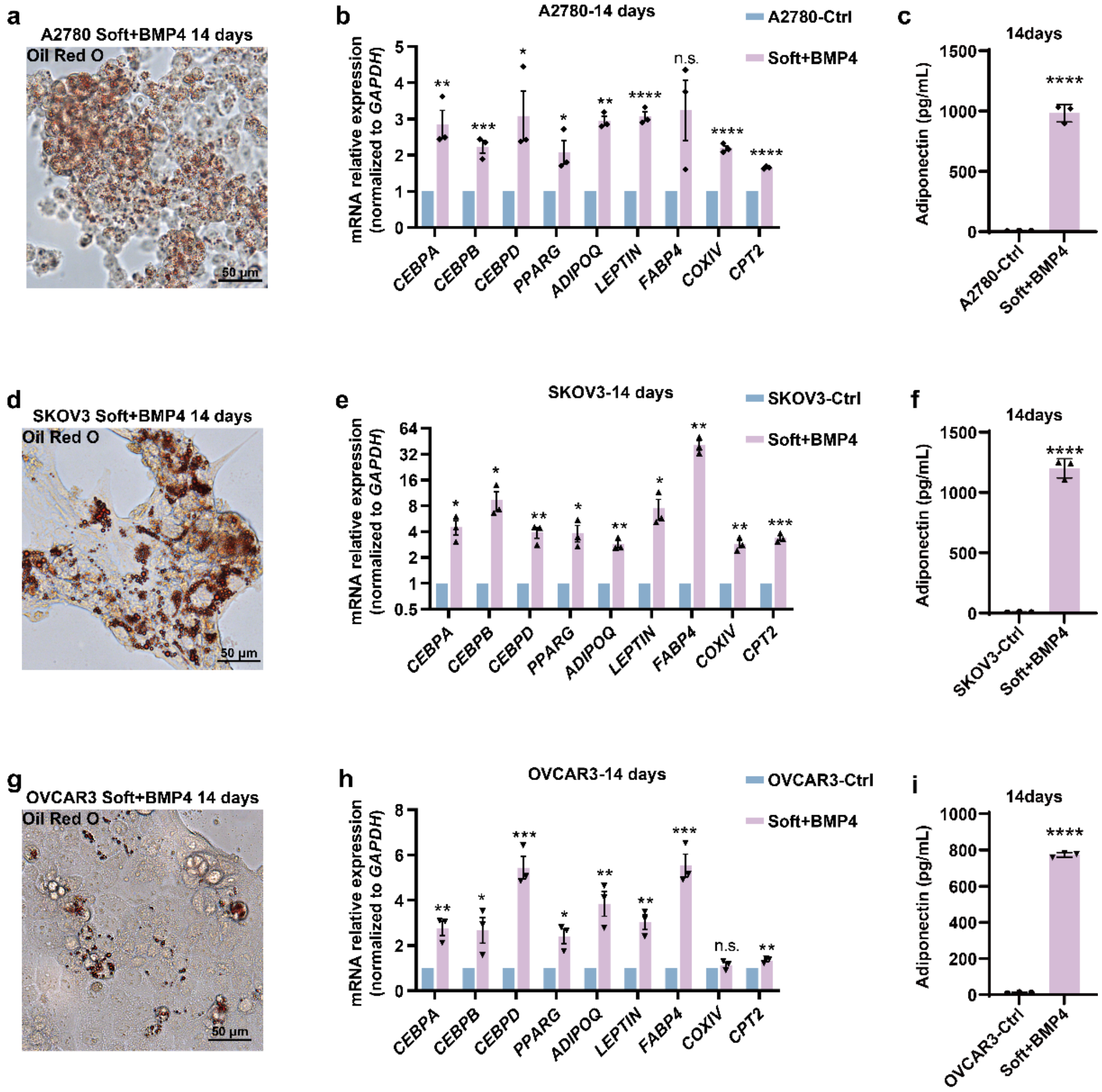

Response Fig. 3 | a Lipid droplets of A2780 cells cultured on soft substrates with BMP4 for 14 days were visualized by Oil Red O staining. Scale bars, 50 μm. **b** qRT–PCR analysis of the mRNA levels of adipogenesis- and FAO-related genes in A2780 cells cultured on soft substrate with BMP4 (*n* = 3). **c** Adiponectin secretion levels were measured for A2780 cells cultured on soft substrate with BMP4 by ELISA (*n* = 3). **d** Lipid droplets of SKOV3 cells cultured on soft substrates with BMP4 for 14 days were visualized by Oil Red O staining. Scale bars, 50 μm. **e** qRT–PCR analysis of the mRNA levels of adipogenesis- and FAO-related genes in SKOV3 cells cultured on soft substrate with BMP4 (*n* = 3). **f** Adiponectin secretion levels were measured for SKOV3 cells cultured on soft substrate with BMP4 by ELISA (*n* = 3). **g** Lipid droplets of OVCAR3 cells cultured on soft substrates with BMP4 for 14 days were visualized by Oil Red O staining. Scale bars, 50 μm. **h** qRT–PCR analysis of the mRNA levels of adipogenesis- and FAO-related genes in OVCAR3 cells cultured on soft substrate with BMP4 (*n* = 3). **i** Adiponectin secretion levels were measured for

OVCAR3 cells cultured on soft substrate with BMP4 by ELISA. ($n = 3$). Data are presented as mean ± SEM. *$P < 0.05$, **$P < 0.01$, ***$P < 0.001$, ****$P < 0.0001$. Statistical analyses were performed using two-sided unpaired Student's *t*-test in (**b**, **c**, **e**, **f**, **h,** and **i**).

9. Substrate stiffness: While it is true that 64 kPa (the Young's modulus of young cartilage) is soft relative to plastic (~GPa), it is much stiffer than adipose tissue (~0.5 - 2 kPa). What is the biological relevance of a 64 kPa 'soft' surface, and how does it relate to the adipogenic niche?

Response:

We appreciate this highly insightful and scientifically rigorous comment.

We agree that native adipose tissue exhibits a physiological stiffness of ~0.5–2 kPa. In our original design, 64 kPa was used as a soft substrate relative to rigid plastic, which is sufficient to reduce cytoskeletal tension and open chromatin structure, similar to the effect of Ble.

To better mimic the physiological adipogenic niche, we performed additional experiments on 2 kPa matrices (Sigma, 5141), which yielded comparable results: enhanced lipid accumulation, upregulation of adipogenic markers, and functional lipolysis in MEFs **(Response Fig. 4)**, as well as adipogenic transdifferentiation in ovarian cancer cells **(Response Fig. 3)**.

These results confirm that physiologically relevant soft matrices act similarly to 64 kPa substrates by relaxing cytoskeletal tension, thereby facilitating adipogenic transdifferentiation in cooperation with adipogenic cues.

Response Fig. 4

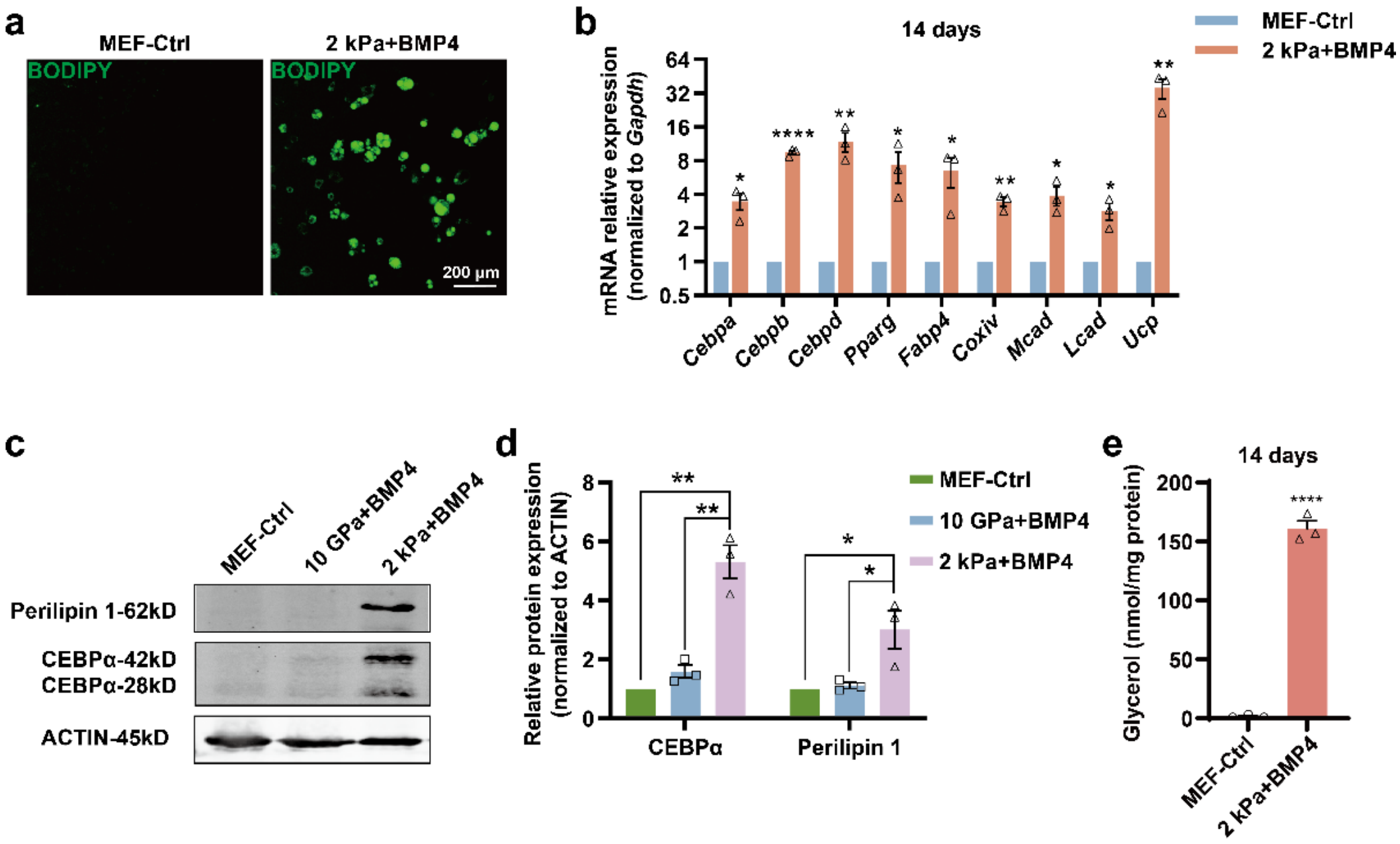

Response Fig. 4 | a Lipid droplets of cells cultured on stiff (10Gpa) and 2 kPa substrates for 14 days were visualized by BODIPY staining. Scale bars, 200 μm. **b** qRT–PCR analysis of the mRNA levels of adipogenesis- and FAO-related genes in cells cultured on 2 kPa substrate (*n* = 3). **c** Western blot analysis of CEBPα and Perilipin 1 in cells cultured on stiff (10Gpa) and 2 kPa substrates for 14 days. **d** Quantitative analysis of **c**, the relative protein expression of CEBPα and Perilipin 1 (*n* = 3). **e** Isoproterenol-induced lipolysis was measured in cells cultured on stiff (10 Gpa) and 2 kPa substrates for 14 days (*n* = 3). Data are presented as mean ± SEM. *$P < 0.05$, **$P < 0.01$, ***$P < 0.001$, ****$P < 0.0001$. Statistical analyses were performed using two-sided unpaired Student's *t*-test in (**b**, **d**, and **e**).

10. Overexpression system: Describe the system used to overexpress the YAP-5SA mutant. A control (e.g. YAP wt) is missing to confirm that the YAP-5SA mutant indeed negates the effects of the BLE on cells.

Response:

We appreciate your constructive comment.

The YAP-5SA constitutively active mutant was overexpressed in MEFs using a lentiviral expression system. We have added this description to the figure notes and methods materials in the revised version.

To rigorously validate its specific effect, we have now included wild-type YAP (YAP-WT) as an additional control group **(Revised Fig. 6k)**.

Immunofluorescence showed that cells overexpressing YAP-WT (green) remained positive for Perilipin 1 (red) after induction, whereas those expressing constitutively nuclear YAP-5SA showed no Perilipin 1 signal.

These new control data directly confirm that cytoplasmic translocation of YAP is required for Ble-mediated adipogenic transdifferentiation, and that sustained nuclear YAP activity is sufficient to block this reprogramming process.

Revised manuscript:

"… To investigate whether Ble-induced adipogenic fate transition depends on YAP nuclear export, we generated stable cell lines overexpressing a constitutive nuclear-localized YAP mutant (YAP-5SA) via lentiviral transduction. In this mutant, the phosphorylation sites required for cytoplasmic retention are mutated to alanine, thus preventing nuclear export."

Revised Fig. 6

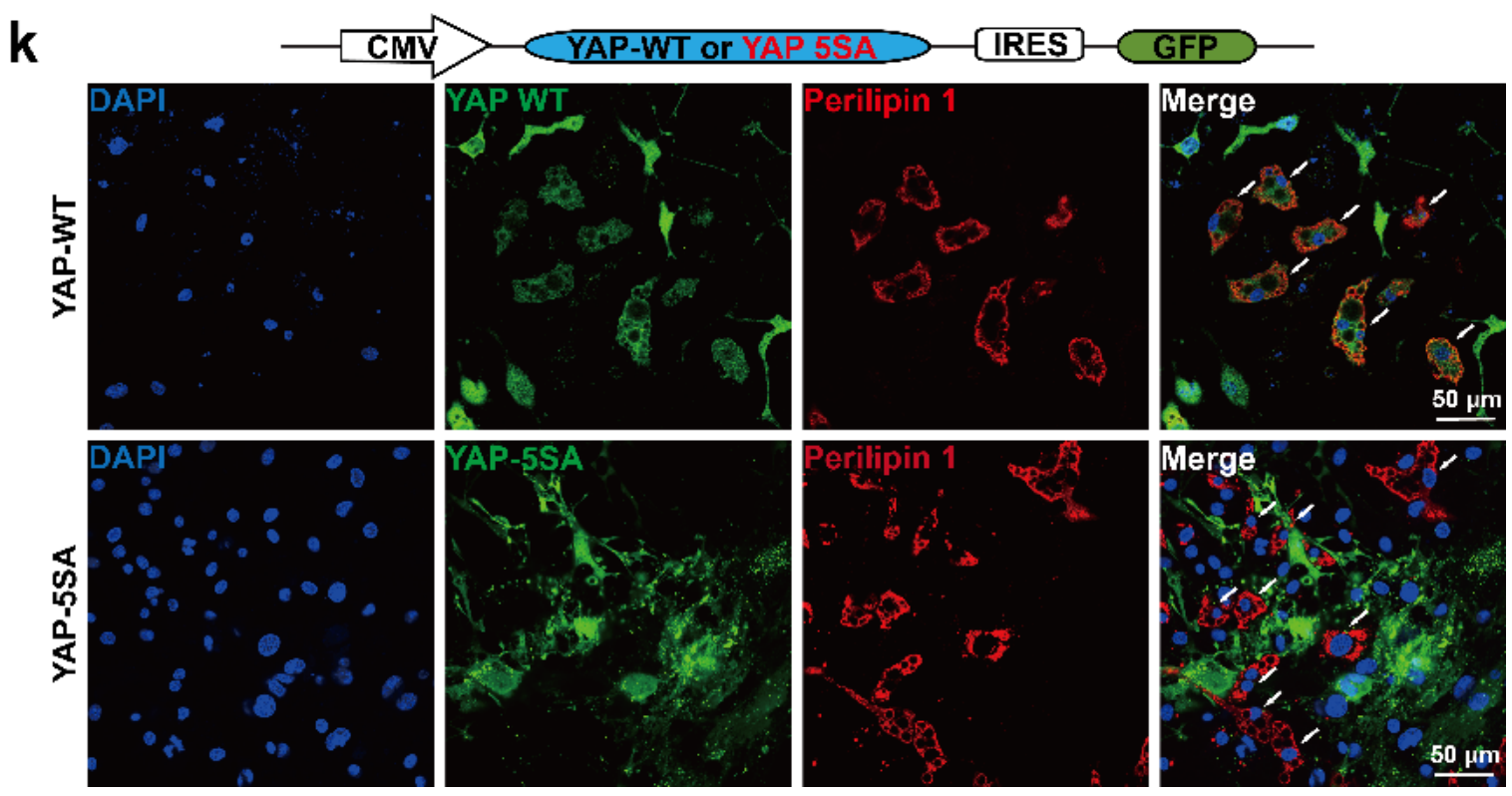

Revised Fig. 6 | … k Representative immunofluorescence images of Ble-treated MEFs overexpressing YAP-WT or YAP-5SA (green), stained for Perilipin 1 (red). Scale bar, 50 μm.

Reviewer #2 (Remarks to the Author):

Brief summary of the manuscript

Li et al. investigate the effect of pharmacological disruption of actomyosin contractility with (−)-Blebbistatin combined with adipogenic cues on ovarian cancer cells. They report that this approach induces adipogenic reprogramming both in vitro and in vivo, linking the observed conversion to chromatin remodeling and YAP nuclear export. The authors propose this as a novel therapeutic concept for ovarian cancer.

Overall impression of the work

The study is conceptually novel and timely, presenting a coherent, multi-layered dataset that integrates mechanistic and in vivo work. However, statistical underpowering, incomplete reporting, and several gaps in experimental validation and translational anchoring reduce confidence in the generalizability and durability of the effect. Addressing the experimental and reporting issues outlined below would substantially strengthen the manuscript.

Specific comments, with recommendations

Major comments

1. Statistical power and transparent reporting

Increase or describe in detail the biological n for key assays, including imaging quantifications (state how many cells and fields of view were analyzed per replicate), RT-qPCR, and in vivo readouts. In every figure legend, specify: exact statistical tests, two-sidedness, multiple-testing correction, alpha, and the definition of replicates. Use adjusted p-values for RNA-seq and targeted assays with multiple comparisons. Overlay individual data points on all plots and provide raw Ct tables (targets and endogenous controls) for RT-qPCR experiments.

Response:

We greatly appreciate your professional suggestions on statistical description and data presentation.

We have thoroughly revised all figure legends to include complete and clear statistical details. We have clearly stated the exact biological replicate numbers for all quantitative assays in each figure legend. Exact statistical methods are clearly noted: two-sided unpaired Student's *t*-test for all

pairwise two-group comparisons, and two-way ANOVA for all proliferation curve analyses. A significance level of $\alpha = 0.05$ is used for all statistical tests. Individual data points are overlaid on all quantitative plots for transparent data display. Raw Ct tables for RT-qPCR assays, including target genes and endogenous controls, have be provided as supplementary material, and adjusted *P*-values are used for all relevant multiple comparisons.

2. RNA-seq pathway validation (GSEA)

Perform GSEA for curated adipogenic differentiation signatures and report NES/q (FDR) to validate pathway-level upregulation beyond DEG lists. Show representative adipogenesis-related transcription factors and downstream effector genes from the RNA-seq analysis.

Response:

We thank you for this insightful and rigorous suggestion.

We have now performed GSEA analysis using curated adipogenic differentiation signatures and presented the corresponding gene-level heatmaps in **Response Fig. 5**.

In both the MEF and SKOV3 model, GSEA revealed consistent positive enrichment of the "**positive regulation of fat cell differentiation**" gene set (NES > 0) **(Response Fig. 5a, c)**, supporting pathway-level activation of the adipogenic program. Although the FDR values did not reach strict statistical significance in MEFs, this is likely due to the moderate fold changes and limited sample size in our bulk RNA-seq data. Key regulators including PPARG, CEBPA, and downstream lipid-metabolism genes were consistently upregulated upon treatment, reinforcing the phenotypic and functional evidence of adipogenic reprogramming **(Response Fig. 5b, d)**.

Together with our molecular and functional validations, these data support the robust activation of adipogenic pathways *in vitro* and *in vivo*. We have noted this statistical limitation in the revised discussion and agree that future studies with larger sample sizes will help further substantiate these pathway-level observations.

Response Fig. 5

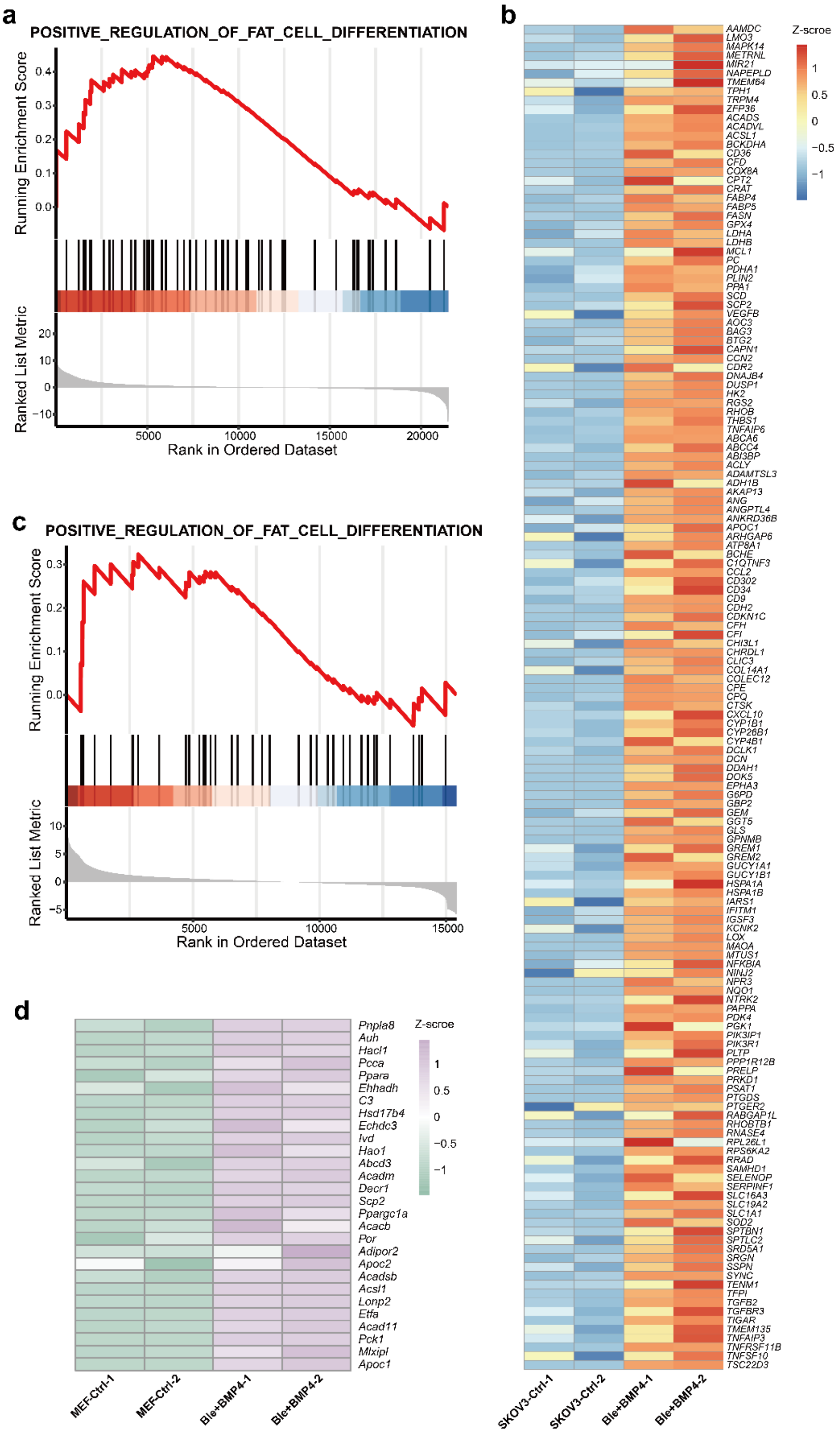

Response Fig. 5 | a GSEA enrichment plot for "positive regulation of fat cell differentiation" in Ble+BMP4-treated SKOV3 cells (NES = 1.5132473, FDR = 0.0782314). **b** Heatmap showing expression of adipogenesis-related transcription factors and downstream effector genes in Ble+BMP4-treated SKOV3 cells. **c** GSEA enrichment plot for "positive regulation of fat cell

differentiation" in Ble+BMP4-treated MEFs (NES = 0.887193, FDR = 0.6496399). **d** Heatmap showing expression of adipogenesis-related transcription factors and downstream effector genes in Ble+BMP4-treated MEFs.

3. MEF controls and prior literature

Reconcile the absence of adipogenesis in MEF controls (Fig. 1b,c) with the well-described multi-lineage potential of MEFs. Provide optimization data or rationale (medium composition, cell confluence, timing, substrate coating, insulin/BMP4 dosing) or include a positive-control protocol demonstrating expected adipogenesis under standard conditions. In addition, including a second cell type with known adipogenic potential—such as mesenchymal stem cells—would help establish whether Blebbistatin generally facilitates adipogenesis.

Response:

We thank you for this thoughtful and rigorous comment, which helps us better contextualize and validate our experimental system.

As extensively documented in the literature, MEFs possess mesenchymal multi-lineage differentiation potential, but this capacity is not spontaneous and strictly requires exogenous pro-adipogenic stimuli **(PMID: 23441817)**. The absence of adipogenesis in the control MEF group (**Original Fig 1b)** is therefore fully consistent with prior studies: control cells were maintained in standard basal growth medium without any differentiation-inducing factors. Notably, BMP4 alone was insufficient to trigger adipogenic conversion in this neutral medium **(Original Fig. S1)**, whereas Ble-mediated cytoskeletal disruption strongly enabled adipogenic transdifferentiation when combined with these cues.

Our experimental conditions were rigorously optimized based on established protocols and preliminary data: **Medium composition**: the medium composition was set as a standard neutral culture environment without directing differentiation toward any specific lineage; **Cell confluence & timing:** cell confluence and induction timing were optimized by continuously monitoring the quantity and size of lipid droplets during culture to ensure optimal differentiation efficiency; **Substrate**: substrate coating included two types, standard cell culture plastic plates induced by Ble, and low-stiffness polydimethylsiloxane (PDMS) coatings utilized to mimic the cytoskeleton

relaxation. **Dosing**: the dosing of BMP4 and other small molecules (Ble, Rosi) was determined following the commonly used and validated concentrations in previous relevant adipogenesis research.

To further validate our system, we performed a positive control experiment using a commercial adipogenic induction kit (Cat# 03.18102C), which confirmed robust lipid accumulation, marker expression, and functional differentiation (**Response Fig. 6**).

In addition, we extended our key findings to adipose-derived mesenchymal stem cells (Adi-MSCs), a canonical cell type with strong, well-defined adipogenic potential. As shown in **Response Fig. 7**, Ble significantly promoted adipogenic differentiation when combined with BMP4 or rosiglitazone, demonstrating that its pro-adipogenic effect is conserved across multiple cell types with adipogenic competence.

These additional controls and cross-cell-type validations strongly support the specificity and generality of our mechanistic findings.

Response Fig. 6

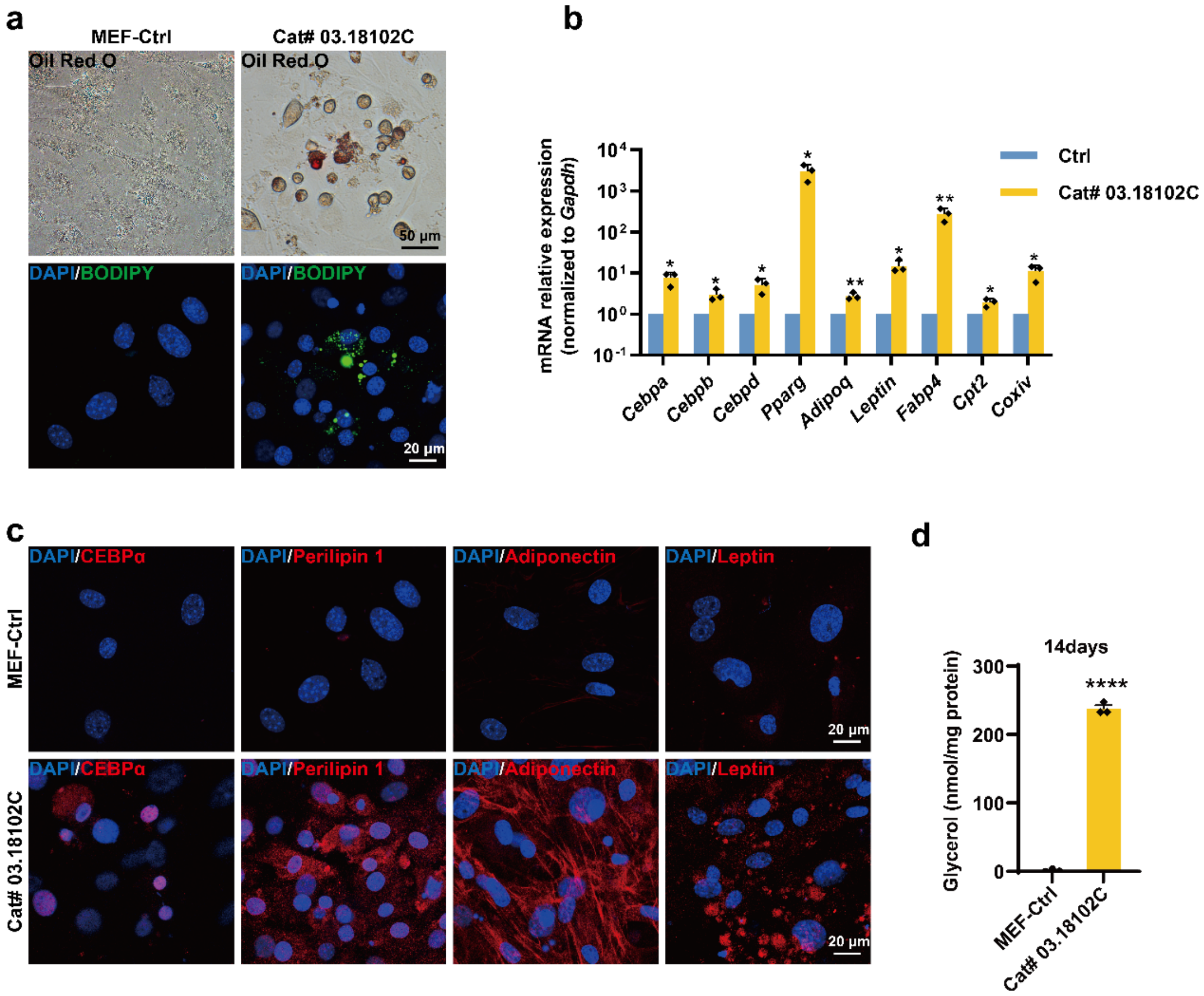

Response Fig. 6 | a Lipid droplets in adipogenic induction differentiation kit (Cat# 03.18102C) treated MEFs at D14 were visualized by Oil Red O and BODIPY staining. Scale bars, 50 μm and 20 μm. **b** qRT–PCR analysis of the mRNA levels of adipocyte-specific genes in Cat# 03.18102C treated MEFs at D14 ($n = 3$). **c** Immunostaining for CEBPα (red), Perilipin 1(red), Adiponectin (red), Leptin (red) and nuclei (DAPI) in Cat# 03.18102C treated MEFs at D14, scale bar, 20 μm. **d** Isoproterenol-induced lipolysis was measured in Cat# 03.18102C treated MEFs at D14 ($n = 3$). Data are presented as mean ± SEM. $*P < 0.05$, $**P < 0.01$, $***P < 0.001$, $****P < 0.0001$. Statistical analyses were performed using two-sided unpaired Student's *t*-test in (**b** and **d**).

Response Fig. 7

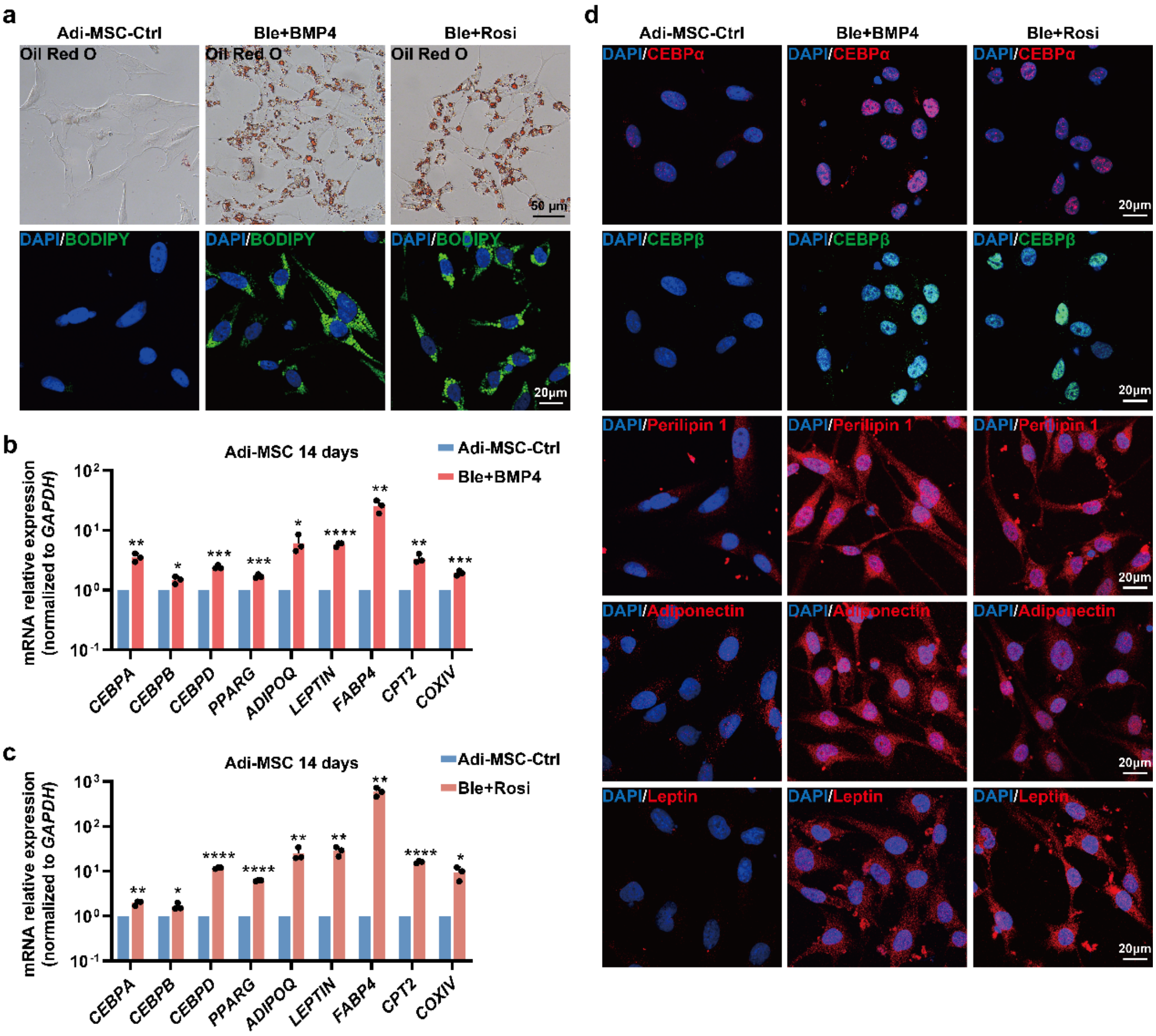

Response Fig. 7 | Lipid droplets in Ble+BMP4/Rosi treated Adi-MSCs at D14 were visualized by Oil Red O and BODIPY staining. Scale bars, 50 μm and 20 μm. **b, c** qRT–PCR analysis of the mRNA levels of adipocyte-specific genes in Ble+BMP4/Rosi treated Adi-MSCs at D14 (n = 3). **d** Immunostaining for CEBPα (red), CEBPβ (green), Perilipin 1(red), Adiponectin (red), Leptin (red) and nuclei (DAPI) in Ble+BMP4/Rosi treated Adi-MSCs at D14, scale bar, 20 μm. Data are presented as mean ± SEM. *P < 0.05, **P < 0.01, ***P < 0.001, ****P < 0.0001. Statistical analyses were performed using two-sided unpaired Student's t-test in (**b**, and **c**).

4. Model breadth and clinical anchoring

Add at least one additional ovarian cancer cell line for all core in vitro and in vivo phenotypes. Ideally, extend to patient-derived organoids or primary tumor cells to increase clinical and translational relevance.

Response:

We greatly appreciate this valuable suggestion to strengthen the model breadth and clinical translational relevance of our study.

In response, we have performed comprehensive validation of all core *in vitro* and *in vivo* phenotypes across three clinically distinct ovarian cancer cell lines: A2780, SKOV3, and OVCAR3. Their key characteristics and adipogenic responses are summarized in **Response Table 1** below.

Response Table 1. Characteristics of ovarian cancer cell lines used and adipogenic transdifferentiation results.

**Cell line**	**Origin**	**Key features**	**Adipogenic transdifferentiation**
A2780	Human ovarian endometrioid adenocarcinoma (primary)	Chemo-sensitive, p53-WT, widely used	Confirmed *in vitro* **(Revised Fig. S3)** and *in vivo* **(Original Fig. 3)**
SKOV3	Human ovarian serous adenocarcinoma (ascites)	Cisplatin-resistant, highly metastatic	Confirmed *in vitro* **(Revised Fig. 2)** and *in vivo* **(Original Fig. 4)**
OVCAR3	Human ovarian serous adenocarcinoma (malignant ascites)	p53-mutated, high-grade serous subtype	Confirmed *in vitro* **(Revised Fig. S4)** and *in vivo* **(Revised Fig. S5)**

In vitro, we verified consistent adipogenic transdifferentiation in all three lines using lipid droplet staining, qRT-PCR/immunofluorescence for adipogenic markers, lipolytic activity assays, and RNA-seq analysis. Transcriptomic profiling confirmed upregulation of adipogenic regulators and downregulation of oncogenic pathways, supported by GO.

In vivo, we extended xenograft modeling to all three cell lines: subcutaneous (A2780) and intraperitoneal models (SKOV3, OVCAR3). In each case, Ble-based treatment induced Perilipin 1-positive adipogenic conversion and reduced tumor burden.

Our panel of three well-established ovarian cancer lines covers major clinical subtypes: chemosensitive, drug-resistant, and p53-mutated high-grade serous. Their consistent response to Ble-induced reprogramming strongly supports the robustness, generality, and translational potential of our findings.

Revised Fig. S3

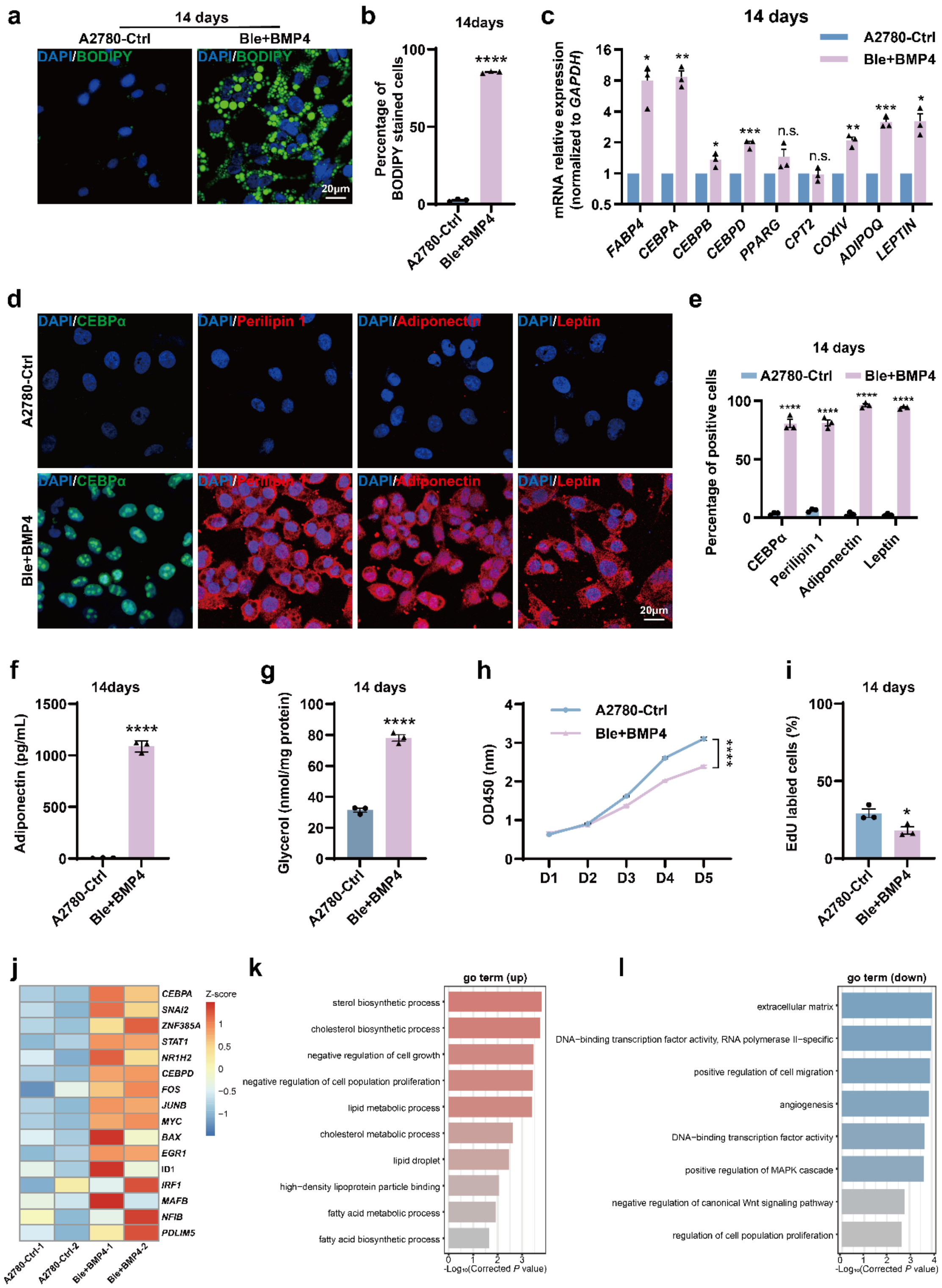

Revised Fig. S3 | Ble induces A2780 cells transdifferentiation into adipocyte-like cells and inhibits cell proliferation. a Lipid droplets in Ble+BMP4 treated A2780 cells at D14 were visualized by BODIPY staining. Scale bars, 20 μm. **b** The percentage of BODIPY-stained cells was quantified (n = 3). **c** qRT–PCR analysis of the mRNA levels of adipocyte-specific genes in

Ble+BMP4 treated A2780 cells at D14 (n = 3). **d** Immunostaining for CEBPα (green), Perilipin 1(red), Adiponectin (red), Leptin (red) and nuclei (DAPI) in Ble+BMP4 treated A2780 cells at D14; scale bar, 20 μm. **e** CEBPα-, Perilipin 1-, Adiponectin- and Leptin- positive cells were quantified (n = 3). **f** Adiponectin secretion levels in Ble+BMP4 treated A2780 cells at D14 were measured by ELISA (n = 3). **g** Isoproterenol-induced lipolysis was measured in Ble+BMP4 treated A2780 cells at D14 (n = 3). **h** CCK-8 analysis of Ble+BMP4 treated A2780 cells revealed a lower cell proliferation rate (n = 3). **i** Ble+BMP4 treated A2780 cells were incubated with 5-ethynyl-2'-deoxyuridine (EdU) for 2.5 hr to label proliferating cells. EdU-labeled cells were quantified using ImageJ (n = 3). **j** Heatmap showing differential expression of adipogenesis-related transcription factors in A2780 cells with or without Ble+BMP4 treatment. **k, l** Bar plot of selected functional enrichment terms of genes upregulated and downregulated in Ble+BMP4 treated A2780 cells. Random visual fields were selected from each group to avoid artificial selection bias for panel **b, e, i.** Data are presented as mean ± SEM. *$P < 0.05$, **$P < 0.01$, ***$P < 0.001$, ****$P < 0.0001$. n.s., no significant difference ($P > 0.05$). Statistical analyses were performed using two-sided unpaired Student's t-test in (**b**, **c**, **e-g**, **i**) and two-way ANOVA in (**h**).

Revised Fig. S4

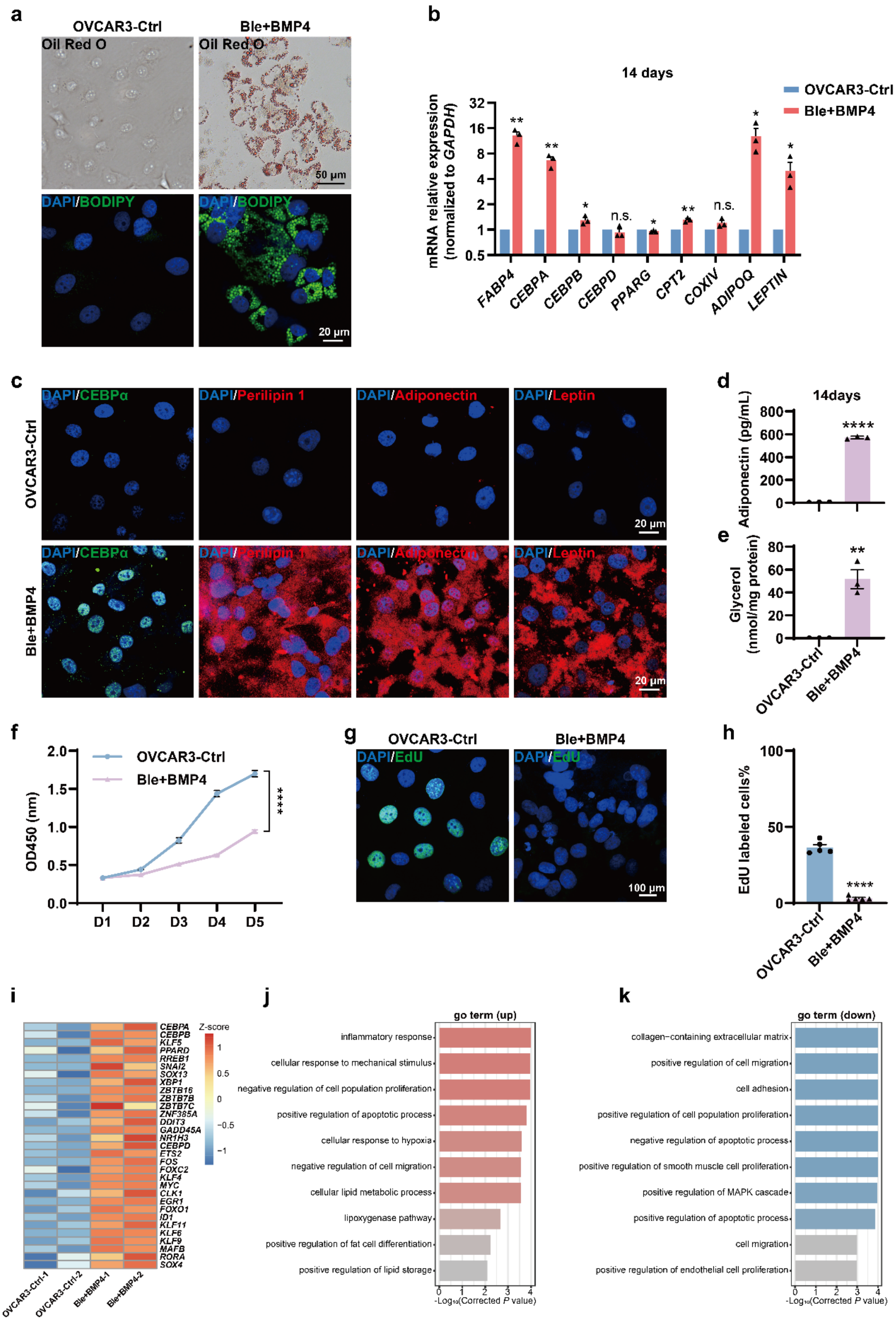

Revised Fig S4 | Ble induces OVCAR3 cells transdifferentiation into adipocyte-like cells and inhibits cell proliferation. a Lipid droplets in Ble+BMP4 treated OVCAR3 cells at D14 were

visualized by Oil Red O and BODIPY staining. Scale bars, 50 µm and 20 µm. **b** qRT–PCR analysis of the mRNA levels of adipocyte-specific genes in Ble+BMP4 treated OVCAR3 cells at D14 (*n* = 3). **c** Immunostaining for CEBPα (green), Perilipin 1(red), Adiponectin (red), Leptin (red) and nuclei (DAPI) in Ble+BMP4 treated OVCAR3 cells at D14; scale bar, 20 µm. **d** Adiponectin secretion levels in Ble+BMP4 treated OVCAR3 cells at D14 were measured by ELISA (*n* = 3). **e** Isoproterenol-induced lipolysis was measured in Ble+BMP4 treated OVCAR3 cells at D14 (*n* = 3). **f** CCK-8 analysis of Ble+BMP4 treated OVCAR3 cells revealed a lower cell proliferation rate (*n* = 3). **g, h** Ble+BMP4 treated OVCAR3 cells were incubated with 5-ethynyl-2'-deoxyuridine (EdU) for 2.5 hr to label proliferating cells. EdU-labeled cells were quantified using ImageJ (*n* = 3), scale bar, 100 µm. **i** Heatmap showing differential expression of adipogenesis-related transcription factors in OVCAR3 cells with or without Ble+BMP4 treatment. **j, k** Bar plot of selected functional enrichment terms of genes upregulated and downregulated in Ble+BMP4 treated OVCAR3 cells. Random visual fields were selected from each group to avoid artificial selection bias for panel **h.** Data are presented as mean ± SEM. *$P < 0.05$, **$P < 0.01$, ***$P < 0.001$, ****$P < 0.0001$. n.s., no significant difference ($P > 0.05$). Statistical analyses were performed using two-sided unpaired Student's *t*-test in (**b**, **d**, **e**, **h**) and two-way ANOVA in (**f**).

Revised Fig. S6

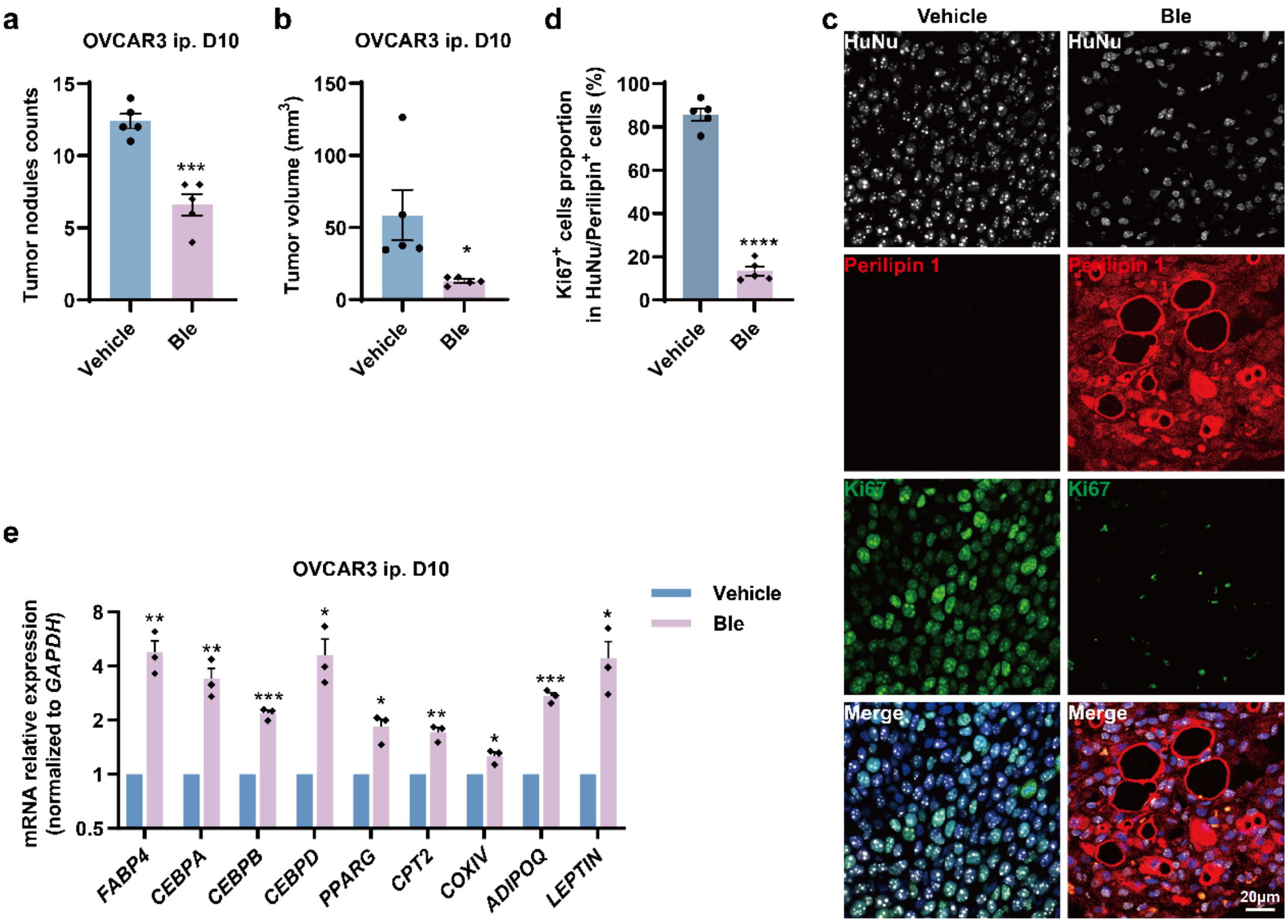

Revised Fig. S6 | Ble alone treatment inhibits OVCAR3 tumor growth in a mouse model of ovarian cancer intraperitoneal metastasis. a Tumor nodules counts are shown after 10 days of treatment (n = 5). **b** Tumor weights are shown after 10 days of treatment (n = 5). **c** Representative images of tumor sections that were stained with HuNu (white), Ki67 (green), and Perilipin 1 (red). Scale bars, 20 μm. **d** The Ki67-positive cells fraction in HuNu and Perilipin 1 co-positive cells was quantified (n = 5). **e** qRT–PCR analysis of the mRNA levels of adipocyte-specific genes in abdominal tumors from Ble treated mice (n = 3). Random visual fields were selected from each group to avoid artificial selection bias for panel **d.** Data are presented as mean ± SEM. *P < 0.05, **P < 0.01, ***P < 0.001, ****P < 0.001. Statistical analyses were performed using two-sided unpaired Student's t-test in (**a**, **b**, **d**, **e**).

5. Single-cell resolution of reprogramming (optional but highly valuable)

Incorporate scRNA-seq and/or scATAC-seq to define the fraction of cells undergoing conversion, characterize heterogeneity, and directly link chromatin accessibility to adipogenic transcription factor activity. This would strengthen mechanistic insight and help anticipate tumor

microenvironment influences, including the risk of cancer-associated adipocyte (CAA) emergence.

Response:

We sincerely appreciate your insightful suggestion to incorporate single-cell RNA-seq and/or scATAC-seq for dissecting cellular heterogeneity, mapping adipogenic transdifferentiation frequencies, and linking chromatin accessibility to adipogenic transcription factor activity. This approach would indeed deepen our mechanistic understanding and help address the critical question of cancer-associated adipocyte (CAA) emergence in the tumor microenvironment (TME).

Consistent with your observation, solid ovarian tumors exhibit inherent spatial and cellular heterogeneity, which may contribute to variable therapeutic responses across the tumor mass. To address this, we performed bulk RNA-sequencing on in vivo tumor tissues with robust adipogenic transdifferentiation **(Original Figure 3)**. While bulk profiling reflects aggregate transcriptional changes, our data, combined with rigorous validation across three clinically distinct ovarian cancer cell lines (A2780, SKOV3, OVCAR3), fully supports our core conclusions: Ble induces consistent adipogenic transdifferentiation and suppresses tumor proliferation.

Given the substantial costs and TME-related challenges of high-throughput single-cell sequencing, we have prioritized strengthening the current study with existing, well-powered datasets rather than adding unplanned single-cell assays. We have revised the Discussion section to explicitly acknowledge tumor heterogeneity, highlight the value of single-cell technologies for resolving subpopulation dynamics and CAA formation, and note that single-cell analyses will be a high-priority direction for our future mechanistic studies.

Revised Discussion

"…Although we have validated the anti-tumor efficacy of myosin II inhibition-mediated adipogenic transdifferentiation in subcutaneous and intraperitoneal ovarian cancer xenograft models, several limitations should be noted.…In addition, bulk RNA-seq provides only population-level insights and cannot resolve tumor cell subpopulation dynamics or cellular interactions within the tumor microenvironment; future single-cell RNA sequencing will be required to dissect the heterogeneous mechanisms underlying adipogenic transdifferentiation. …Of note, while the adipocyte-like cells generated from tumor cell transdifferentiation exhibited a stable, quiescent phenotype without evidence of pathological characteristics associated with cancer-associated adipocytes (CAAs), their

long-term in vivo fate and potential to gradually acquire CAA-related tumor-promoting properties require further long-term observation and characterization; …"

6. In vivo efficacy endpoints

Extend beyond short-term tumor volume measurements by including survival and recurrence analyses (e.g., Kaplan–Meier) and longer follow-up. This is critical, as novel therapies often show strong early effects that later diminish due to resistance mechanisms.

Response:

We greatly appreciate this critical and insightful suggestion to extend efficacy evaluation beyond short-term tumor volume, emphasizing long-term survival and recurrence endpoints—an important consideration, as novel therapies often elicit robust initial responses that wane over time due to acquired resistance.

For subcutaneous xenografts, animal ethics mandates termination by Day 14, as control tumors reached the maximum allowable volume by this time. Given heterogeneous intratumoral/peritumoral drug distribution in vivo, we posit that residual tumor cells with incomplete Ble exposure would eventually proliferate and drive recurrence—an outcome not seen *in vitro*, where uniform drug exposure eliminates such escapees.

To assess long-term efficacy, we extended intraperitoneal (i.p.) xenograft observations to Day 45 (supplementary experiments). This endpoint was selected because control mice developed severe cachexia or succumbed to tumor burden before Day 45. Serial in vivo bioluminescence imaging (every 7 days through Day 35) revealed persistently low tumor burden in Ble-treated mice, whereas controls exhibited a transient adaptive regression followed by aggressive regrowth, leading to cachexia and mortality **(Response Fig. 8)**.

Regarding resistance risk: this transdifferentiation strategy differs fundamentally from conventional chemotherapy. Unlike DNA-damaging or antimitotic agents—prone to resistance via mutations, target loss, or drug efflux—Ble acts by remodeling chromatin accessibility. Ovarian cancer's inherent lipophilic tropism drives preferential metastasis to adipose-rich sites (peritoneum, omentum); we leverage this native adipose microenvironment to induce adipogenic

transdifferentiation. This non-cytotoxic, reprogramming-based mechanism imposes no selective pressure for resistant clones, thereby bypassing classic chemoresistance pathways entirely.

The main *in vivo* limitation we observed is inconsistent drug distribution and incomplete tumor coverage, causing inter-individual response variability. This delivery barrier drives experimental heterogeneity and represents a key translational challenge. We have expanded the Discussion to address this limitation, including considerations for patient selection and regimen optimization, to guide future clinical development.

Response Fig. 8

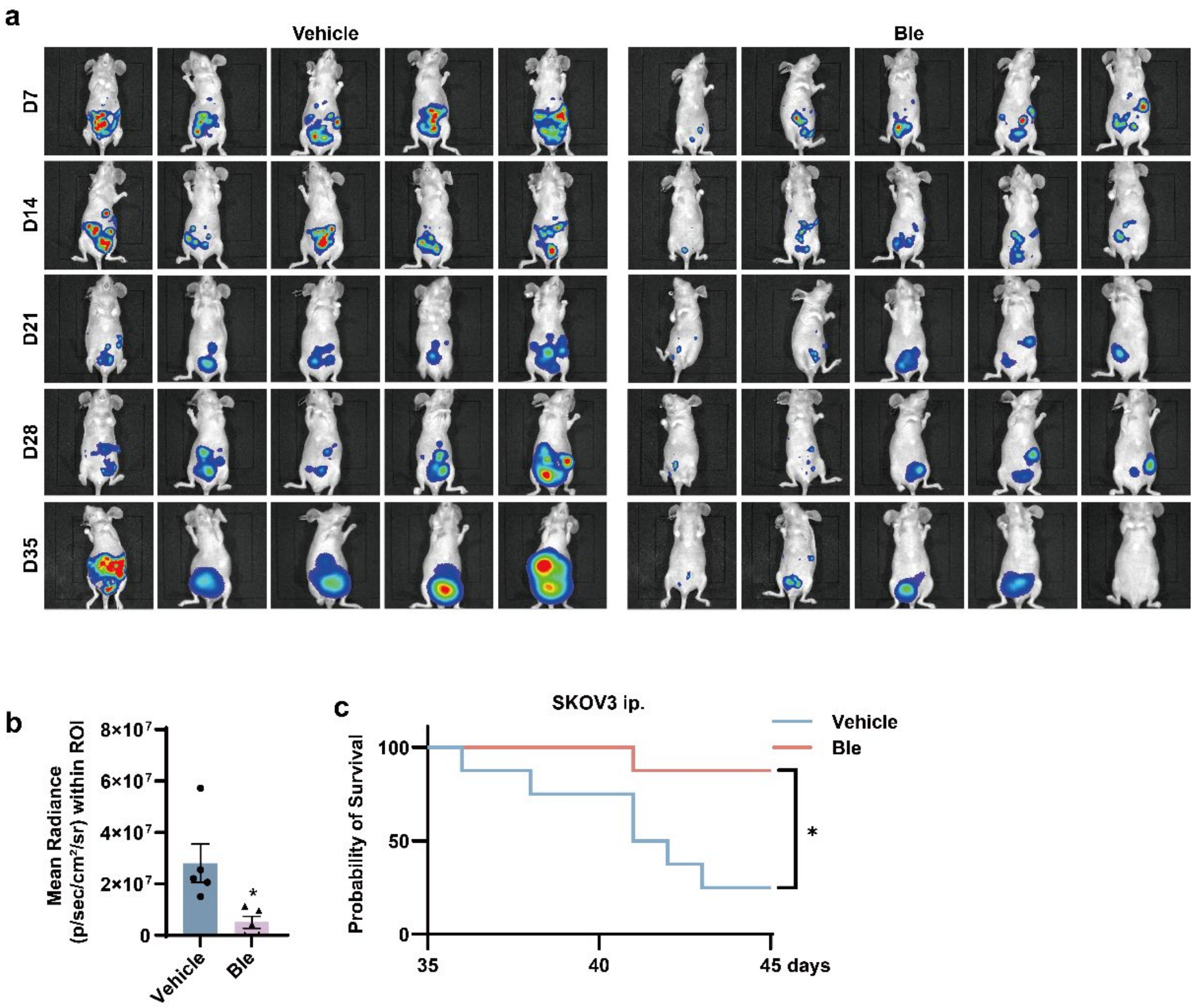

Response Fig. 8 | a Representative bioluminescence images of vehicle and Ble treatment mice at D7, D14, D21, D28, and D35 after Ble administration. **b** Quantification of tumor radiance (n = 5) at D35. **c** Survival curve of SKOV3 tumor-bearing mice after intraperitoneal administration of Ble alone (n = 8). Data are presented as mean ± SEM. *$P < 0.05$. Statistical analyses were performed using two-sided unpaired Student's t-test in (**b**) and Log-rank (Mantel-Cox) test in (**c**).

Revised Discussion

"Although we have validated the anti-tumor efficacy of myosin II inhibition-mediated adipogenic transdifferentiation in subcutaneous and intraperitoneal ovarian cancer xenograft models, several limitations should be noted. …Additionally, the long-term stability of this transdifferentiation strategy has not been fully evaluated, and whether the adipocyte phenotype can be stably maintained or may undergo phenotypic reversal still requires further validation using lineage-tracing approaches. …"

7. Safety/off-target profiling

Provide basic toxicology and off-target assessments for myosin II inhibition in non-tumor tissues (histopathology, serum chemistries, body weight) and discuss dosing/pharmacokinetic considerations, given Blebbistatin's known cytotoxicity, photoinstability, and poor solubility.

Response:

We greatly appreciate this critical assessment of the safety, off-target effects, dosing, and pharmacokinetic (PK) considerations for myosin II inhibition, particularly given Ble's well-documented limitations: cytotoxicity, photoinstability, and poor solubility.

To address these concerns, we performed a 14-day systemic safety evaluation in healthy mice receiving daily intraperitoneal (i.p.) Ble administration. We monitored body weight throughout the study and assessed serum biochemical parameters and H&E histopathology of major organs at the endpoint. No significant body weight loss, abnormal serum chemistry, or histopathological damage to vital organs was observed in the Ble-treated group **(Response Fig. 9)**.

We selected a moderate dose of 3 mg/kg for i.p. injection. A published study **(PMID: 33468641)** employed a comparable dosage (3.6 mg/kg) in rats and reported detailed toxicological and PK data, including a short metabolic half-life (~20.2 min) of Ble in primary hepatocytes. This rapid turnover supports our once-daily dosing strategy, minimizing drug accumulation and systemic toxicity, and validates the safety of our *in vivo* treatment regimen.

Recognizing Ble's inherent drawbacks—including cardiac safety risks and poor druggability—we sought a safer, more translatable myosin II inhibitor to advance our adipogenic transdifferentiation strategy. We identified MT228, an optimized non-muscle myosin II (NMII) inhibitor with 17-fold

selectivity for NMII over cardiac myosin II (CMII), superior biosafety, and improved physicochemical properties. MT228 retains potent NMII inhibition while markedly reducing CMII activity, thereby mitigating the cardiac toxicity associated with Ble. Additionally, MT228 exhibits enhanced solubility (42 μM *vs* 21 μM for Ble) and photostability (93% activity retained after 24 h light exposure), alongside favorable PK profiles **(PMID: 40602401)**. Functionally, we validated in vitro that MT228 + BMP4 efficiently induces adipogenic transdifferentiation in MEFs, with robust lipid droplet formation, upregulated adipogenic gene expression, and positive marker staining after 14 days **(Response Fig. 10)**.

Collectively, these safety data, PK analyses, and alternative compound validation comprehensively address your concerns regarding toxicity, off-target effects, and dosing/pharmacokinetic considerations.

Response Fig. 9

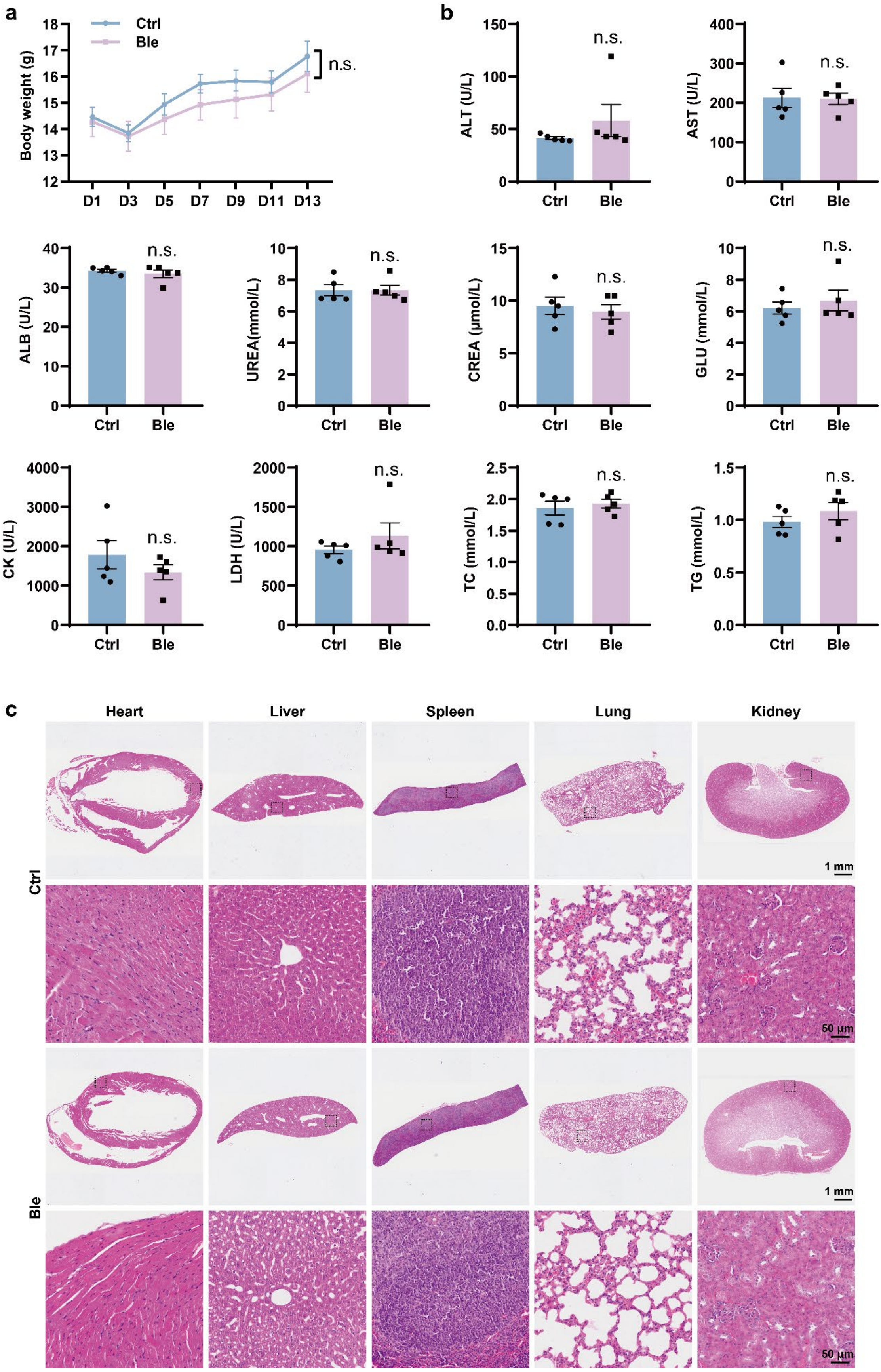

Response Fig. 9 | a Body weight growth curves are shown during the treatment time course ($n = 5$ for each group) **b** Detection of serum biochemical indices related to liver function, renal function, blood glucose, blood lipid and myocardial injury in Ble-treated mice. **c** Representative histopathological morphology of crucial visceral organs (heart, liver, spleen, lung, kidney) in mice following Ble treatment, with integral field and partial enlarged microscopic observations. Data are presented as mean ± SEM. n.s. $P > 0.05$. Statistical analyses were performed using two-sided unpaired Student's *t*-test in (**b**) and two-way ANOVA in (**a**).

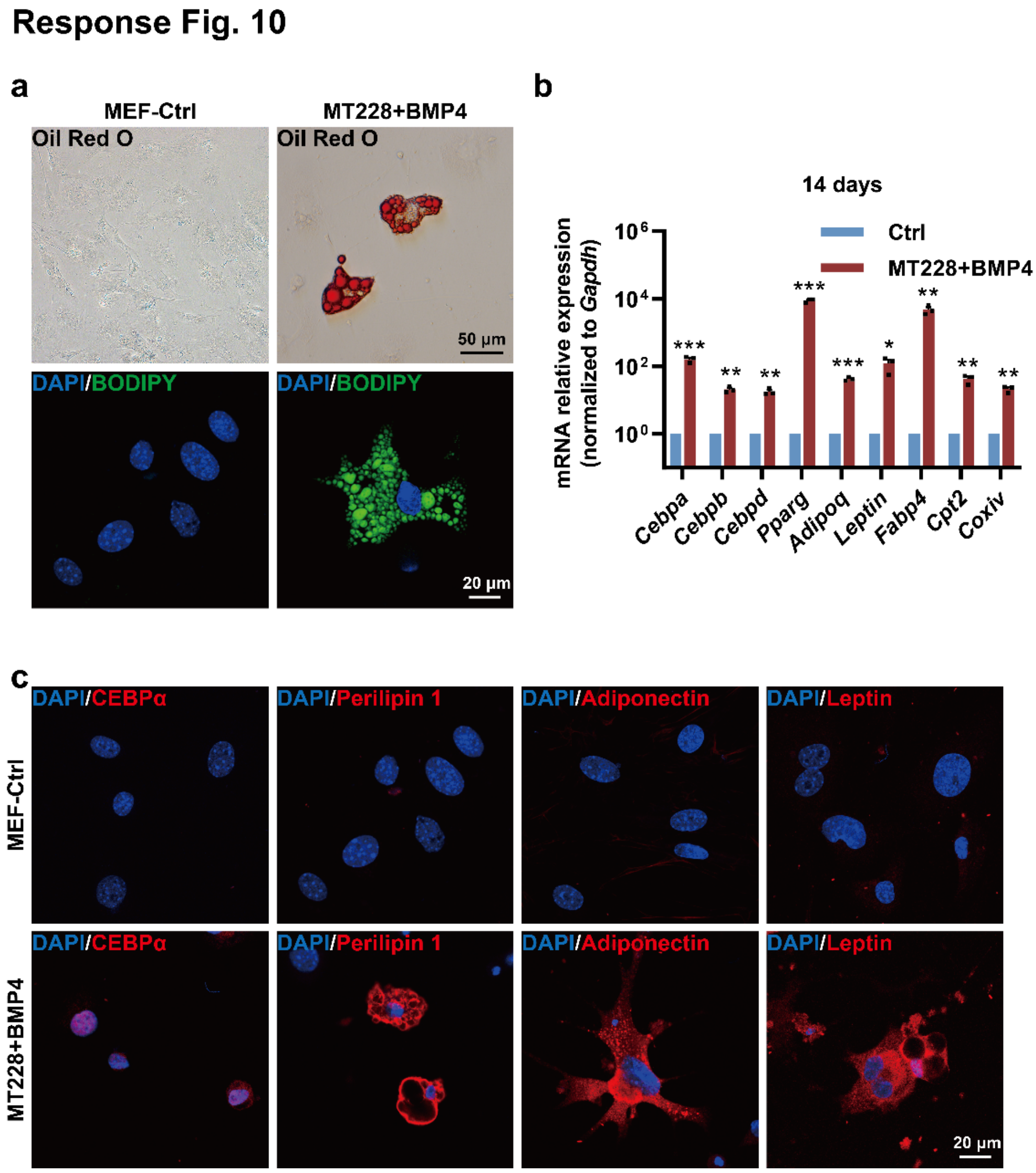

Response Fig. 10 | a Lipid droplets in MT228+BMP4 treated MEFs at D14 were visualized by Oil Red O and BODIPY staining. Scale bars, 50 μm and 20 μm. **b** qRT–PCR analysis of the mRNA levels of adipocyte-specific genes in MT228+BMP4 treated MEFs at D14 ($n = 3$). **c** Immunostaining for CEBPα (red), Perilipin 1(red), Adiponectin (red), Leptin (red) and nuclei (DAPI) in MT228+BMP4 treated MEFs at D14; scale bar, 20 μm. Data are presented as mean ± SEM. $*P <$

0.05, $^{**}P < 0.01$, $^{***}P < 0.001$, $^{****}P < 0.0001$. Statistical analyses were performed using two-sided unpaired Student's *t*-test in (**b**).

8. Durability of adipogenic conversion and CAA risk

Assess the stability of the induced adipogenic state after drug withdrawal (e.g., lineage tracing or washout experiments). Evaluate whether converted cells could become CAAs, which are known to support metastasis and therapy resistance, and discuss these implications for relapse risk.

Response：

Thank you for your query on adipogenic conversion durability and CAA risk.

To address the stability of the induced adipogenic state, we performed an *in vitro* drug washout experiment using A2780 cells. Following successful adipogenic transdifferentiation, Ble was removed, and cells were cultured for an additional 7 days. qRT–PCR analysis of core adipogenic genes (e.g., *PPARG*, *CEBPA*) and immunofluorescence staining for Perilipin 1 confirmed stable retention of the adipogenic phenotype without evidence of de-differentiation **(Response Fig. 11)**. This finding demonstrates that the adipogenic state induced by Ble is stably maintained in the absence of continuous drug exposure.

Consistently, our *in vivo* intraperitoneal xenograft model further validated this durability: within 3 weeks of drug cessation, Ble-treated tumors remained suppressed with no obvious rebound in tumor burden **(Response Fig. 8)**, indicating a low risk of early tumor relapse driven by reversion to a malignant phenotype.

Regarding the potential risk of converted cells transitioning into pro-metastatic and therapy-resistant CAAs, we note that classic CAAs are defined by pro-inflammatory, pro-tumorigenic secretory profiles (e.g., elevated IL-6, TNF-α, adipokines) and reduced lipid storage, which collectively promote metastasis and therapy resistance **(PMID: 36551185)**. In contrast, our Ble-converted cells exhibit mature adipocyte features: low proliferative activity, terminal differentiation, and a lipid-storing phenotype with no evidence of the pro-tumorigenic secretory repertoire or malignant functional properties characteristic of CAAs.

We have expanded the Discussion to explicitly address these points, including the low CAA risk and durable adipogenic state. Future long-term in vivo studies will focus on dissecting CAA-related properties and further characterizing the long-term fate of converted cells.

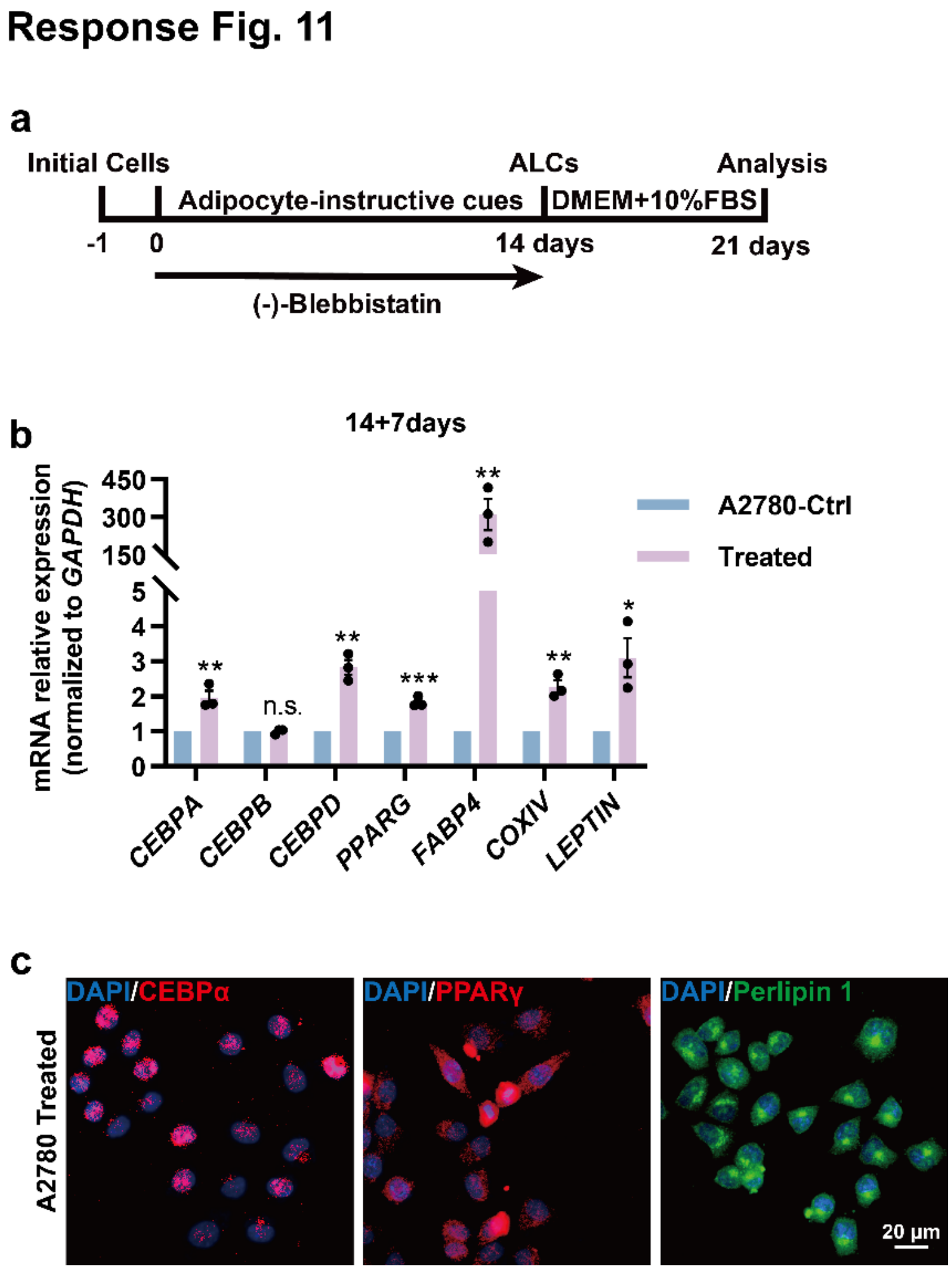

Response Fig. 11 | a. Schematic of the *in vitro* washout experiment showing the stability of adipogenic transdifferentiation in A2780 cells after withdrawal of Ble+BMP4**. b** qRT–PCR analysis of the mRNA levels of adipocyte-specific genes in Ble withdrawal converted A2780 cells ($n = 3$). **c** Immunostaining for CEBPα (red), PPARγ (red), Perilipin 1 (red), and nuclei (DAPI) in Ble withdrawal converted A2780 cells; scale bar, 20 μm. Data are presented as mean ± SEM. *$P < 0.05$, **$P < 0.01$, ***$P < 0.001$, ****$P < 0.0001$. Statistical analyses were performed using two-sided unpaired Student's *t*-test in (**b**).

Revised Discussion

"Although we have validated the anti-tumor efficacy of myosin II inhibition-mediated adipogenic transdifferentiation in subcutaneous and intraperitoneal ovarian cancer xenograft models, several

limitations should be noted. … Of note, while the adipocyte-like cells generated from tumor cell transdifferentiation exhibited a stable, quiescent phenotype without evidence of pathological characteristics associated with cancer-associated adipocytes (CAAs), their long-term in vivo fate and potential to gradually acquire CAA-related tumor-promoting properties require further long-term observation and characterization; additionally, the long-term stability of this transdifferentiation strategy has not been fully evaluated, and whether the adipocyte phenotype can be stably maintained or may undergo phenotypic reversal still requires further validation using lineage-tracing approaches."

Minor comments

1. Imaging and consistency

Provide higher-magnification BODIPY images to evaluate lipid droplet size and maturity. Improve resolution in Fig. 2 and ensure representative fields match the quantified percentages. State whether image selection and thresholding were blinded.

Response:

We greatly appreciate these valuable suggestions to enhance the clarity, consistency, and rigor of our imaging data.

To better visualize lipid droplet size and maturity, we have supplemented higher-magnification BODIPY images of MEF, A2780, SKOV3, and OVCAR3 cells treated with Ble+BMP4, clearly showing the morphology, size and maturity of lipid droplets **(Response Fig. 12)**. Meanwhile, we have improved the image resolution of **Original Fig. 2** and re-quantified the positive cell percentages using representative fields, ensuring consistency between microscopic images and statistical quantification.

For image acquisition and analysis, random visual fields were selected from each group to avoid artificial selection bias. All images were captured under identical microscopic parameters, and a unified analysis threshold was applied for positive cell calculation. Although blinding was not performed in this experiment, random field selection and standardized unified analytical criteria ensure the objectivity and reliability of our results. We have now **added these details to the Materials and Methods section of the revised manuscript**.

Response Fig. 12

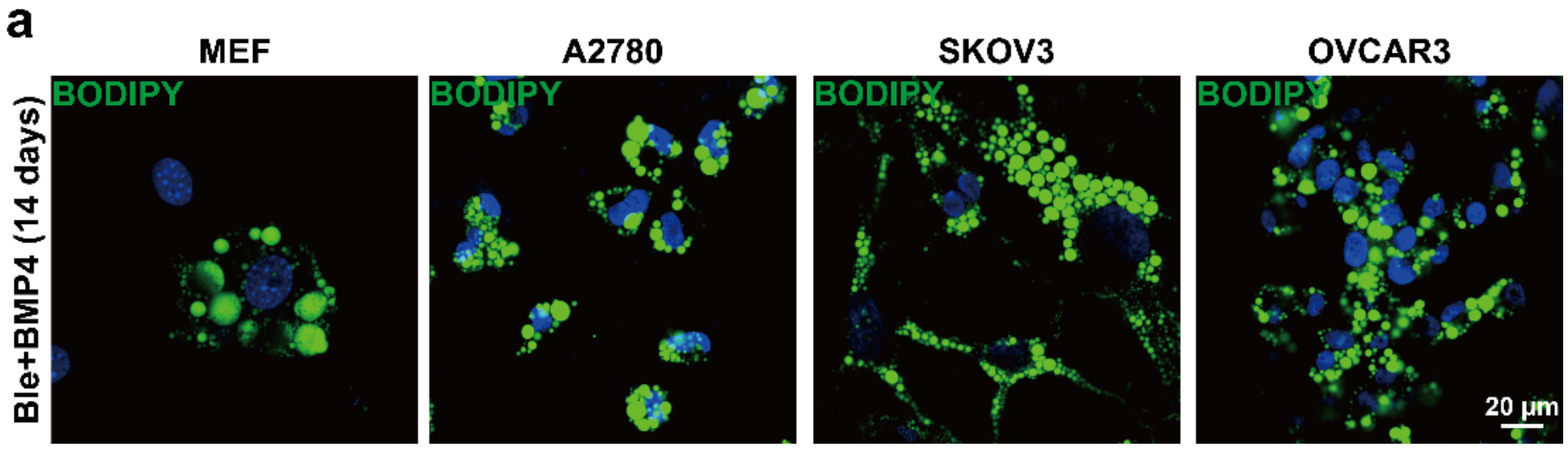

Response Fig. 12 | a Lipid droplets in Ble+BMP4 treated MEF, A2780, SKOV3, OVCAR3 at D14 were visualized by BODIPY staining. Scale bars, 20 μm.

2. Heatmaps and units (Fig. 1f)

Replace "max/min" alone with a clear numeric scale (e.g., log2FC or z-score) and specify measurement units and data transformation.

Response:

Thank you for the comment on the heatmap scale and units.

We have replaced the vague "max/min" display scale with a clear numerical scale (Z-Score) in the revised figures. Meanwhile, we have explicitly annotated the original measurement units of each dataset and fully described all data transformation and normalization methods in the figure legends and Methods section.

Revised Fig. 1

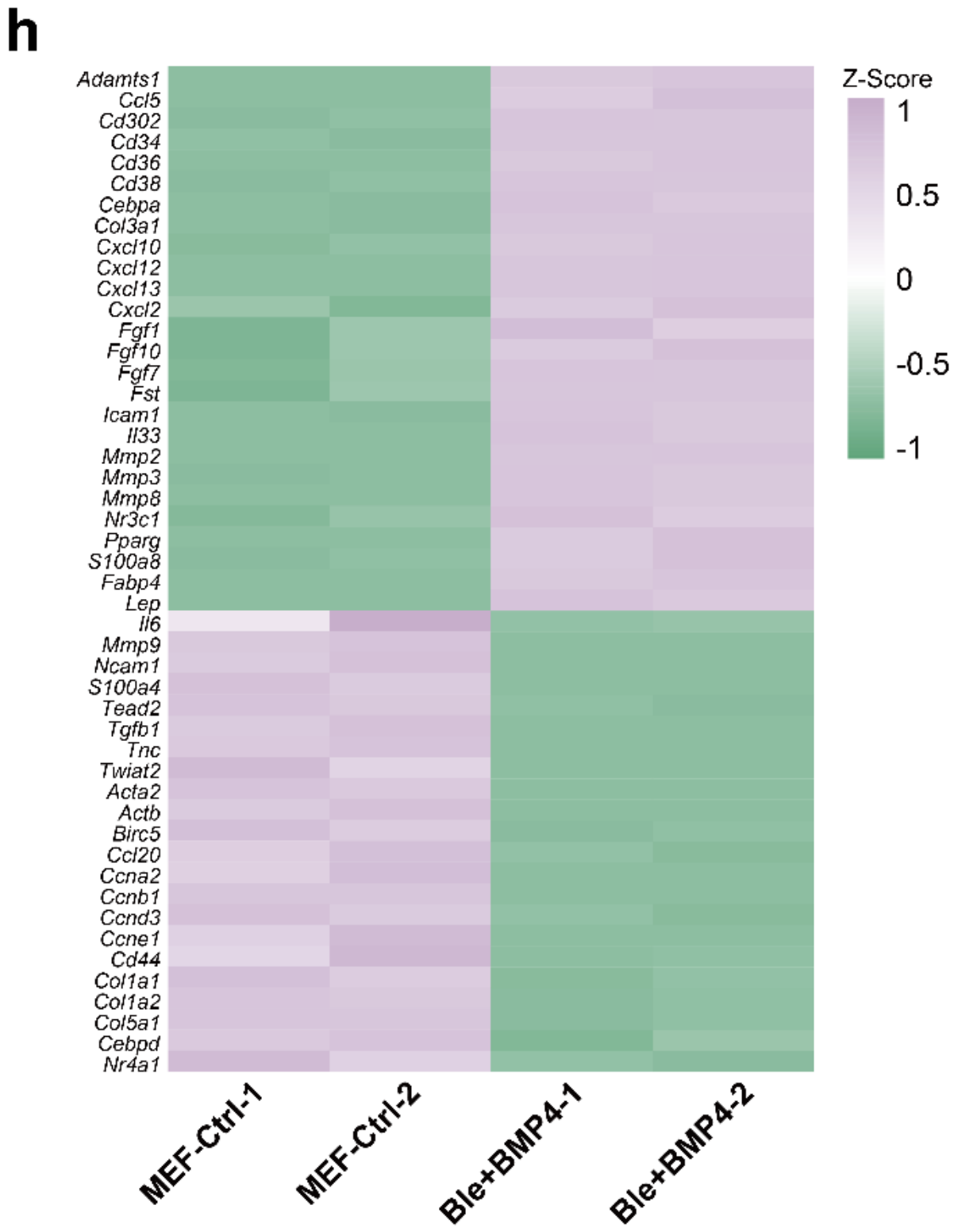

Revised Fig. 1 | … **h** Heatmap of selected adipocyte-specific and MEF-specific gene expression in Ble+BMP4 treated MEFs.

3. Figure and legend specificity

For each panel, specify the assay platform (RNA-seq vs RT-qPCR), the exact cell type used (e.g., Fig. 6), and the number/type of replicates. Define all abbreviations and clarify what "max/min" denote.

Response:

We appreciate this constructive suggestion to enhance the clarity, completeness, and reproducibility of our figure legends.

We have carefully revised all figure legends in the revised manuscript. We clearly specified the experimental detection platform for each panel, annotated the exact cell types applied in every figure with detailed clarification for **Revised Fig. 6**, and explicitly stated the number and type of biological or technical replicates for each experiment. In addition, we defined all abbreviations at their first appearance throughout the text, and the previously vague "max/min" display in heatmaps has been

replaced with a clear numerical scale (Z-Score), with relevant explanations supplemented in the figure legends.

Revised Fig. 6

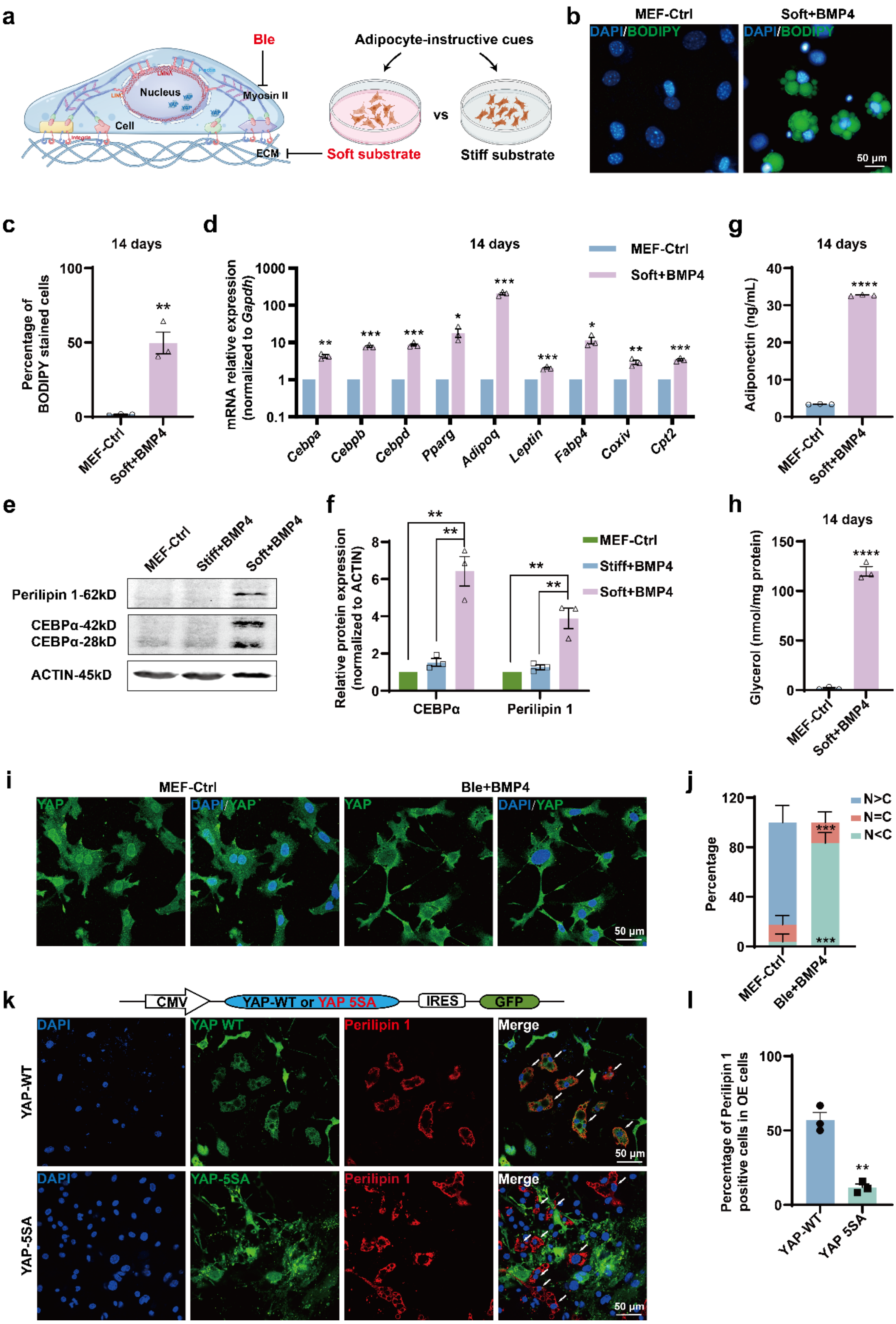

Revised Fig. 6 | Mechanical signals promote adipogenic transdifferentiation. a Schematic of adipogenic transdifferentiation induced by soft substrate culture combined with adipocyte-instructive cues (BMP4) treatment. **b** Lipid droplets of MEFs cultured on stiff (Ctrl) versus soft substrates with BMP4 for 14 days were visualized by BODIPY staining. Scale bars, 50 µm. **c** The percentage of BODIPY-stained cells was quantified (n = 3). **d** qRT–PCR analysis of the mRNA levels of adipogenesis- and FAO-related genes in MEFs cultured on soft substrates with BMP4 (n = 3). **e** Western blot analysis of CEBPα and Perilipin 1 in MEFs cultured on soft substrates with BMP4 for 14 days. **f** Quantitative analysis of **g,** the relative protein expression of CEBPα and Perilipin 1 (n = 3). **g** Adiponectin secretion levels were measured for MEFs cultured on soft substrates with BMP4 by ELISA (n = 3). **h** Isoproterenol-induced lipolysis was measured in MEFs cultured on soft substrates with BMP4 for 14 days (n = 3). **i** Immunocytochemistry of YAP subcellular localization in Ble+BMP4 treated cells. Scale bar, 50 µm. **j** YAP subcellular localization percentage was quantified (n = 3). N, Nucleus; C, Cytoplasm. **k** Representative immunofluorescence images of Ble-treated MEFs overexpressing YAP-WT or YAP-5SA (green), stained for Perilipin 1 (red). Scale bar, 50 µm. **l** Quantification of the percentage of Perilipin 1-positive cells in MEFs overexpressing YAP-WT or YAP-5SA (n = 3). Data are presented as mean ± SEM. *$P < 0.05$, **$P < 0.01$, ***$P < 0.001$, ****$P < 0.0001$. Statistical analyses were performed using two-sided unpaired Student's t-test in (**c-h**, **j**, and **l**).

4. qPCR data presentation (Figs. 2c, 3d; Suppl. Fig. 3SC)

Overlay individual data points; provide raw Ct tables; correct missing SD values (e.g., FABP4 in Suppl. Fig. 3SC). Investigate and explain unexpected zero SD in cancer-cell differentiation experiments.

Response:

Thank you for the qPCR data presentation suggestions.

We have overlaid individual data points on all statistical graphs and provided raw Ct tables for RT-qPCR detection in the supplementary files. We carefully checked and supplemented the missing SD values (e.g., FABP4 in **Original Fig. S3c**), Meanwhile, we have confirmed there are no abnormal

zero SD values in cancer-cell differentiation experiments, and all statistical variations are derived from reliable independent biological replicates.

5. Terminology and scope of conclusions

Calibrate claims in the Abstract and Discussion to reflect the current preclinical status. Explicitly acknowledge limitations regarding model scope, conversion durability, and safety.

Response:

Thanks for your suggestion.

We have carefully moderated the conclusions described in the Abstract and Discussion to fully reflect the preclinical stage of the present study. Meanwhile, we have explicitly acknowledged the key limitations of this work in the revised Discussion, including the restricted tumor model scope, the durability of *in vivo* adipogenic conversion, and the systemic safety and tissue delivery challenges of the therapeutic strategy, and further discussed corresponding future optimization directions.

See the specific changes below:

Revised Abstract

"……These findings establish that cytoskeletal destabilization enables the adipogenic reprogramming of EOC cells via heterochromatin remodeling in the adipose-rich peritoneal niche, providing preclinical insights for novel ovarian cancer differentiation-targeted therapy."

Revised Discussion

"Although we have validated the anti-tumor efficacy of myosin II inhibition-mediated adipogenic transdifferentiation in subcutaneous and intraperitoneal ovarian cancer xenograft models, several limitations should be noted. Our study was performed in a limited set of ovarian cancer cell lines and conventional xenograft models, which cannot fully recapitulate the heterogeneity and complex microenvironment of primary human ovarian tumors, restricting the generalizability of our findings. …Of note, while the adipocyte-like cells generated from tumor cell transdifferentiation exhibited a stable, quiescent phenotype without evidence of pathological characteristics associated with cancer-associated adipocytes (CAAs), their long-term in vivo fate and potential to gradually acquire CAA-related tumor-promoting properties require further long-term observation and

characterization; additionally, the long-term stability of this transdifferentiation strategy has not been fully evaluated, and whether the adipocyte phenotype can be stably maintained or may undergo phenotypic reversal still requires further validation using lineage-tracing approaches."

If the authors increase statistical rigor and transparency, add pathway-level validation (GSEA), broaden and clinically anchor their model systems, consider single-cell resolution or at minimum strengthen conversion durability data, and expand in vivo endpoints and safety readouts, this work would make a meaningful contribution to the field.

Response:

We greatly appreciate your comprehensive evaluation and constructive suggestions.

We have substantially improved the statistical rigor and data transparency of the present study, supplemented relevant pathway validation analyses, optimized and clinically anchored the experimental model systems, strengthened the validation of adipogenic conversion durability, and expanded *in vivo* observational endpoints and safety evaluation readouts in the revised manuscript. These revisions have greatly refined our work, and we believe the current study can provide meaningful insights into the field of epithelial ovarian cancer therapeutic targeting *via* adipogenic reprogramming.

Response to Reviewers' Comments

Dear Editors and Reviewers,

Thank you for your continued evaluation and constructive feedback on our manuscript. We are grateful for the rigorous and insightful comments that have further guided us to improve the clarity, statistical rigor, and accuracy of our work. In response to the remaining concerns, we have carefully revised the manuscript with a focus on optimizing statistical analyses, standardizing quantitative reporting, and refining wording for more cautious and precise interpretation. All revisions and corrections in the tracked version are clearly highlighted in *red*.

Below are our point-by-point responses to the reviewers' comments.

Reviewer #1 (Remarks to the Author):

The authors have thoroughly addressed all comments raised in the previous review and provided clear and well-reasoned responses to the points of criticism. The revisions have significantly improved the manuscript. In its current form, the manuscript is suitable for publication.

Response：

We sincerely appreciate your positive feedback and full approval of our revised manuscript. Thank you very much for supporting its publication.

Reviewer #2 (Remarks to the Author):

The revised manuscript addresses a novel and potentially relevant concept: mechanical disruption of actomyosin contractility, together with adipogenic cues, may promote adipocyte-like reprogramming of ovarian cancer cells and reduce tumor progression. The authors have made a substantial effort to revise the work, including additional ovarian cancer cell lines, further in vivo validation, positive adipogenic controls, adipose-derived MSC experiments, and replacement of ACTB with GAPDH for qRT-PCR normalization. These additions improve the biological scope and are appreciated.

1. However, key statistical concerns remain insufficiently resolved. Many central conclusions rely on qRT-PCR panels with small replicate numbers, typically n = 3, and multiple genes tested in parallel. Several control qPCR groups still appear fixed at exactly 1 without visible SD/SEM. If these controls were artificially set as reference values rather than plotted as replicate-level normalized data, the biological variance of the control group is not displayed and the validity of the statistical tests becomes difficult to assess. The authors should replot qRT-PCR data to show replicate-level variability in both control and treated groups, provide raw Ct/ΔCt values, and clearly document how statistics were performed. The use of multiple unpaired t-tests across multi-gene qPCR and ChIP-qPCR panels also remains problematic unless appropriate multiple-testing correction is applied and reported.

Response：

We greatly appreciate the reviewer's rigorous and valuable comments on the statistical analyses of qRT-PCR and ChIP-qPCR data. We have comprehensively revised the data presentation, statistical tests and relevant documentation following your suggestions, as detailed below.

First, we have discontinued the practice of artificially setting the control group value to 1. All qRT-PCR results are now presented using raw ΔCt values (ΔCt = Ct(target gene) − Ct(*GAPDH*)). Both Ctrl and treated groups now exhibit genuine replicate-level variability with SEM error bars, fully reflecting biological and technical variation among samples. Meanwhile, all raw Ct and ΔCt values for qRT-PCR, as well as raw data for ChIP-qPCR, have been compiled into Raw Data tables to ensure full data transparency and traceability.

Second, we have fully updated the statistical strategies for both qRT-PCR and ChIP-qPCR assays. Considering the evident inter-batch variation across experiments, we replaced unpaired t-tests with two-sided paired t-tests for all analyses. For all multi-gene panels in both assays, we have applied rigorous multiple-testing correction using the two-stage step-up method (Benjamini, Krieger, and Yekutieli) with a false discovery rate (FDR) set at 5%, to eliminate potential false positives caused by multiple comparisons. The complete statistical approaches have been clearly documented in all corresponding figure legends and main text.

We fully acknowledge your concern regarding the use of three biological replicates (n = 3). Three independent biological replicates are a common experimental setting for cell-based qRT-PCR and

ChIP-qPCR studies in this field. To offset the potential limitations of a relatively small sample size, we adopted paired statistical design to correct batch effects, together with strict FDR control and multiple-testing correction. These combined measures effectively guarantee the robustness and reliability of our statistical results.

All revisions mentioned above have been marked in *red* in the revised manuscript.

Example:

Original Fig. 1 |… **c** qRT–PCR analysis of the mRNA levels of key adipogenic transcription factors, mature adipocyte markers, and fatty acid oxidation-related genes in Ble+BMP4-treated MEFs (*n* = 3). … Data are presented as mean ± SEM. *P < 0.05, ***P < 0.001, ****P < 0.0001. Statistical analyses were performed using two-sided unpaired Student's t-test in **(c, e, f)**.

Revised Fig. 1 |…**c** qRT-PCR analysis of ΔCt values (ΔCt = Ct (target gene) – Ct (*Gapdh*)) for key adipogenic transcription factors, mature adipocyte markers, and fatty acid oxidation-related genes in Ble+BMP4-treated MEFs (n = 3). Lower ΔCt values indicate higher mRNA expression levels. … Data are presented as mean ± SEM. **P < 0.01, ***P < 0.001, ****P < 0.0001. Statistical analyses for panels **(c, e, f)** were performed using two-sided paired Student's t-tests. For qRT PCR data **(c)**, paired two sided t tests were applied to ΔCt values, and multiple testing correction across genes was performed using the two stage step up method (Benjamini, Krieger, and Yekutieli; FDR = 5%).

2. In addition, the RNA-seq/GSEA results should be interpreted more cautiously, as some reported adipogenic enrichments are borderline or not statistically significant, while the manuscript still uses strong wording.

Response:

We appreciate your valuable suggestion to interpret RNA-seq and GSEA results more cautiously. We fully agree that some adipogenic enrichment results are not robust enough to support definitive conclusions. Accordingly, we have revised all relevant descriptions in both the main text and figure legends. We have removed overly strong and conclusive wording, and adopted more prudent expressions. The updated description is as follows: *Gene Set Enrichment Analysis (GSEA)* ***suggested a borderline enrichment*** *of the gene signature associated with positive regulation of fat cell differentiation (Fig. 2j).*

3. The response regarding MEF adipogenic differentiation is valuable. The added positive-control adipogenic induction kit and adipose-derived MSC data strengthen the experimental framework. However, these data mainly support that Blebbistatin can facilitate adipogenic conversion under adipogenic conditions; they do not fully establish that mechanical disruption alone is instructive.

Response:

We greatly appreciate the reviewer's insightful comment and precise distinction between a mechanical sensitizing/triggering effect and an instructive differentiation signal. We apologize for the misunderstanding caused by overly assertive phrasing in the original manuscript, and we fully clarify our experimental design and scientific logic as follows:

First, we did not include a "Ble alone" group in this study by deliberate scientific design, not by omission. Our previous published work has firmly demonstrated that Ble-mediated cytoskeletal disruption acts exclusively as a mechanical trigger/sensitizer—it cannot alone direct or induce any lineage-specific differentiation (including adipogenesis or neurogenesis). This well-established conclusion guided our experimental setup from the outset.

Second, Supplementary Fig. S1 was designed to validate that BMP4 alone is insufficient to trigger adipogenic conversion, which further supports our core model: robust lineage transdifferentiation occurs ONLY when mechanical perturbation (Ble) is combined with lineage-specific instructive cues (adipogenic signals in this study, neurogenic signals in our previous work). Ble itself provides no differentiation instructions; it only remodels chromatin and increases cellular responsiveness to external differentiation signals.

Third, we have comprehensively revised all ambiguous or overstated descriptions throughout the manuscript, including titles, section headings, main text, and figure legends. We have replaced terms implying independent instructive activity (e.g., "Ble alone promotes", "induces differentiation") with precise wording that defines Ble as a sensitizer/enabler that facilitates adipogenic transdifferentiation only in the presence of adipogenic cues.

Our experiments and conclusions strictly align: mechanical disruption serves as a permissive trigger to prime cells for differentiation, rather than an instructive signal that dictates cell fate. All revisions have been marked in red in the revised manuscript.

Example:

Original subtitle: Ble alone promotes adipogenic transdifferentiation in peritoneal micrometastases of ovarian cancer

Revised subtitle: Ble **facilitates** adipogenic transdifferentiation of ovarian cancer peritoneal micrometastases **in the endogenous adipose niche**

Overall, the revision improves the biological narrative, but the statistical and quantitative reporting still do not reach a fully satisfactory level. A focused revision addressing qPCR variance display, raw Ct/ΔCt transparency, multiple-testing correction, and more cautious interpretation of pathway analyses is required before the conclusions can be considered sufficiently supported.

Response:

We sincerely appreciate your constructive summary and valuable guidance. We have conducted a focused revision to fully address all remaining concerns regarding statistical and quantitative reporting, including the display of qPCR variance, transparency of raw Ct/ΔCt values, implementation of multiple-testing correction, and more cautious interpretation of pathway analyses. All revisions have been made to ensure the conclusions are rigorously supported by data, and relevant changes are marked in red in the revised manuscript.

Review of "Mechanical regulation of adipogenic reprogramming suppresses ovarian cancer progression" by Li et al.

Summary

Li et. al presented a study that investigated cytoskeletal regulation as a therapeutic strategy in epithelial ovarian cancer (EOC). The authors report that combining blebbistatin (Ble) with adipogenic cues (BMP4, PPARγ agonists) induces adipocyte-like transdifferentiation of ovarian cancer cells in vitro and in vivo, evidenced by lipid droplet accumulation, adipogenic marker expression, and β-adrenergic–responsive lipolysis. In mouse models, Ble treatment suppressed tumor growth, with HuNu/Perilipin-1 colocalization confirming tumor cell conversion. Mechanistically, cytoskeletal destabilization triggered heterochromatin remodeling, enabling CEBPα/CEBPβ activity. Adipogenic conversion also occurred on soft substrates and was blocked by constitutively nuclear YAP, highlighting mechanical signaling as a key regulator.

Overall impression:

The concept of mechanically induced reprogramming is relevant, and the authors apply a broad methodological toolkit of high quality. However, several claims, particularly regarding terminal differentiation and adipocyte morphology, are not convincingly supported by the data shown. The mere accumulation of neutral TAG in small lipid droplets stained by Bodipy and induction of LD-formating relevant genes does not indicate necessarily an adipocyte identity; true adipogenesis requires expression of adipokines such as ADIPOQ or LEPTIN, and the formation of large lipid droplets, which was not observed in Ble-treated cancer cells. Based on the current data, Ble-treated cells rather appear to undergo metabolic stress due to loss of actin–myosin structure, leading to ER perturbation and formation of small lipid droplets. The absence of large, fused lipid droplets typical of adipocytes suggests that lipid droplet maturation is impaired, and therefore the term "adipocytes" is not appropriate in this context.

Major points

1. **Terminology & Clarity**
    - Line 112: "terminally differentiated adipocytes" is incorrect; please use "adipocyte-like" True terminal adipocytes cannot be recapitulated in vitro within 14 days. See also "Overall impression" above.
2. **Lipid droplet accumulation vs. adipogenesis**
    - Data shown in Fig. S2 (ROSI treatment alone) showed stronger lipid accumulation in MEFs than Ble-treatment in terms of the number and size of Bodipy-stained cells, and the expression of PPARG/FABP4. Why do the authors stick to blebbistatin when ROSI has a stronger effect?
    - Fig. 2A shows numerous small lipid droplets in blebbistatin-treated A2780 cells that do not exhibit in vitro adipocyte morphology or lipid droplet fusion, as observed in Fig. S2A in MEFs. The authors should explain why they believe LD accumulation indicates adipogenesis rather than being merely a consequence of increased FA synthesis to maintain survival under cytoskeletal stress. Lipid droplets: a cellular organelle vital in cancer cells | Cell Death Discovery.
    - Also, PLIN1 staining appeared to be more cytoplasmic than LD-specific, with increased staining observed in almost all cells. At least, judging by the image presented. Does PLIN1 colocalise with LDs in Ble-treated A2780 cells?

- In this context RNA-seq data from "differentiated" A2780 cells as shown for MEFs in Fig. 1F shall be provided to support a "adipocyte"-like phenotype of Ble-treated A2780 cells.
- In Fig. 2c, the effect of Ble versus rosiglitazone (Rosi) treatment on peroxisome proliferator-activated receptor gamma (PPARG) and other differentiation markers was contrary to that observed in MEFs. What is the explanation for that?
- Although there is increased PLIN1 expression in vivo (see Figures 3 and 4), the cancer cell-derived cells do not exhibit typical adipocyte morphology, characterised by large, fusing lipid droplets. The author must explain this observation in the discussion section.

3. **Gene expression analysis**
    - In Fig. 2d the authors showed that "ACTB" is downregulated in RNAseq data. This makes this gene and unsuitable as a reference gene for quantitative real time PCR. Please re-analyze with validated stable reference genes (for example see Reliable reference genes for expression analysis of proliferating and adipogenically differentiating human adipose stromal cells - PubMed).
    - Clarify what is meant by "relative expression of mRNA", shown are fold changes vs. ctr of housekeeping genes normalized values?
    - Indicate whether SE or SD are shown in the figure legends?
4. **Cell line choice & Methodological clarification**
    - In figure 3, Ble was used always used in combination with BMP4 or ROSI but not administrated alone while in Figure 4 only Ble was used. Is there a reason for change in treatment strategy?
    - In Figure 3, the author used A2780 (ovarian cancer, therapy-sensitive) for the subcutaneous xenograft model, while SKOV3 (isolated from ascites, therapy-resistant) was used for intraperitoneal injection. Firstly, the main text and figure legends do not state that different cell types have been used. The authors must consistently refer to the cell types used in the figure captions and the main text to avoid confusing the reader. Secondly, there is no in vitro data showing that SKOV3 cells can "transdifferentiate" in a manner similar to A2780 cells. The authors must provide evidence showing whether or not SKOV3 behave similarly to A2780 cells in response to Ble-treatment.

Minor points.

8. **Fig. 1f:** Specify the selection criteria for the "top 50 differentially expressed genes."
9. **Fig. 3b / Fig. S4a:** provide statistical details (p-values; specify whether SE or SD is shown).
10. **Fig. 3j:** Lipid droplet morphology is not visible; please explain.
11. **Oil Red O staining:** clarify whether the peripheral staining in mouse tissue reflects tumor-associated lipid storage.
12. **Line 210 / Fig. 4d:** clarify whether Ble+BMP4-induced "transdifferentiation" is local, and explain what the perilipin+ areas in the vehicle controls represent.
13. **Fig. 3h vs. Fig. 4d:** clarify whether outcomes depend on tumor cell administration route or treatment condition.
14. **Line 273:** specify in the main text that ChIP-qPCR was used to demonstrate CEBPα/PPARγ binding to HP1/LMNA/C.
15. **Line 306 / Fig. 6:** specify which cell type was cultured on soft vs. stiff matrices (A2780 vs. MEFs). Clarify whether adipogenesis was induced solely by substrate stiffness or if additional cocktails were used.

16. **Substrate stiffness:** While it is true that 64 kPa (the Young's modulus of young cartilage) is soft relative to plastic (~GPa), it is much stiffer than adipose tissue (~0.5–2 kPa). What is the biological relevance of a 64 kPa 'soft' surface, and how does it relate to the adipogenic niche?
17. **Overexpression system:** Describe the system used to overexpress the YAP-5SA mutant. A control (e.g. YAP wt) is missing to confirm that the YAP-5SA mutant indeed negates the effects of the BLE on cells.
