# Supplementary material for: Mechanical regulation of adipogenic reprogramming suppresses ovarian cancer progression
Source: Commun Biol. 2026 Jun 29;9:1506. doi: 10.1038/s42003-026-10567-4 (PMC13752288; doi:10.1038/s42003-026-10567-4)
Supplement: Supplementary file 2 [file 42003_2026_10567_MOESM2_ESM.pdf]

Mechanical regulation of adipogenic reprogramming suppresses ovarian cancer progression

Supplementary Information

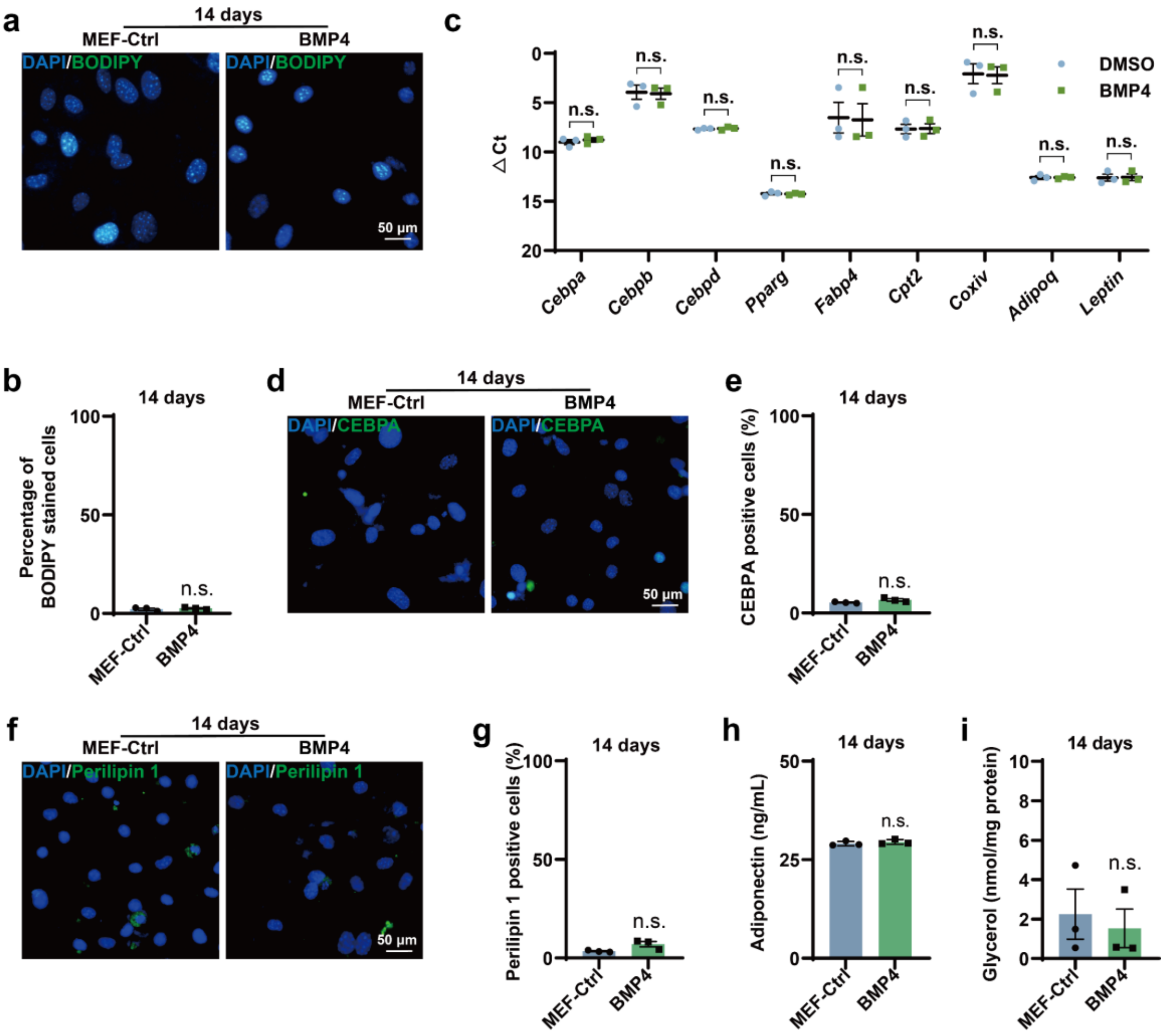

Supplementary Figure 1 | BMP4 alone does not induce the adipogenic transdifferentiation of MEFs. a Lipid droplets in BMP4-treated MEFs at D14 were visualized by BODIPY staining. Scale bars, 50 μm. **b** The percentage of BODIPY-stained cells were quantified (n = 3 independent experiments). **c** qRT–PCR analysis of ΔCt values (ΔCt = Ct (target gene) – Ct (*Gapdh*)) adipogenesis- and FAO-related genes in BMP4-treated MEFs at D14 (n = 3 independent experiments). Lower ΔCt values indicate higher mRNA expression levels. **d** Immunostaining for CEBPα (green) and nuclei (DAPI) in BMP4-treated MEFs at D14. Scale bar, 50 μm. **e** CEBPα-

positive cells were quantified (*n* = 3 independent experiments). **f** Immunostaining for Perilipin 1 (green) and nuclei (DAPI) in BMP4-treated MEFs at D14. Scale bars, 50 µm. **g** Perilipin 1-positive cells were quantified (*n* = 3 independent experiments). **h** Adiponectin secretion levels were measured for BMP4-treated MEFs by ELISA (*n* = 3 independent experiments). **i** Isoproterenol-induced lipolysis was assessed in BMP4-treated MEFs at D14 (*n* = 3 independent experiments). Random visual fields were selected from each group to avoid artificial selection bias for panel **b, e, g.** Data are presented as mean ± SEM. n.s., no significant difference ($P > 0.05$). Statistical analyses for panels **(b, e, g, h, i)** were performed using two-sided paired Student's *t*-tests. For qRT-PCR data **(c)**, paired two-sided *t*-tests were applied to ΔCt values, and multiple testing correction across genes was performed using the two-stage step-up method (Benjamini, Krieger, and Yekutieli; FDR = 5%).

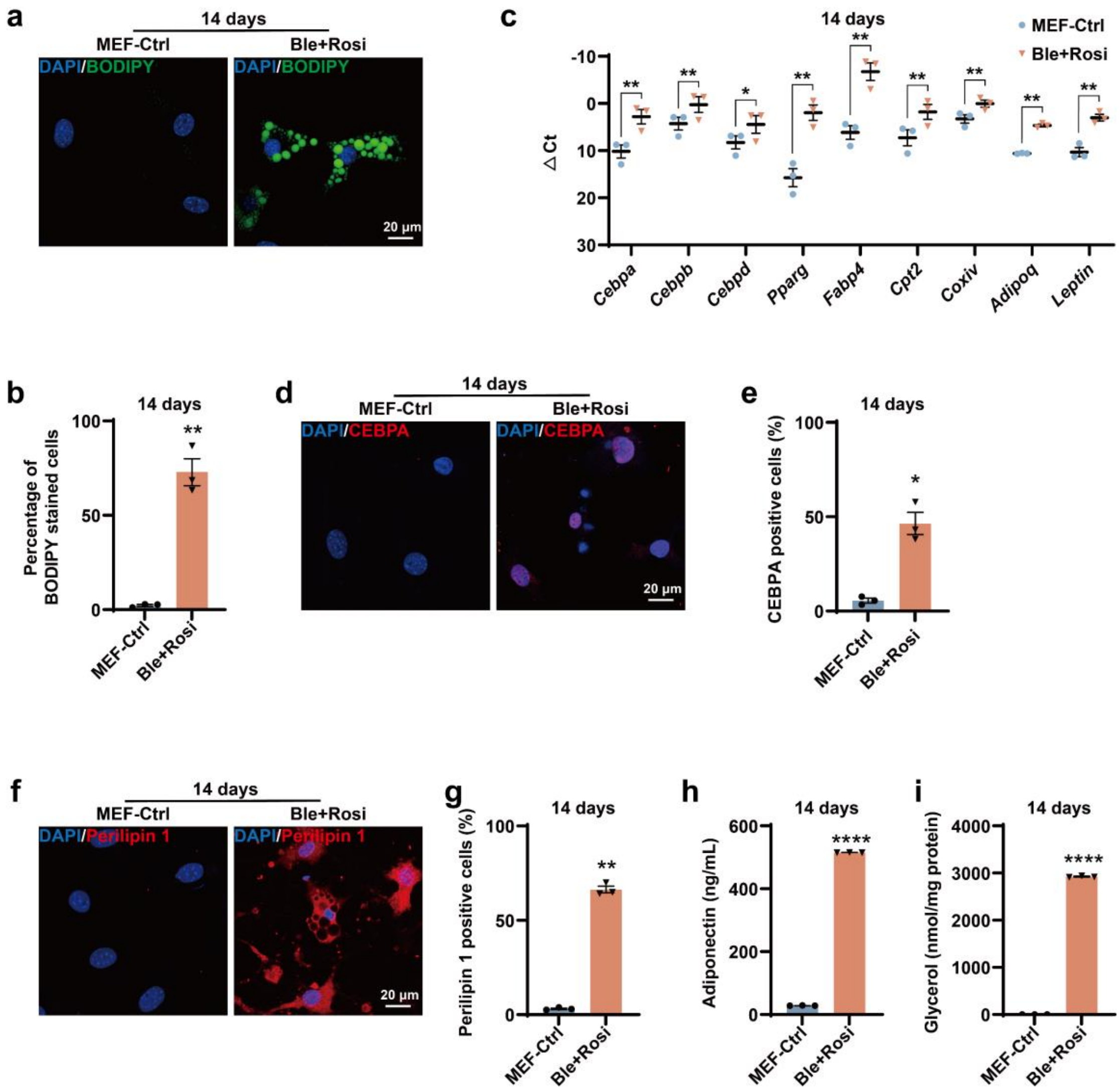

Supplementary Figure 2 | Ble+Rosi induces adipocyte transdifferentiation in MEFs. a Lipid droplets in Ble+Rosi treated MEFs were visualized by BODIPY staining. Scale bars, 50 µm. **b** The percentage of BODIPY-stained cells were quantified (*n* = 3 independent experiments). **c** qRT–PCR analysis of ΔCt values (ΔCt = Ct (target gene) – Ct (*Gapdh*)) for adipogenesis- and FAO-related genes in Ble+Rosi treated MEFs (*n* = 3 independent experiments). Lower ΔCt values indicate higher mRNA expression levels. **d** Immunostaining for CEBPα (red) and nuclei (DAPI) in Ble+Rosi treated MEFs. Scale bar, 50 µm. **e** CEBPα-positive cells were quantified (*n* = 3 independent experiments). **f** Immunostaining for Perilipin 1 and nuclei (DAPI) in Ble+Rosi treated MEFs. scale bars, 50 µm. **g** Perilipin 1-positive cells were quantified (*n* = 3 independent experiments). **h** Adiponectin secretion levels were measured for Ble+Rosi treated MEFs by ELISA (*n* = 3 independent experiments). **i** Isoproterenol-induced lipolysis was measured in Ble+Rosi treated

MEFs (n = 3 independent experiments). Random visual fields were selected from each group to avoid artificial selection bias for panel **b, e, g.** Data are presented as mean ± SEM. *$P < 0.05$, **$P < 0.01$, ***$P < 0.001$, ****$P < 0.0001$. Statistical analyses for panels **(b, e, g, h, i)** were performed using two-sided paired Student's t-tests. For qRT-PCR data **(c)**, paired two-sided t-tests were applied to ΔCt values, and multiple testing correction across genes was performed using the two-stage step-up method (Benjamini, Krieger, and Yekutieli; FDR = 5%).

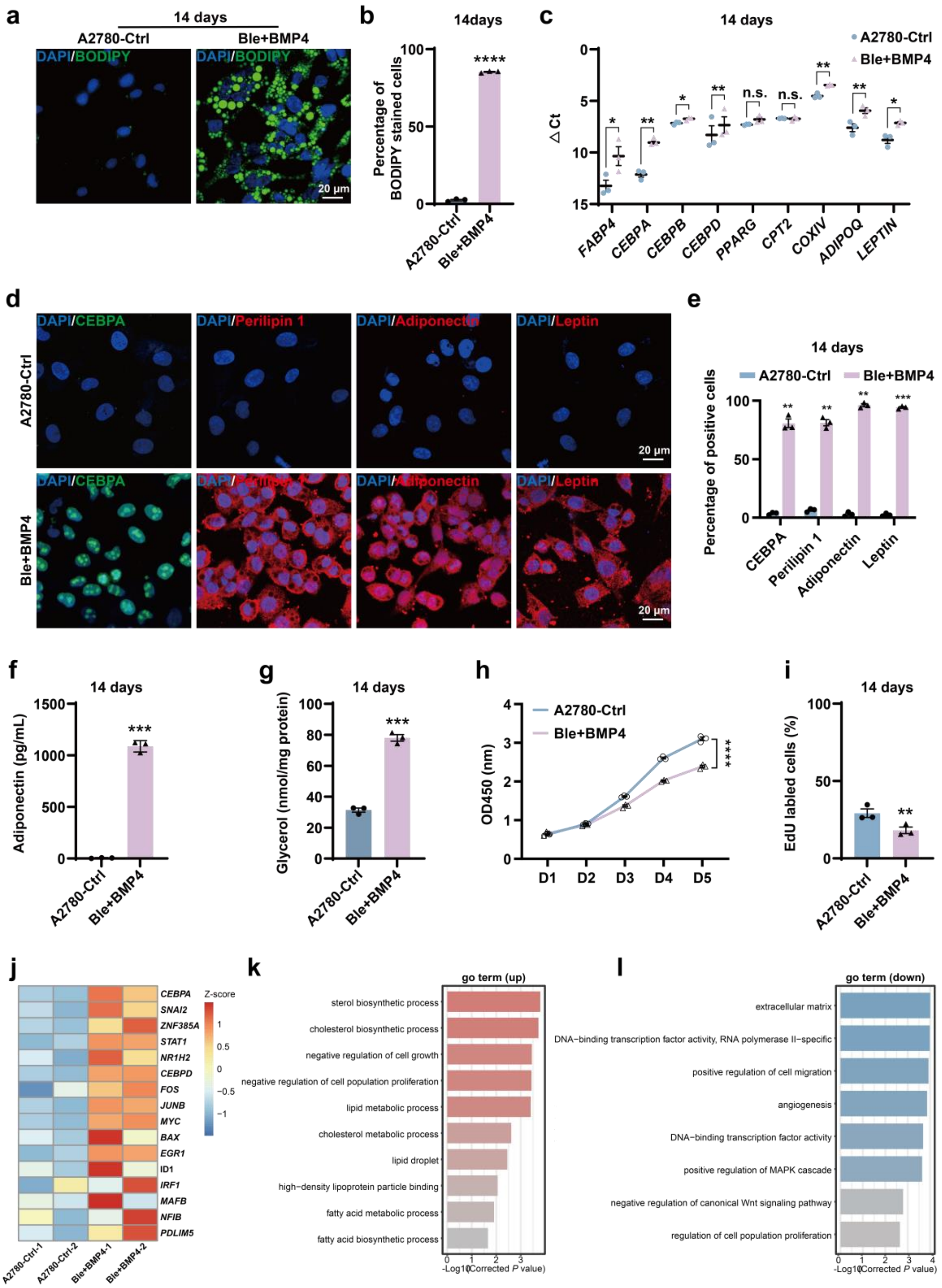

Supplementary Figure 3 | Ble plus adipogenic cues induce A2780 adipogenic transdifferentiation and inhibit proliferation *in vitro*. a Lipid droplets in Ble+BMP4 treated A2780 cells at D14 were visualized by BODIPY staining. Scale bars, 20 μm. **b** The percentage of

BODIPY-stained cells was quantified ($n = 3$ independent experiments). **c** qRT–PCR analysis of ΔCt values (ΔCt = Ct (target gene) − Ct (*GAPDH*)) for adipocyte-specific genes in Ble+BMP4 treated A2780 cells at D14 ($n = 3$ independent experiments). Lower ΔCt values indicate higher mRNA expression levels. **d** Immunostaining for CEBPα (green), Perilipin 1(red), Adiponectin (red), Leptin (red) and nuclei (DAPI) in Ble+BMP4 treated A2780 cells at D14; scale bar, 20 μm. **e** CEBPα-, Perilipin 1-, Adiponectin- and Leptin- positive cells were quantified ($n = 3$ independent experiments). **f** Adiponectin secretion levels in Ble+BMP4 treated A2780 cells at D14 were measured by ELISA ($n = 3$ independent experiments). **g** Isoproterenol-induced lipolysis was measured in Ble+BMP4 treated A2780 cells at D14 (n = 3 independent experiments). **h** CCK-8 analysis of Ble+BMP4 treated A2780 cells revealed a lower cell proliferation rate (n = 3 independent experiments). **i** Ble+BMP4 treated A2780 cells were incubated with EdU for 2.5 hr to label proliferating cells. EdU-labeled cells were quantified using ImageJ ($n = 3$ independent experiments). **j** Heatmap showing differential expression of adipogenesis-related transcription factors in A2780 cells with or without Ble+BMP4 treatment. **k, l** Bar plot of selected functional enrichment terms of genes upregulated and downregulated in Ble+BMP4 treated A2780 cells. Random visual fields were selected from each group to avoid artificial selection bias for panel **b, e, i.** Data are presented as mean ± SEM. *$P < 0.05$, **$P < 0.01$, ***$P < 0.001$, ****$P < 0.0001$. n.s., no significant difference ($P > 0.05$). Statistical analyses for panels **(b, f, g, i)** were performed using two-sided paired Student's *t*-tests. For panels **(c, e)**, paired two-sided *t*-tests were applied, and multiple testing correction across genes or proteins was performed using the two-stage step-up method (Benjamini, Krieger, and Yekutieli; FDR = 5%). For panel **(h)**, proliferation rates were

derived from linear regression of the growth curves, and significance between groups was calculated using an unpaired two-sided Student's t-test on the regression slopes.

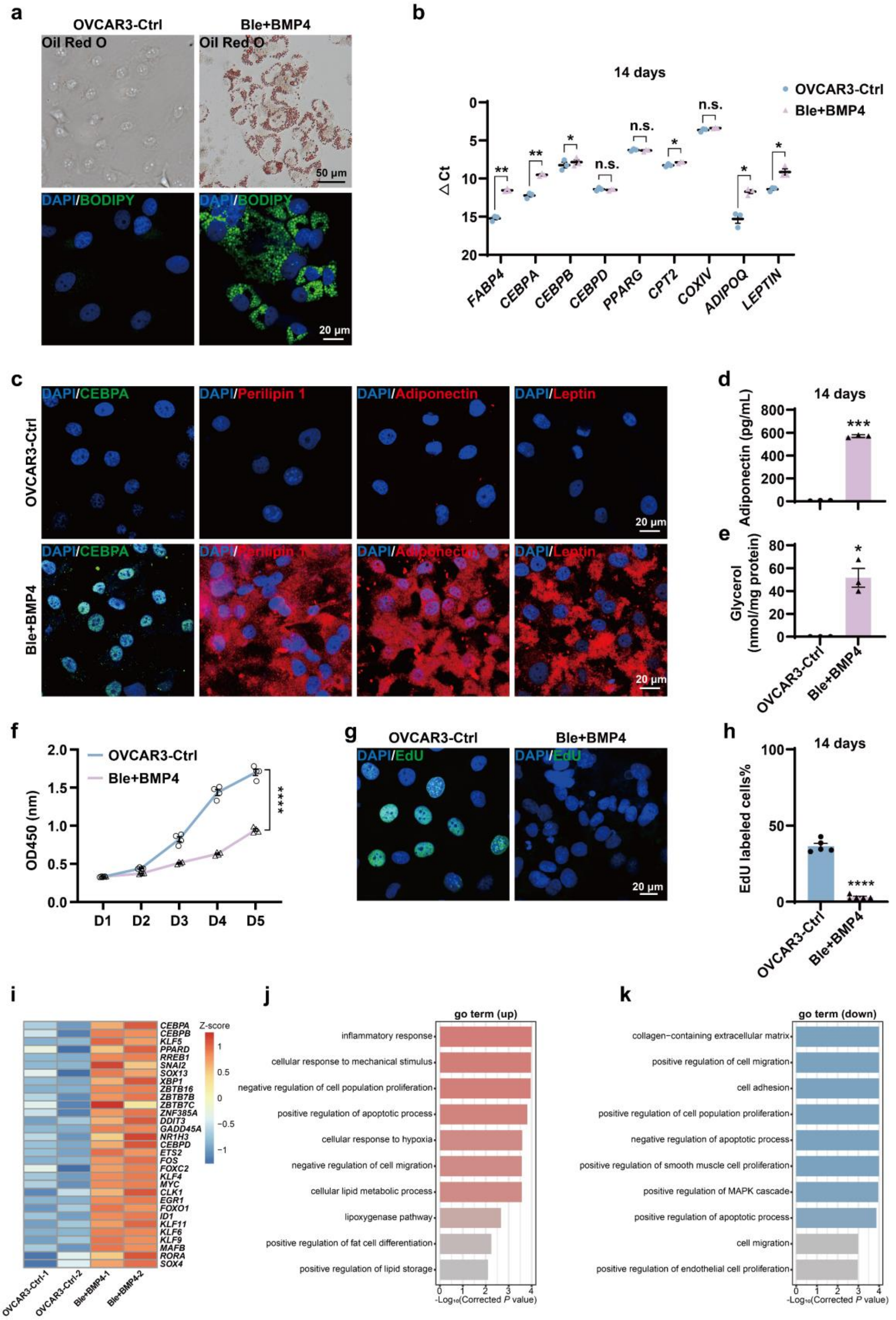

Supplementary Figure 4 | Ble plus adipogenic cues induce OVCAR3 adipogenic transdifferentiation and inhibit proliferation. **a** Lipid droplets in Ble+BMP4 treated OVCAR3 cells at D14 were visualized by Oil Red O and BODIPY staining. Scale bars, 50 μm and 20 μm. **b** qRT–PCR analysis of ΔCt values (ΔCt = Ct (target gene) – Ct (*GAPDH*)) for adipocyte-specific genes in Ble+BMP4 treated OVCAR3 cells at D14 (n = 3 independent experiments). Lower ΔCt values indicate higher mRNA expression levels. **c** Immunostaining for CEBPα (green), Perilipin 1(red), Adiponectin (red), Leptin (red) and nuclei (DAPI) in Ble+BMP4 treated OVCAR3 cells at D14; scale bar, 20 μm. **d** Adiponectin secretion levels in Ble+BMP4 treated OVCAR3 cells at D14 were measured by ELISA (n = 3 independent experiments). **e** Isoproterenol-induced lipolysis was measured in Ble+BMP4 treated OVCAR3 cells at D14 (n = 3 independent experiments). **f** CCK-8 analysis of Ble+BMP4 treated OVCAR3 cells revealed a lower cell proliferation rate (n = 3 independent experiments). **g, h** Ble+BMP4 treated OVCAR3 cells were incubated with EdU for 2.5 hr to label proliferating cells. EdU-labeled cells were quantified using ImageJ (n = 3 independent experiments), scale bar, 20 μm. **i** Heatmap showing differential expression of adipogenesis-related transcription factors in OVCAR3 cells with or without Ble+BMP4 treatment. **j, k** Bar plot of selected functional enrichment terms of genes upregulated and downregulated in Ble+BMP4 treated OVCAR3 cells. Random visual fields were selected from each group to avoid artificial selection bias for panel **h.** Data are presented as mean ± SEM. *P < 0.05, **P < 0.01, ***P < 0.001, ****P < 0.0001. n.s., no significant difference (P > 0.05). Statistical analyses for panels **(d, e, h)** were performed using two-sided paired Student's t-tests. For qRT-PCR data **(b)**, paired two-sided t-tests were applied to ΔCt values, and multiple testing correction across genes was performed using the two-stage step-up method (Benjamini, Krieger, and Yekutieli; FDR = 5%). For panel **(f)**, proliferation rates were derived from linear regression of the growth curves, and significance between groups was calculated using an unpaired two-sided Student's t-test on the regression slopes.

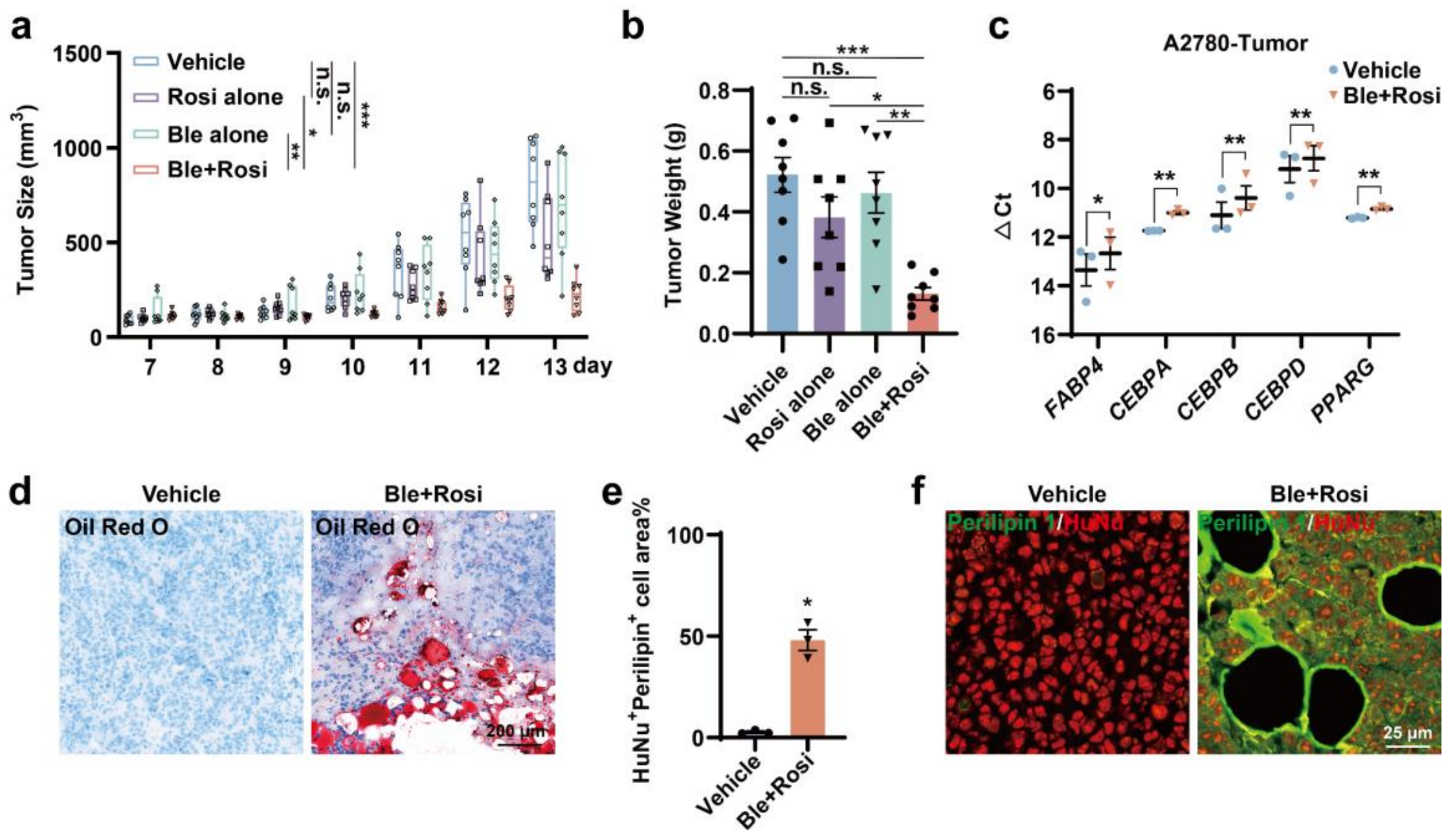

Supplementary Figure 5 | Ble+Rosi treatment inhibits tumor growth and induces adipogenic transdifferentiation of A2780 cells *in vivo*. a Tumor volume growth curves are shown during the treatment time course (n = 8 animals for each group, P = 0.1057 for Vehicle *vs* Rosi, P = 0.5654 for Vehicle *vs* Ble, P = 0.0001 for Vehicle *vs* Ble+Rosi, P = 0.0445 for Rosi *vs* Ble+Rosi, P = 0.0033 for Ble *vs* Ble+Rosi,). Vehicle and Ble alone groups are shared with Fig. 3b. **b** Tumor weights are shown after 14 days of treatment (n = 8 animals). Vehicle and Ble alone groups are shared with Fig. 3c. **c** qRT–PCR analysis of ΔCt values (ΔCt = Ct (target gene) – Ct (*GAPDH*)) for adipocyte-specific genes in A2780 subcutaneous tumors from Ble+Rosi treated mice (n = 3 biologically independent samples). Lower ΔCt values indicate higher mRNA expression levels. **d** Representative images of tumor sections stained with Oil Red O. Scale bars, 200 μm. **e** The area occupied by HuNu and Perilipin 1 copositive cells was quantified (n = 3 biologically independent samples). **f** Local zoom images of tumor sections that were stained with HuNu (red) and Perilipin 1 (green). Scale bars, 25 μm. Random visual fields were selected from each group to avoid artificial selection bias for panel **e.** Data are presented as mean ± SEM. *$P < 0.05$, **$P < 0.01$, ***$P < 0.001$, ****$P < 0.0001$. n.s.,

no significant difference ($P > 0.05$). For panel **(a)** (tumor growth rates derived from linear regression of individual tumor volume curves) and panel **(b)** (tumor weights), data were analyzed by one-way ANOVA with Tukey's test (panel **(a)**, equal variances) or Welch's ANOVA with Dunnett's T3 (panel **(b)**, unequal variances). All four groups (Vehicle, Rosi alone, Ble alone, Ble+Rosi) were compared pairwise. For qRT-PCR data **(c)**, paired two-sided *t*-tests were applied to ΔCt values, and multiple testing correction across genes was performed using the two-stage step-up method (Benjamini, Krieger, and Yekutieli; FDR = 5%). For panel **(e)**, a two-sided paired Student's *t*-test was used.

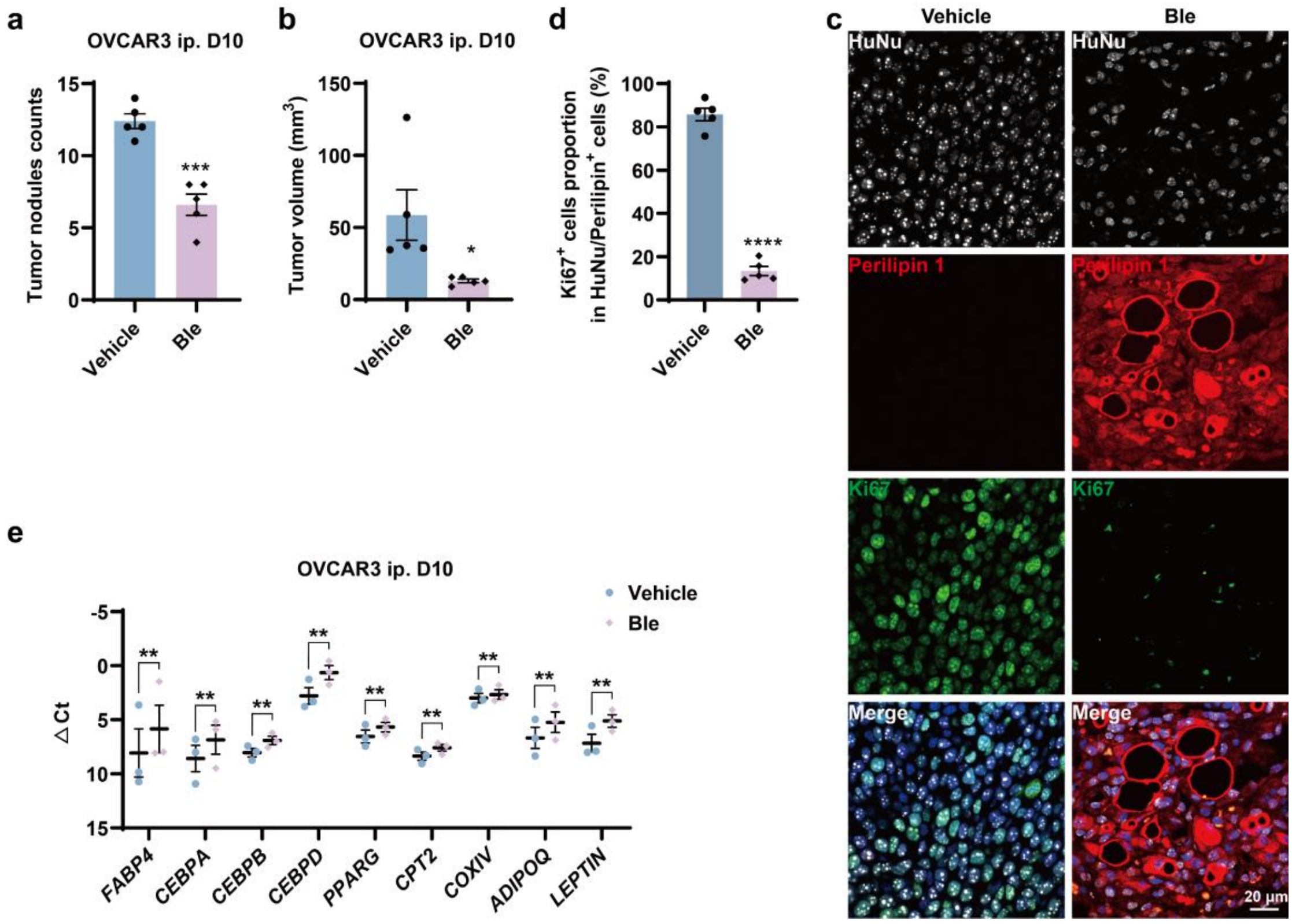

Supplementary Figure 6 | Ble inhibits OVCAR3 tumor growth and facilitates adipogenic transdifferentiation in the peritoneal adipose niche. a Tumor nodules counts are shown after 10 days of treatment (n = 5 animals). **b** Tumor weights are shown after 10 days of treatment (n = 5

animals). **c** Representative images of tumor sections that were stained with HuNu (white), Ki67 (green), and Perilipin 1 (red). Scale bars, 20 μm. **d** The Ki67-positive cells fraction in HuNu and Perilipin 1 co-positive cells was quantified ($n = 5$ biologically independent samples). **e** qRT–PCR analysis of ΔCt values (ΔCt = Ct (target gene) − Ct (*GAPDH*)) for adipocyte-specific genes in abdominal tumors from Ble treated mice ($n = 3$ biologically independent samples). Lower ΔCt values indicate higher mRNA expression levels. Random visual fields were selected from each group to avoid artificial selection bias for panel (**d**)**.** Data are presented as mean ± SEM. *$P < 0.05$, **$P < 0.01$, ***$P < 0.001$, ****$P < 0.001$. For panel **(a, b)**, a two-sided unpaired Student's *t*-test was used. For panels **(d)**, two-sided paired Student's *t*-tests were applied. For qRT-PCR data **(e)**, paired two-sided *t*-tests were applied to ΔCt values, and multiple testing correction across genes was performed using the two-stage step-up method (Benjamini, Krieger, and Yekutieli; FDR = 5%).

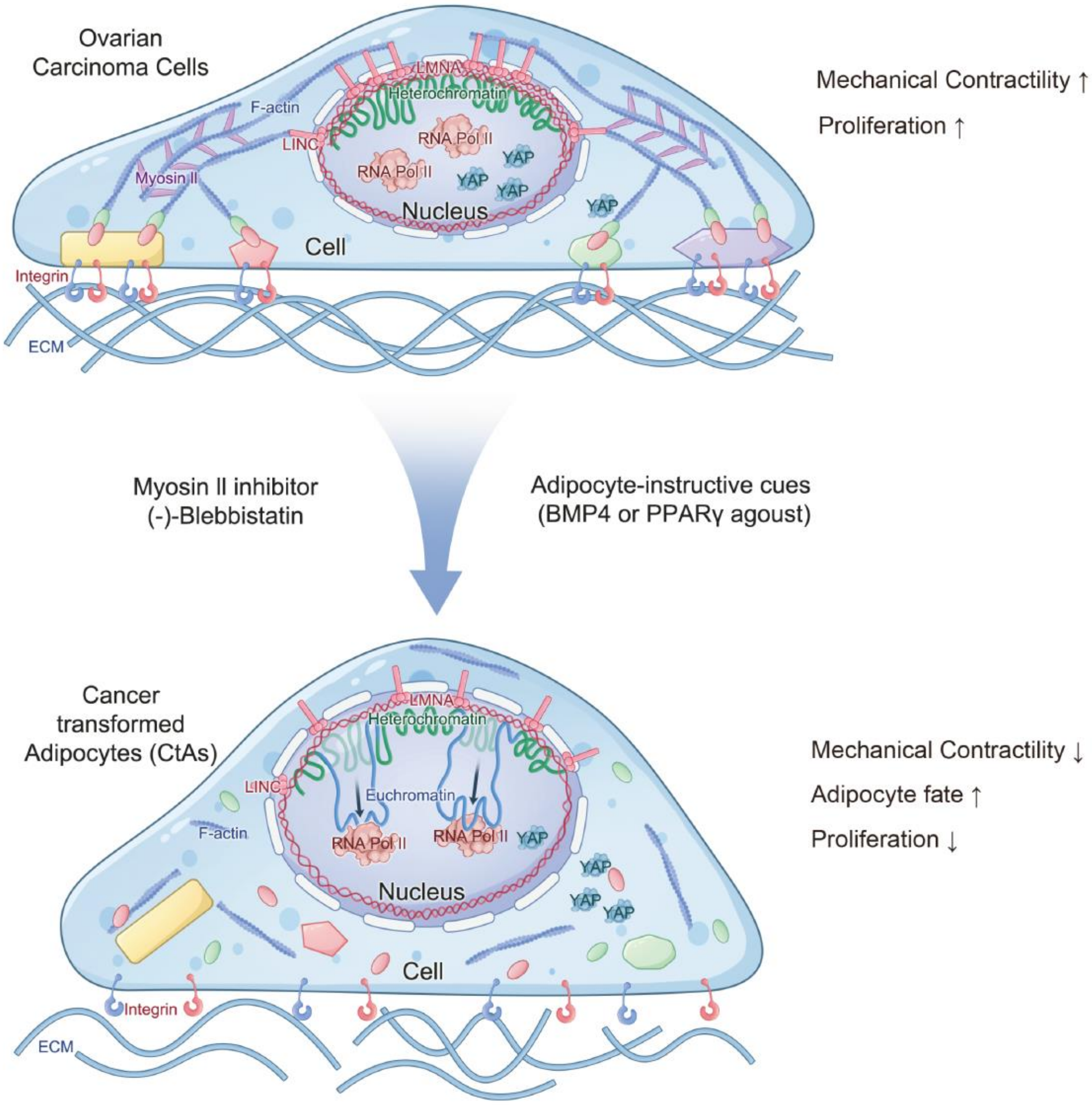

Supplementary Figure 7 | The mechanism of adipogenic transdifferentiation of ovarian carcinoma cells. Mechanical destabilization of the cytoskeleton by Ble triggers mechano-epigenetic remodeling, which involves the redistribution of constitutive heterochromatin and increased chromatin accessibility. This establishes an epigenetically permissive state that facilitates the binding of pioneer transcription factors. These factors synergize with adipogenic stimuli to drive the adipogenic transdifferentiation of ovarian cancer cells while concomitantly restraining their proliferation. Moreover, this process depends on YAP nuclear export.

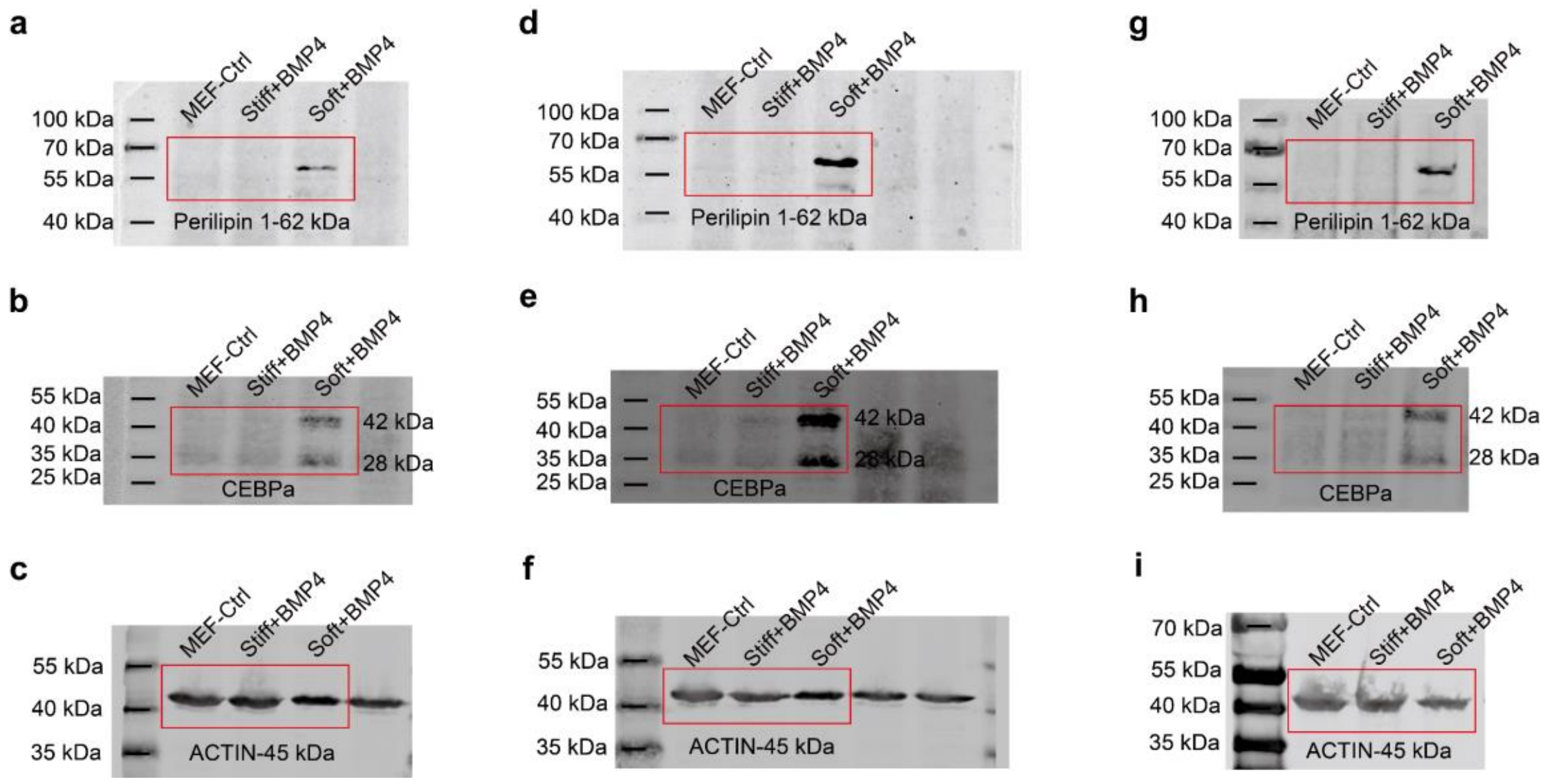

Supplementary Figure 8 | Full-length Western blot images corresponding to Fig. 6e. (a–c) First replicate: **(a)** Perilipin 1, **(b)** CEBPA, **(c)** ACTIN. The cropped blots presented in **Fig. 6e** are derived from the regions indicated by red boxes in **(a–c)**. **(d–f)** Second replicate: **(d)** Perilipin 1, **(e)** CEBPA, **(f)** ACTIN. **(g–i)** Third replicate: **(g)** Perilipin 1, **(h)** CEBPA, **(i)** ACTIN. Molecular weight markers are indicated on the left. Uncropped scans are provided as source data.

Supplementary Table 1: Primers for qRT-PCR (Mouse)

Primers for qRT-PCR (Mouse)	
Fabp4- F	AAGGTGAAGAGCATCATAACCCT
Fabp4- R	TCACGCCTTTCATAACACATTCC
Cebpa- F	CAAGAACAGCAACGAGTACCG
Cebpa- R	GTCACTGGTCAACTCCAGCAC
Cebpb- F	GGGGTTGTTGATGTTTTTGG
Cebpb- R	CGAAACGGAAAAGGTTCTCA
Cebpd- F	CGACTTCAGCGCCTACATTGA
Cebpd- R	GAAGAGGTCGGCGAAGAGTT
Pparg-F	TGTGGACCTCTCCGTGATGG
Pparg-R	GGTTCTACTTTGATCGCACTTTGG
Cpt2-F	CCTGCTCGCTCAGGATAAACA
Cpt2-R	GTGTCTTCAGAAACCGCACTG
Coxiv-F	ATTGGCAAGAGAGCCATTTCTAC
Coxiv-R	CACGCCGATCAGCGTAAGT
Mcad-F	CCGAAGAGTTGGCGTATGGG
Mcad-R	GGGCTCTGTCACACAGTAAGC
Lcad-F	TTTCCTCGGAGCATGACATTTT
Lcad-R	GCCAGCTTTTTCCCAGACCT
Adipoq-F	TGTTCCTCTTAATCCTGCCCA
Adipoq-R	CCAACCTGCACAAGTTCCCTT
Leptin-F	GAGACCCCTGTGTCGGTTC
Leptin-R	CTGCGTGTGTGAAATGTCATTG
Gapdh-F	AGGTCGGTGTGAACGGATTTG
Gapdh-R	TGTAGACCATGTAGTTGAGGTCA

Supplementary Table 2: Primers for qRT-PCR (Human)

Primers for qRT-PCR (Human)	
FABP4-F	ACTGGGCCAGGAATTTGACG
FABP4-R	CTCGTGGAAGTGACGCCTT
CEBPA-F	TTGTGCCTTGGAAATGCAAC
CEBPA-R	TCGGGAAGGAGGCAGGAAAC
CEBPB-F	CAAGCACAGCGACGAGTACAAGATCC
CEBPB-R	GCTTGAACAAGTTCCGCAGGGTG
CEBPD-F	GCCATGTACGACGACGAGA
CEBPD-R	TTGCTGTTGAAGAGGTCGG
PPARG-F	GGGATCAGCTCCGTGGATCT
PPARG-R	TGCACTTTGGTACTCTTGAAGTT
COXIV-F	CAGGGTATTTAGCCTAGTTGGC
COXIV-R	GCCGATCCATATAAGCTGGGA
CPT2-F	CATACAAGCTACATTTCGGGACC
CPT2-R	AGCCCGGAGTGTCTTCAGAA
ADIPOQ-F	GGCTTTCCGGGAATCCAAGG
ADIPOQ-R	TGGGGATAGTAACGTAAGTCTCC
LEPTIN-F	TGCCTTCCAGAAACGTGATCC
LEPTIN-R	CTCTGTGGAGTAGCCTGAAGC
GAPDH-F	GGAGCGAGATCCCTCCAAAAT
GAPDH-R	GGCTGTTGTCATACTTCTCATGG

Supplementary Table 3: Primers for ChIP-qPCR

Primers for ChIP-qPCR	
Cebpa-F	CAGGAGTCAGTGGGCGTTG
Cebpa-R	TGACTTTCCAAGGCGGTGAG
Cebpb-F	CAATGACGCGCACCGAC
Cebpb-R	TGGCCACTTCCATGGGTCTA
Cebpd-F	TGTTCCGCCTTTGCTATGTCT
Cebpd-R	TTGTGCGGTTTGGACCTACT
Pparg-F	GACATCGGTCTGAGGGACAC
Pparg-R	TTACCTGGCCGCCTTGC

Supplementary Table 4: Primary antibodies

Primary antibodies	Brand	Cat#
C/EBPα (D56F10) XP® Rabbit mAb #8178	CST	8178S
Perilipin 1 (D1D8) XP® Rabbit mAb #9349	CST	9349S
Adiponectin Polyclonal antibody	Proteintech	21613-1-AP
Leptin Polyclonal antibody	Proteintech	17436-1-AP
Anti-Ki67antibody	Abcam	ab15580
Anti-Human Nucleoli antibody [NM95] - Nucleolar Marker	Abcam	ab190710
HP1α Antibody	CST	2616S
Anti-Histone H3 (tri methyl K9) antibody	Abcam	ab8898
Anti-Histone H3 (acetyl K27) antibody [EP16602]	Abcam	ab177178
Anti-RNA polymerase II CTD repeat YSPTSPS (phospho S5) antibody [4H8]	Abcam	ab5408
Anti-β-Actin antibody	Sigma	A1978
