# Supplementary material for: Mechanical regulation of adipogenic reprogramming suppresses ovarian cancer progression
Source: Commun Biol. 2026 Jun 29;9:1506. doi: 10.1038/s42003-026-10567-4 (PMC13752288; doi:10.1038/s42003-026-10567-4)
Supplement: Supplementary file 3 [file 42003_2026_10567_MOESM3_ESM.pdf]

Description of Additional Supplementary Files

File name: Supplementary Data 1

Description: The source data behind the graphs in the paper.
