# Supplementary material for: Mechanical regulation of adipogenic reprogramming suppresses ovarian cancer progression
Source: Commun Biol. 2026 Jun 29;9:1506. doi: 10.1038/s42003-026-10567-4 (PMC13752288; doi:10.1038/s42003-026-10567-4)
Supplement: Supplementary file 4 [file 42003_2026_10567_MOESM5_ESM.pdf]

Corresponding author(s): DAPR COMMSBIO-25-8167C

Last updated by author(s): Jun 16, 2026

Reporting Summary

Nature Portfolio wishes to improve the reproducibility of the work that we publish. This form provides structure for consistency and transparency in reporting. For further information on Nature Portfolio policies, see our Editorial Policies and the Editorial Policy Checklist.

Statistics

For all statistical analyses, confirm that the following items are present in the figure legend, table legend, main text, or Methods section.

n/a	Confirmed	
☐	☒	The exact sample size (*n*) for each experimental group/condition, given as a discrete number and unit of measurement
☐	☒	A statement on whether measurements were taken from distinct samples or whether the same sample was measured repeatedly
☐	☒	The statistical test(s) used AND whether they are one- or two-sided<br>*Only common tests should be described solely by name; describe more complex techniques in the Methods section.*
☒	☐	A description of all covariates tested
☐	☒	A description of any assumptions or corrections, such as tests of normality and adjustment for multiple comparisons
☐	☒	A full description of the statistical parameters including central tendency (e.g. means) or other basic estimates (e.g. regression coefficient) AND variation (e.g. standard deviation) or associated estimates of uncertainty (e.g. confidence intervals)
☒	☐	For null hypothesis testing, the test statistic (e.g. *F*, *t*, *r*) with confidence intervals, effect sizes, degrees of freedom and *P* value noted<br>*Give P values as exact values whenever suitable.*
☒	☐	For Bayesian analysis, information on the choice of priors and Markov chain Monte Carlo settings
☒	☐	For hierarchical and complex designs, identification of the appropriate level for tests and full reporting of outcomes
☒	☐	Estimates of effect sizes (e.g. Cohen's *d*, Pearson's *r*), indicating how they were calculated

Our web collection on statistics for biologists contains articles on many of the points above.

Software and code

Policy information about availability of computer code

Data collection	Microscope and imaging system built in software (Olympus cellSens, IVIS Lumina XR imaging system, Nikon NIS Elements) were used for image acquisition.
Data analysis	Data were analyzed using the following softwares, as detailed in the methods: ImageJ 1.54g, Living Image software (v4.7.2), Nis Elements Imaging Software (version 5.21.00), and GraphPad Prism 9.5.0.<br>All RNA-sequencing data analyses were performed with STAR (version 2.7.11a) and StringTie (version v2.1.7) using the UCSC hg38 annotation with default settings. Gene Ontology (GO) analysis was performed using KOBAS. Principal component analysis (PCA), heatmaps, and functional enrichment bar plots were generated using prcomp, pheatmap, and ggplot2 functions in R. The interaction network of the differentially expressed TFs was constructed using the STRING online tool STRING: functional protein association networks (https://string-db.org/) using Cytoscape. The Gene Set Enrichment Analysis was performed using the ''GSEA'' function and visualized through the ''gseaplot2'' function in the R package clusterProfiler.

For manuscripts utilizing custom algorithms or software that are central to the research but not yet described in published literature, software must be made available to editors and reviewers. We strongly encourage code deposition in a community repository (e.g. GitHub). See the Nature Portfolio guidelines for submitting code & software for further information.

Data

Policy information about availability of data

All manuscripts must include a data availability statement. This statement should provide the following information, where applicable:

- Accession codes, unique identifiers, or web links for publicly available datasets
- A description of any restrictions on data availability
- For clinical datasets or third party data, please ensure that the statement adheres to our policy

RNA-seq data of MEFs have been deposited in the Genome Sequence Archive of the Beijing Institute of Genomics, Chinese Academy of Sciences (https://ngdc.cncb.ac.cn/). The accession number is CRA024556. RNA-seq data of A2780, SKOV3, and OVCAR3 cells (accession number: HRA018018) and A2780 subcutaneous tumors (accession number is HRA011043) have been deposited in the Genome Sequence Archive for human (GSA-Human) of the Beijing Institute of Genomics, Chinese Academy of Sciences (https://ngdc.cncb.ac.cn/gsa-human/).

Research involving human participants, their data, or biological material

Policy information about studies with human participants or human data. See also policy information about sex, gender (identity/presentation), and sexual orientation and race, ethnicity and racism.

Reporting on sex and gender	All human cell lines used are derived from female donors; no new participant data were collected, and no sex/gender-specific analyses were performed.
Reporting on race, ethnicity, or other socially relevant groupings	Donor ethnicity is known (A2780: African American; SKOV3/OVCAR3: White/Caucasian), and no race/ethnicity-specific analyses were performed.
Population characteristics	No new human participants were enrolled, and no population-level analyses were conducted in this study.
Recruitment	No human participants were recruited; all human cell lines used are previously established and publicly available.
Ethics oversight	This study uses only publicly available, established human cell lines, so no additional ethical approval is required.

Note that full information on the approval of the study protocol must also be provided in the manuscript.

Field-specific reporting

Please select the one below that is the best fit for your research. If you are not sure, read the appropriate sections before making your selection.

- [x] Life sciences
- [ ] Behavioural & social sciences
- [ ] Ecological, evolutionary & environmental sciences

For a reference copy of the document with all sections, see nature.com/documents/nr-reporting-summary-flat.pdf

Life sciences study design

All studies must disclose on these points even when the disclosure is negative.

Sample size	For in vivo mouse studies, we have projected group size estimates based on previous experiences and published studies with similar layouts.
Data exclusions	no data was excluded.
Replication	The number of replicates for each experiment is indicated in figure legends. Within each experimental group, the reproducibility was successful, although a degree of variability was detected due to inter- individual diversity.
Randomization	No randomization was applied.
Blinding	No blinding was used.

Reporting for specific materials, systems and methods

We require information from authors about some types of materials, experimental systems and methods used in many studies. Here, indicate whether each material, system or method listed is relevant to your study. If you are not sure if a list item applies to your research, read the appropriate section before selecting a response.

Materials & experimental systems

n/a	Involved in the study	
☐	☒	Antibodies
☐	☒	Eukaryotic cell lines
☒	☐	Palaeontology and archaeology
☐	☒	Animals and other organisms
☒	☐	Clinical data
☒	☐	Dual use research of concern
☒	☐	Plants

Methods

n/a	Involved in the study	
☒	☐	ChIP-seq
☒	☐	Flow cytometry
☒	☐	MRI-based neuroimaging

Antibodies

Antibodies used	C/EBPα (D56F10) XP® Rabbit mAb #8178, Cell Signaling Technology, 8178S;<br>Perilipin 1 (D1D8) XP® Rabbit mAb #9349, Cell Signaling Technology, 9349S;<br>Adiponectin Polyclonal antibody, Proteintech, 21613-1-AP;<br>Leptin Polyclonal antibody, Proteintech, 17436-1-AP;<br>Anti-Ki67antibody, Abcam, ab15580;<br>Anti-Human Nucleoli antibody [NM95] - Nucleolar Marker, Abcam, ab190710;<br>HP1α Antibody, Cell Signaling Technology, 2616S;<br>Anti-Histone H3 (tri methyl K9) antibody, Abcam, ab8898;<br>Anti-Histone H3 (acetyl K27) antibody [EP16602], Abcam, ab177178;<br>Anti-RNA polymerase II CTD repeat YSPTSPS (phospho S5) antibody [4H8], Abcam, ab5408;<br>Anti-β-Actin antibody, Sigma, A1978.
Validation	All antibodies used were purchased from commercial sources and reputable vendors (eg. Abcam, Cell Signaling Technology). Antibody specificity and quality validation were performed by the manufacturers, including for the species and application. Validation statements of the manufacturers can be found on their webpages (direct testing or by providing adequate references). Antibodies were aliquoted and stored as recommended by the manufacturer to minimize freeze thaw cycles and associated loss in performance.

Eukaryotic cell lines

Policy information about cell lines and Sex and Gender in Research

Cell line source(s)	Mouse embryonic fibroblasts (MEFs) were established from CF-1 mice;<br>Human ovarian cancer cell lines A2780 was purchased from Shanghai Zhongqiao Xinzhou Biotechnology Co., Ltd;<br>Human ovarian cancer cell lines SKOV3 was purchased from ATCC, Lot. 70025324;<br>Human ovarian cancer cell lines OVCAR3 was purchased from ATCC, Lot. HTB-161.
Authentication	A2780,SKOV3, and OVCAR3 were authenticated via short tandem repeat typing (STR).
Mycoplasma contamination	All cell lines were regularly tested for mycoplasma.
Commonly misidentified lines<br>(See ICLAC register)	No commonly misidentified lines were used in this study.

Animals and other research organisms

Policy information about studies involving animals; ARRIVE guidelines recommended for reporting animal research, and Sex and Gender in Research

Laboratory animals	Specific pathogen-free (SPF)-grade female BALB/c nude mice aged 4–6 weeks and weighing 14–16 g were purchased from the Vital River Laboratory Animal Technology Co., Ltd. (Beijing, China).
Wild animals	The study did not involve wild animals.
Reporting on sex	In our study design, considering that ovarian cancer only occurs in females, we chose female mice to establish the ovarian cancer model.
Field-collected samples	The study did not involve samples collected from the field.
Ethics oversight	AThe study protocol was approved by the Animal Ethics Committee of the First Hospital of Jilin University (Approval No. JDYY20250740). We have complied with all relevant ethical regulations for animal use. All housing conditions and experimental procedures strictly complied with the Guidelines for the Management of Laboratory Animals.

Note that full information on the approval of the study protocol must also be provided in the manuscript.

Plants

Seed stocks	*Report on the source of all seed stocks or other plant material used. If applicable, state the seed stock centre and catalogue number. If plant specimens were collected from the field, describe the collection location, date and sampling procedures.*
Novel plant genotypes	*Describe the methods by which all novel plant genotypes were produced. This includes those generated by transgenic approaches, gene editing, chemical/radiation-based mutagenesis and hybridization. For transgenic lines, describe the transformation method, the number of independent lines analyzed and the generation upon which experiments were performed. For gene-edited lines, describe the editor used, the endogenous sequence targeted for editing, the targeting guide RNA sequence (if applicable) and how the editor was applied.*
Authentication	*Describe any authentication procedures for each seed stock used or novel genotype generated. Describe any experiments used to assess the effect of a mutation and, where applicable, how potential secondary effects (e.g. second site T-DNA insertions, mosiacism, off-target gene editing) were examined.*
